# Cap control: cyclic *versus* linear oligomerisation in covalent template-directed synthesis†

**DOI:** 10.1039/c9ra07233k

**Published:** 2019-09-18

**Authors:** Diego Núñez-Villanueva, Maria Ciaccia, Christopher A. Hunter

**Affiliations:** Department of Chemistry, University of Cambridge Lensfield Road Cambridge CB2 1EW UK dn325@cam.ac.uk

## Abstract

Covalent template-directed synthesis was used to oligomerise monomer building blocks in a controlled manner to give exclusively the linear trimer. Competing reaction pathways were blocked by addition of a large excess of a monomeric capping agent. At a concentration of 1 mM, the cap selectively prevented further reaction of the product chain ends to give polymeric and macrocyclic products, but did not interfere with the templating process.

## Abstract 2

The right concentration of capping agent is required to control the product distribution in covalent template-directed synthesis of linear oligomers using CuAAC.

## Graphical abstract

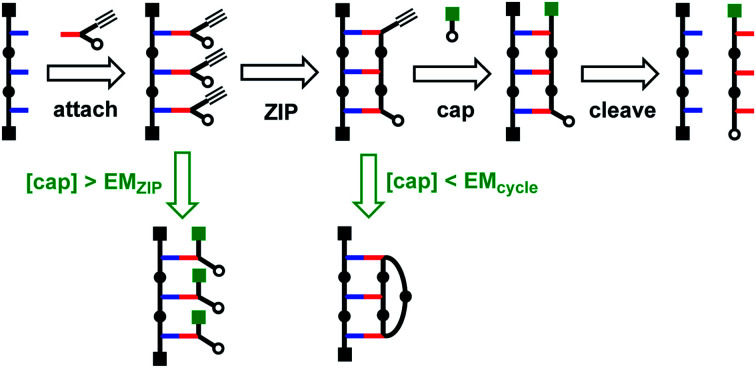

Biological information is encoded as a sequence of different monomer building blocks assembled into a linear polymer. Nucleic acids transfer this genetic information from one polymer template to another by using enzyme mediated template-directed synthesis.^1,2^ This unique property is the basis for molecular evolution in living systems.^3^ Synthetic oligomers that transfer sequence information in the same way could be used to develop evolutionary methods to search new regions of chemical space constituted by synthetic mixed sequence polymers. Programming molecular function using sequence is unlikely to be limited to biomolecules, but the challenge for synthetic systems is the development of approaches to identifying functional sequences, and molecular evolution is an attractive solution.^4^

In nucleic acids, template-directed information transfer is assisted by a complex enzymatic machinery that ensures the fidelity of the transfer.^5–7^ Non-enzymatic sequence information transfer is much more challenging, even using nucleic acids.^8–11^ We have been working on an alternative chemical method for the replication of molecular information in synthetic oligomers based on covalent base-pairing.^12^ Covalent templating has been used previously for the preparation of macrocycles, mechanically interlocked molecules, polydisperse homo-polymers and imprinted dendrimers.^13–26^ We have recently shown that this approach to sequence information transfer has a number of advantages over template-directed synthesis using dynamic covalent or non-covalent interactions, due to the degree of control that can be achieved in each step of the process. Fig. 1 illustrates the approach. In the first step, monomer building blocks are attached to the template using kinetically stable covalent bonds. In the ZIP step, the monomers are oligomerised on the template to give the duplex. After capping the terminal reactive sites on the duplex, the covalent base-pairs are cleaved to recover the template and the copy strand, which has a complementary sequence to the template. We have demonstrated the viability of this approach using ester bond formation between a phenol and a benzoic acid as the covalent base-pairing system and copper catalysed alkyne azide cycloaddition (CuAAC)^27,28^ for the ZIP reaction to form the oligomer backbone.^12^

**Fig. 1:**
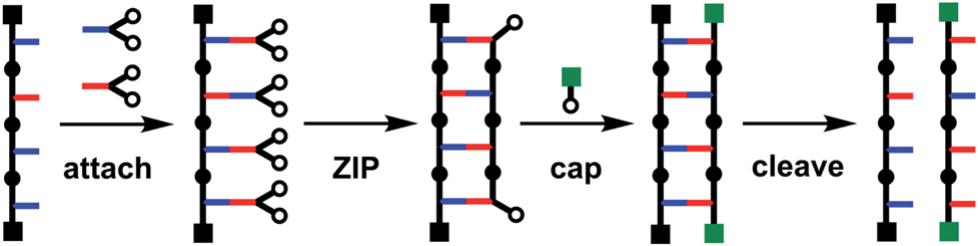
Covalent template-directed synthesis of a linear oligomer. In the attach step, monomers are coupled with complementary groups on the template. In the ZIP step, intramolecular reactions between complementary reaction sites on the monomers (white dots) lead to oligomerization. In the cap step, the reactive chain ends of the copy strand are blocked with monofunctional reagents. In the cleave step, the bonds connecting the new oligomer to the template are broken to regenerate the template and release the product.

The major challenge in covalent template-directed synthesis of linear oligomers is the ZIP step, which requires that coupling of adjacent monomer units on the template is highly favoured over all of the other possible inter- and intramolecular reactions. Fig. 2 illustrates the competing reaction pathways for a trimer template loaded with three monomer building blocks (1). In principle, it should be possible to favour intramolecular over intermolecular reaction pathways by working at high dilution conditions. However, the rate limiting step in the CuAAC reaction is formation of the copper–alkyne complex, which then reacts rapidly with the nearest available azide, so that dilution cannot be used to favour intramolecular processes.^29^ The solution is to carry out the ZIP reaction in the presence of a large excess of a monofunctional azide. Provided the concentration of the capping agent much higher than the concentration of unreacted terminal azide groups, it is possible to intercept the reactive alkyne group on the end of the templated oligomer in 2 and block formation of higher oligomers, such as 3 in Fig. 2.^29^ The concentration of the capping agent must also be much lower than the effective molarity for the intramolecular ZIP reaction, otherwise competition with the ZIP reaction will give rise to multiply capped species (4 and 5 in Fig. 2).^29^ We have shown that this capping strategy works well for dimer templates, but for longer templates, there are additional competing reaction pathways leading to macrocyclic products (6 in Fig. 2).^26^ Here we investigate the factors that control the yields of macrocyclic *versus* linear oligomers and show how a capping agent can be used to influence the product distribution and obtain the linear oligomer as the only major product of the ZIP reaction.

**Fig. 2:**
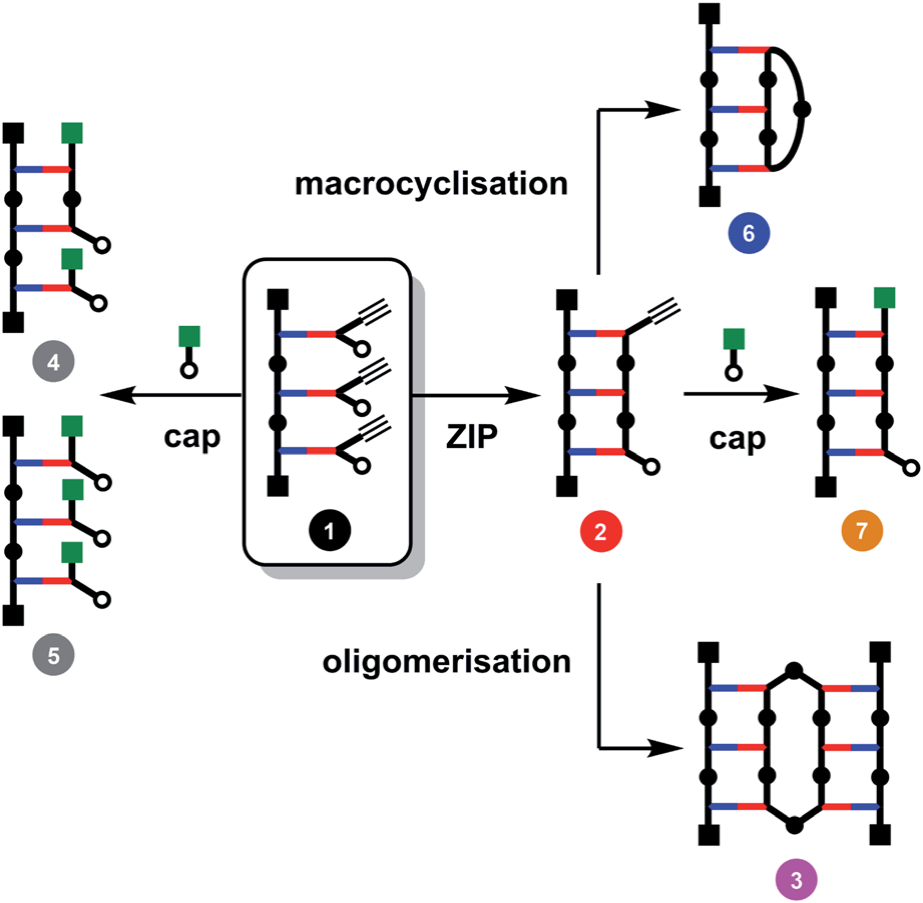
Competing reaction pathways in the ZIP step of covalent template-directed synthesis of a linear oligomer in the presence of a capping agent.

Scheme 1 shows the synthesis of a trimer template (12) *via* sequential CuAAC and deprotection reactions. First, monomers 8 and 9 were coupled using CuAAC to give the 2-mer 10. CuAAC coupling of 10 with capped 1-mer 11 followed by basic hydrolysis of the methyl ester groups afforded template 12 in good yield. From 12, ester coupling with phenol monomer 13 gave the pre-ZIP intermediate 1.

**Scheme 1:**
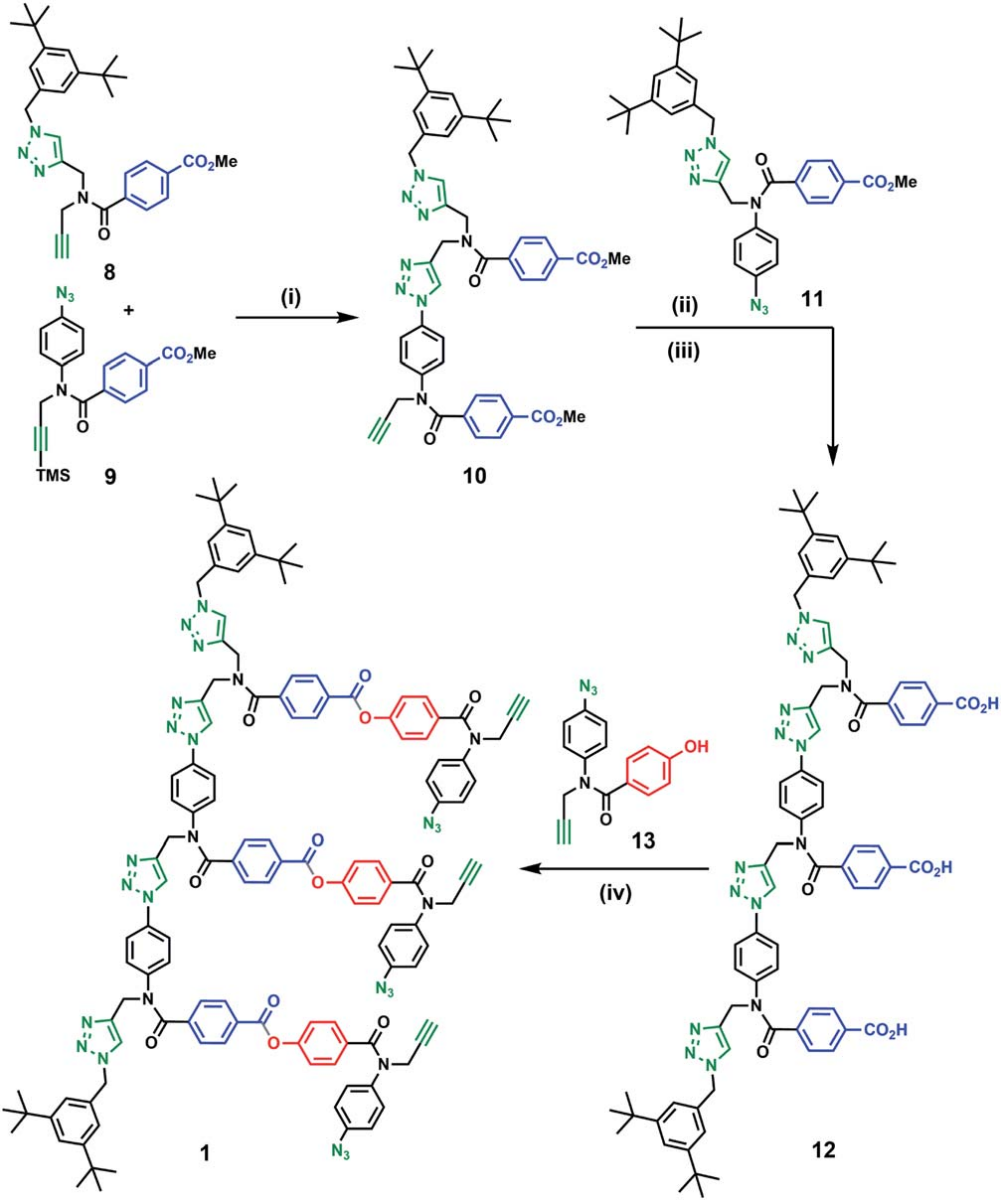
Synthesis of pre-ZIP intermediate 1. (i) Cu(CH_3_CN)_4_PF_6_, TBTA in THF then 1 M TBAF in THF (quant.); (ii) 11, Cu(CH_3_CN)_4_PF_6_, TBTA in THF (84%); (iii) LiOH in THF : H_2_O (quant.); (iv) 13, EDC, DMAP in CH_2_Cl_2_ (59%).

When the monomer building blocks were oligomerised in an untemplated reaction using Cu-TBTA, the major products obtained were the macrocyclic trimer and the macrocyclic tetramer.^29^ Macrocyclic dimers were never observed, because this ring is highly strained. Therefore the trimer template is the simplest system in which macrocyclization competes with the other reaction pathways shown in Fig. 2. When 1 was treated with Cu-TBTA under dilute conditions with no capping agent present, the desired duplex 2 was formed rapidly. However, 2 was consumed by reaction of the terminal azide and alkyne groups in an intermolecular fashion to give higher order oligomer 3 and an intramolecular fashion to give macrocycle 6 (Fig. 3(a)). After 2 days, 3 and 6 were the only products observed in the reaction mixture. Fig. 3(a) shows that the rates of formation and the yields of 3 and 6 are similar.

**Fig. 3:**
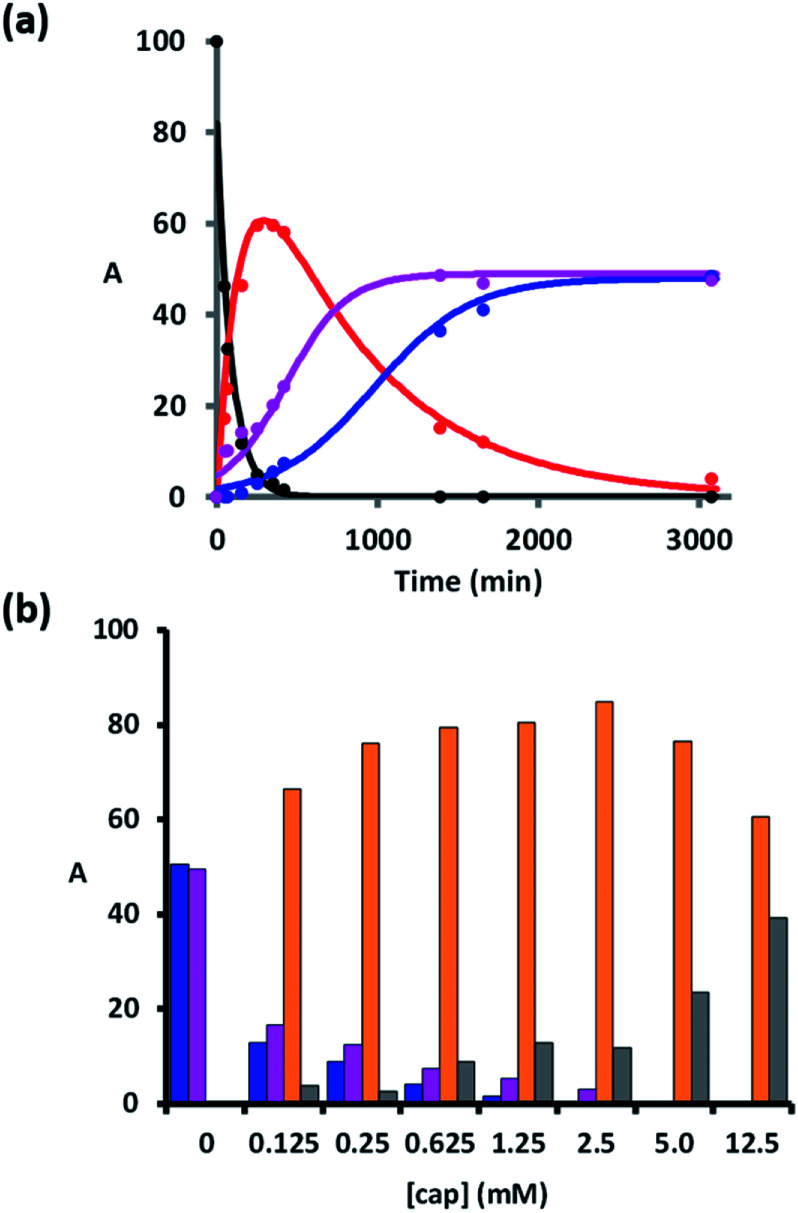
(a) Time dependence of the product distribution for the reaction of 1 (25 μM) and Cu-TBTA (50 μM) in THF at room temperature. The solid lines are drawn as a guide. (b) Product distribution of the CuAAC reaction of 1 (25 μM) using CuTBTA (50 μM) in THF at room temperature in the presence of different concentrations of 4-*t*-butylbenzyl azide. The areas of the UPLC peaks assigned to 1 (black), 2 (red), 3 (pink), multiple capped products 4 and 5 (grey), 6 (blue), and 7 (orange) are plotted as a percentage of the total of all peak areas (A) measured using the UV/vis absorption at 280 nm.

We therefore investigated the use of a monofunctional capping agent (4-*t*-butylbenzyl azide) to compete with these undesired reaction pathways. The product distribution obtained in a presence of different concentrations of 4-*t*-butylbenzyl azide is shown in Fig. 3(b). Millimolar concentrations of capping azide effectively compete with both the intramolecular macrocyclisation reaction and the intermolecular oligomerisation reaction and give the capped duplex 7 as the major product. At higher concentrations, the capping azide starts to compete with the intramolecular ZIP reaction leading to multiply capped products (4 and 5). The sweet spot is at a concentration of capping agent around 1 mM, which results in almost quantitative yield of the capped duplex 7 (90%). The backbone shown in Scheme 1 is directional so all duplex products could have a parallel or antiparallel backbone. Based on the effective molarities measured previously for dimer templates, the proportion of parallel product is expected to be minimal at this concentration of capping azide.^29^ However, the ^1^H NMR spectrum of the duplex is complicated, and there is too much signal overlap to discern whether there is more than one isomer of the duplex present (see ESI†).

When the product obtained from the ZIP reaction carried out in the absence of any capping agent was subjected to basic hydrolysis, a mixture of the macrocyclic phenol trimer and hexamer was obtained together with the original acid trimer template 12 (Fig. 4(a)). In contrast, hydrolysis of the product obtained by carrying out the ZIP reaction in the presence of 1.25 mM capping agent gave the linear phenol trimer 14 as the only major product (Fig. 4(b)). The UPLC yields for these steps are high, but the isolated yields after chromatography were lower (70% and 50% for the ZIP and cleave steps respectively for reactions carried out on the 10 mg scale). Fig. 4(c) shows the mass spectrum of the isolated linear trimer, which was fully characterized (see ESI†).

**Fig. 4:**
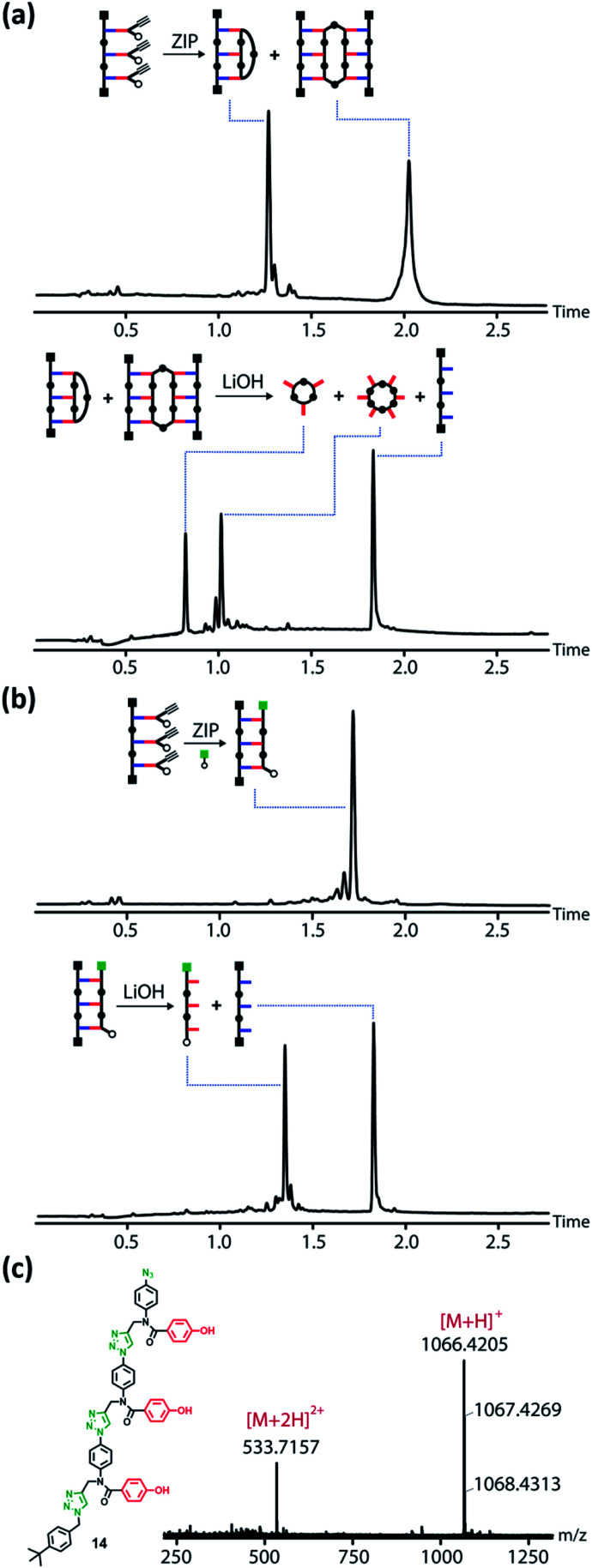
(a) UPLC traces of the products of the CuAAC reaction of 1 with no capping azide present (top), and the products of the subsequent basic hydrolysis (bottom). (b) UPLC traces of the products of the CuAAC reaction of 1 with 1.25 mM of capping azide (top), and the products of the subsequent basic hydrolysis (bottom). (c) Chemical structure and HRMS of the copy strand 14 (calculated mass for [M + H]^+^ = 1066.4225).

The product distributions obtained in these template-directed reactions depend on the concentration of chain end azide, the concentration of capping azide, and the effective molarities of the intramolecular ZIP reaction (EM_ZIP_) and the intramolecular macrocyclisation reaction (EM_cycle_). The data in Fig. 3(b) can therefore be used to determine the values of EM for the intramolecular reactions. The EM for macrocyclisation of 2 to give 6 was determined by extrapolating to the concentration of capping agent required to obtain a 1 : 1 ratio of the intramolecular macrocyclic (6) and intermolecular capped duplex (7) product (Fig. 5), and correcting for the 5-fold difference in reactivity previously measured for the aromatic and aliphatic azides.^29^ The EM for macrocyclisation is 0.14 mM, which is three orders of magnitude lower than the value of EM that we have previously measured for the intramolecular ZIP reaction (530 mM).^29^

**Fig. 5:**
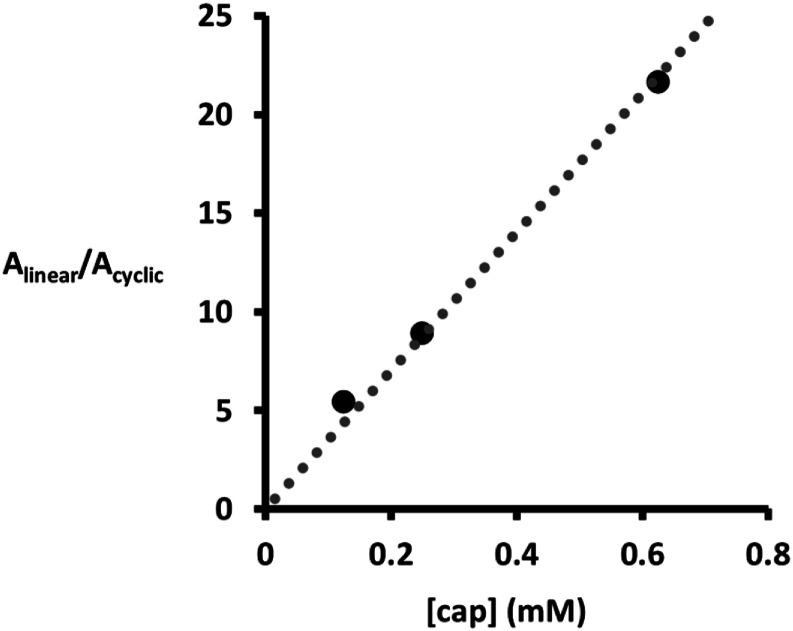
Product distribution for the CuAAC reaction of 1 (25 μM) using CuTBTA (50 μM) in THF at room temperature in the presence of different concentrations of capping 4-*t*-butylbenzyl azide, plotted as the ratio of the area of the UPLC peaks assigned to the capped duplex 7 compared with the area of the UPLC peaks assigned to the macrocycle 6. Peak areas were measured using the UV/vis absorption at 280 nm. The line represents the best fit of the data to *A*_linear_/*A*_cyclic_ = *c* [cap], where *c* is a constant related to EM_cycle_ for the intramolecular process and is equal to 35 (*R*^2^ = 0.99).

It is this large difference in EM values that allowed us to find the sweet spot in the concentration of capping agent. A high yield in the template-directed synthesis of linear oligomers requires that EM_ZIP_ ≫ [cap] and EM_cycle_ ≪ [cap]. This condition is only satisfied when the two EM values are different by more than two orders of magnitude. In untemplated reactions, the macrocyclic trimer was the major product, which implies that the EM for formation of larger macrocycles is likely to be lower than 0.14 mM. The oligomer architecture shown in Scheme 1 therefore appears to be well-suited to template-directed synthesis of longer oligomers, because it should be possible to control all competing processes in the ZIP step by using a 1 mM concentration of capping azide.

In conclusion, we have developed a capping strategy for efficient covalent template-directed synthesis of linear oligomers using CuAAC. The key for the success of this process is that monomers covalently attached to the template react with adjacent monomers to yield a linear daughter strand of the same length of the template. The product duplex carries two reactive groups on the chain ends which can lead to intermolecular oligomerisation processes or intramolecular macrocyclisation, compromising the yield of the templated reaction. In the system described here, the EM for macrocyclization is three orders of magnitude lower than the EM for the intramolecular ZIP reaction, so it is possible to completely block all undesired side reactions by using 1 mM of a capping azide to intercept all of the reactive chain ends without interfering with the templated oligomerisation.

## Conflicts of interest

There are no conflicts to declare.

## Supplementary Material

RA-009-C9RA07233K-s001
