# Supplementary material for: Cap control: cyclic versus linear oligomerisation in covalent template-directed synthesis
Source: RSC Adv. 2019 Sep 18;9(51):29566–9. doi: 10.1039/c9ra07233k (PMC9071899; doi:10.1039/c9ra07233k)
Supplement: RA-009-C9RA07233K-s001 [file RA-009-C9RA07233K-s001.pdf]

Electronic Supplementary Material (ESI) for RSC Advances.
This journal is © The Royal Society of Chemistry 2019

Supporting information

Cap control: cyclic versus linear oligomerisation in covalent template-directed synthesis

Diego Núñez-Villanueva, Maria Ciaccia and Christopher A. Hunter*

Department of Chemistry, University of Cambridge, Lensfield Road, Cambridge CB2 1EW, UK. E-mail: herchelsmith.orgchem@ch.cam.ac.uk

TABLE OF CONTENTS **Page**

**General experimental details.**	S2
**CuAAC reaction of pre-ZIP 1 without capping azide.**	S3
**CuAAC reaction of pre-ZIP 1 in the presence of the 4-*t*-butylbenzyl azide.**	S9
**Synthesis and characterization of described compounds.**	S16
**References.**	S59

General experimental details.

All the reagents and materials used in the synthesis of the compounds described below were bought from commercial sources, without prior purification. Dry THF and CH_2Cl_2 were obtained from a solvent purification system (Pure Solv™, Innovative Technology, Inc.). Anhydrous DMF was purchased from Sigma-Aldrich. Thin layer chromatography was carried out using with silica gel 60F (Merck) on glass plates. Flash chromatography was carried out on an automated system (Combiflash Rf+ or Combiflash Rf Lumen) using prepacked cartridges of silica (25µ PuriFlash® columns). All NMR spectroscopy was carried out on a Bruker 400 MHz DPX400, 400 MHz AVIII400, 500 MHz DCH cryoprobe or 500 MHz TCI Cryoprobe spectrometer using the residual solvent as the internal standard. All chemical shifts (δ) are quoted in ppm and coupling constants given in Hz. Splitting patterns are given as follows: s (singlet), bs (broad singlet), d (doublet), t (triplet), q (quadruplet), m (multiplet). FT-IR spectra were measured on a PerkinElmer Spectrum One spectrometer equipped with an ATR cell. Melting points were measured in a Mettler Toledo MP50 Melting Point System. UPLC analysis of samples was performed using Waters Acquity H-class UPLC coupled with a single quadrupole Waters SQD2. ACQUITY UPLC CSH C18 Column, 130Å, 1.7 µm, 2.1 mm X 50 mm was used as the UPLC column. The conditions of the UPLC method are as follows: gradients of water +0.1% formic acid (solvent A) and acetonitrile +0.1% formic acid (solvent B=) as specified in each case. Flow rate: 0.6 ml/min; Column temperature of 40°C; Injection volume of 2 µL. The signal was monitored at 254 nm. HRMS analysis was performed in a Waters LCT Premier equipped with a TOF mass analyser and W optics for enhanced resolution, using 50% aqueous acetonitrile with 0.25% formic acid as mobile phase.

CuAAC reaction of pre-ZIP 1 without capping azide.

Scheme S1 shows the possible products for the CuAAC reaction of pre-ZIP **1** performed at high dilution in the absence of capping azide. Firstly, the linear duplex **2** must be formed both in the parallel and antiparallel arrangement as the backbone is directional. Then, further CuAAC reaction of **2** would lead to cyclic 3-mer duplex **6** and intermolecular adducts such as **3**.

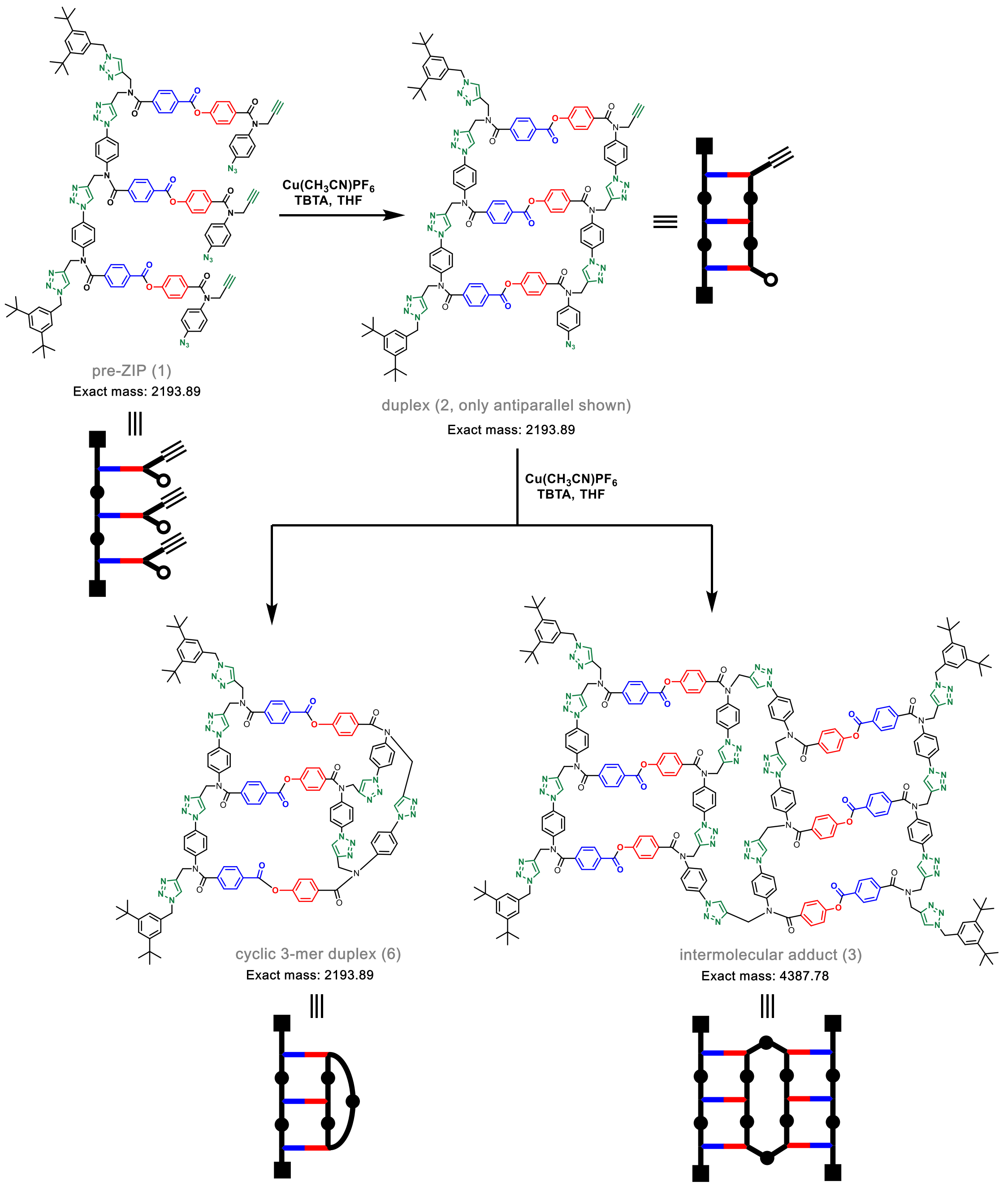

Scheme S1.

The reaction was performed as follows: Compound **1** was dissolved, under N_2 atmosphere, in dry THF in 25 μM concentration. A stock solution of $Cu(CH_3CN)_4PF_6$ and TBTA in THF was prepared and the volume needed to get 50 μM concentration of Cu-TBTA was added to the reaction. The reaction was stirred at room temperature under N_2 atmosphere. Aliquots of the

reaction were taken at different times, diluted with EtOAc and washed with 0.2 M EDTA soln. and then analysed by UPLC to assess the progress of the reaction (Figure S1). In the first hours of reaction, linear 2-mer and linear 3-mer duplexes are detected (Figure S2). After 2 days of reaction, these linear duplexes are consumed and a mixture of cyclic duplex **6** and intermolecular adduct **3** is formed as they accumulate.

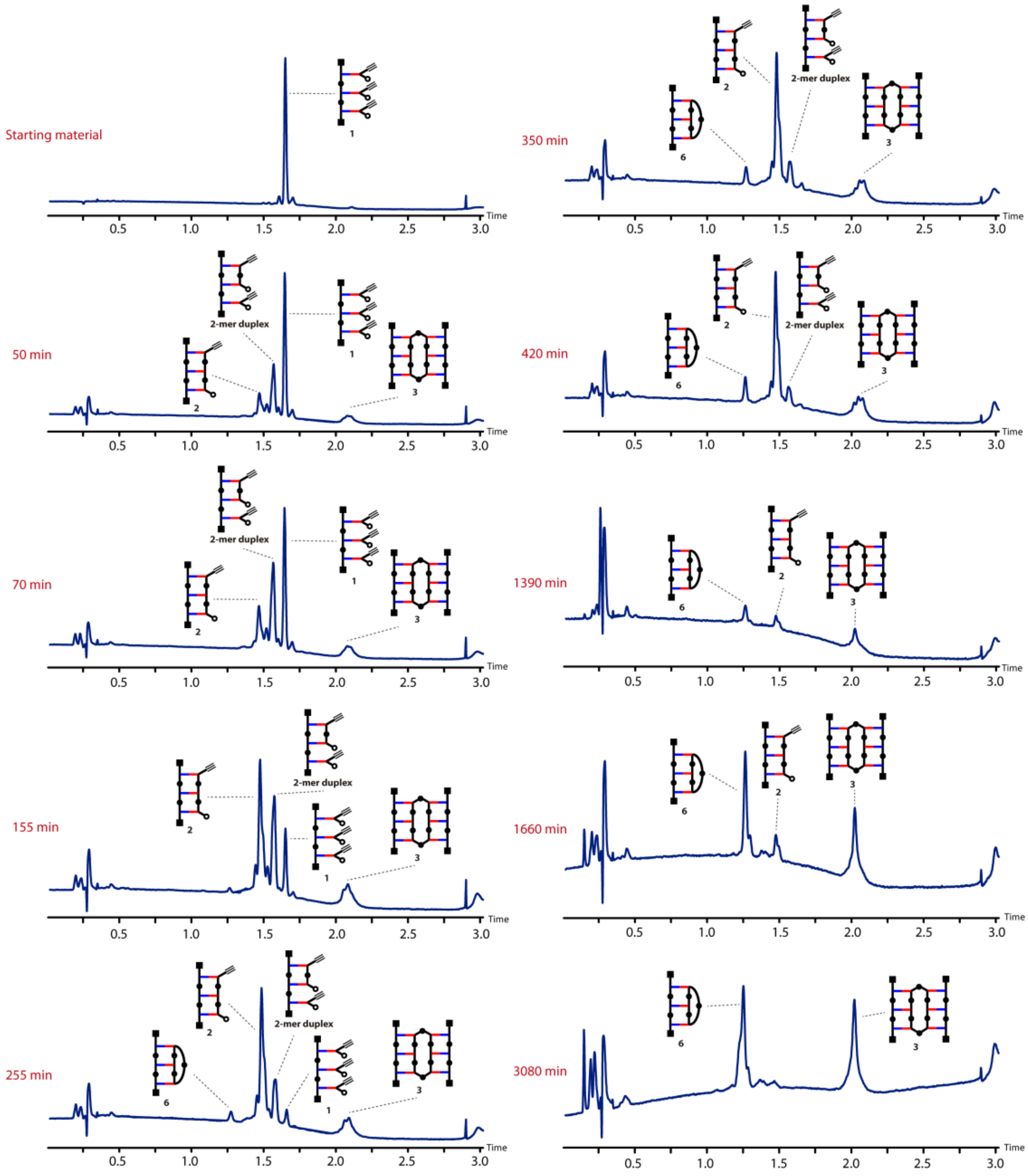

Figure S1. UPLC traces for the CuAAC reaction of **1** (50 µM of **1** and Cu-TBTA in THF) at different times. *Conditions:* C18 column at 40 °C (254 nm) using water + 0.1% formic acid (A) and CH_3CN + 0.1% formic acid (B) with flow rate of 0.6 ml/min; Gradient of 0-2 minutes 65% -100%B + 1 minute 100% B.

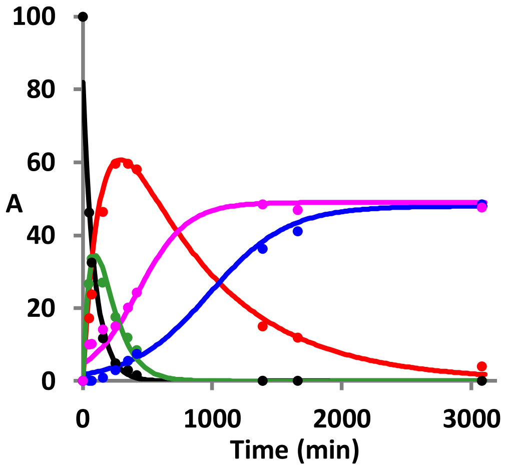

Figure S2. Time dependence of the product distribution for the reaction of **1** (25 µM) and Cu-TBTA (50 µM) in THF at room temperature (black = **1**, green=**linear 2-mers**, red = **2**, pink = **3**, blue = **6** , plotted as HPLC peak area **A**). The solid lines are drawn as a guide.

Isolation of compound **6** (Figure S3A and S3B) provided key evidences of the formation of a cyclic product: the absence of acetylene protons in ^{1}H NMR and azide stretching bands in IR, when compared to the starting material **1** (Figure S3C and S3D). Compound **3** was assigned by comparison to the 2-mer analogue[S1] and the product distribution after ester hydrolysis (Figure S4).

Basic hydrolysis of the crude mixture (mixture of **6** and **3**) and isolated **6** proved to be an alternative and efficient way to analyse the reaction. As shown in Scheme S2, basic hydrolysis of the crude mixture provided a mixture of cyclic 3-mer **15** and cyclic 6-mer **16** together with recovered template **12** (Figure S4) while, from isolated **6**, the hydrolysis crude contained only cyclic 3-mer **15** and template **12** (Figure S5).

The reaction was performed as follow: In the case of the hydrolysis of the ZIP reaction crude, once the reaction is finished, the solution was diluted with EtOAc, washed with 0.2 M EDTA soln. and the solvent evaporated. The crude was dissolved in THF (1 mL) and 1M LiOH soln. (3-4 drops) was added. After 15 min of reaction at room temperature, the reaction was quenched with diluted HCl soln., extracted with EtOAc and analyse by UPLC. The same hydrolysis procedure was followed to hydrolyse compound **6**, affording cyclic **15**. Full procedure and characterization of **15** can be found in pages S53-S55.

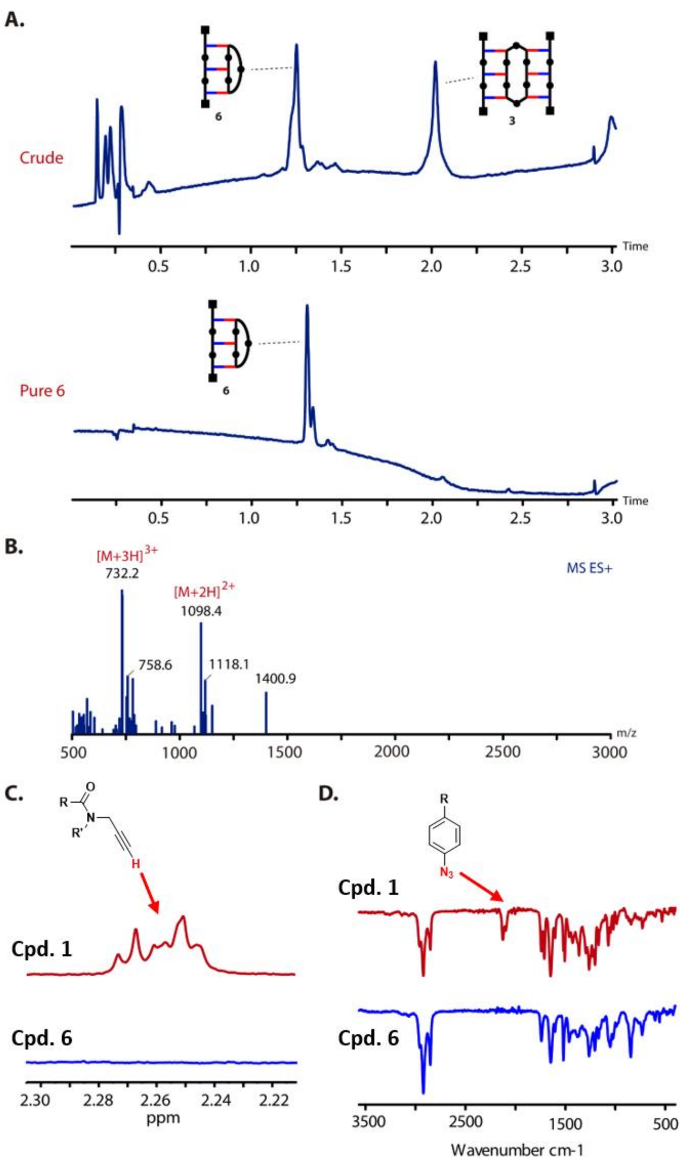

Figure S3. A. UPLC traces of the crude mixture from the reaction of **1** (50 µM) with Cu-TBTA in THF, and isolated **6**. *Conditions:* C18 column at 40 °C (254 nm) using water + 0.1% formic acid (A) and CH_3CN + 0.1% formic acid (B) with flow rate of 0.6 ml/min; Gradient of 0-2 minutes 65% -100%B + 1 minute 100% B. **B.** Mass spectra of **6**. **C.** Partial ^1H NMR (400 MHz, $CDCl_3$) of **1** (top) and **6** (bottom), showing the absence of acetylene protons in **6**. **D.** FT-IR (ATR) of **1** (top) and **6** (bottom), showing the absence of azide stretching band in **6**.

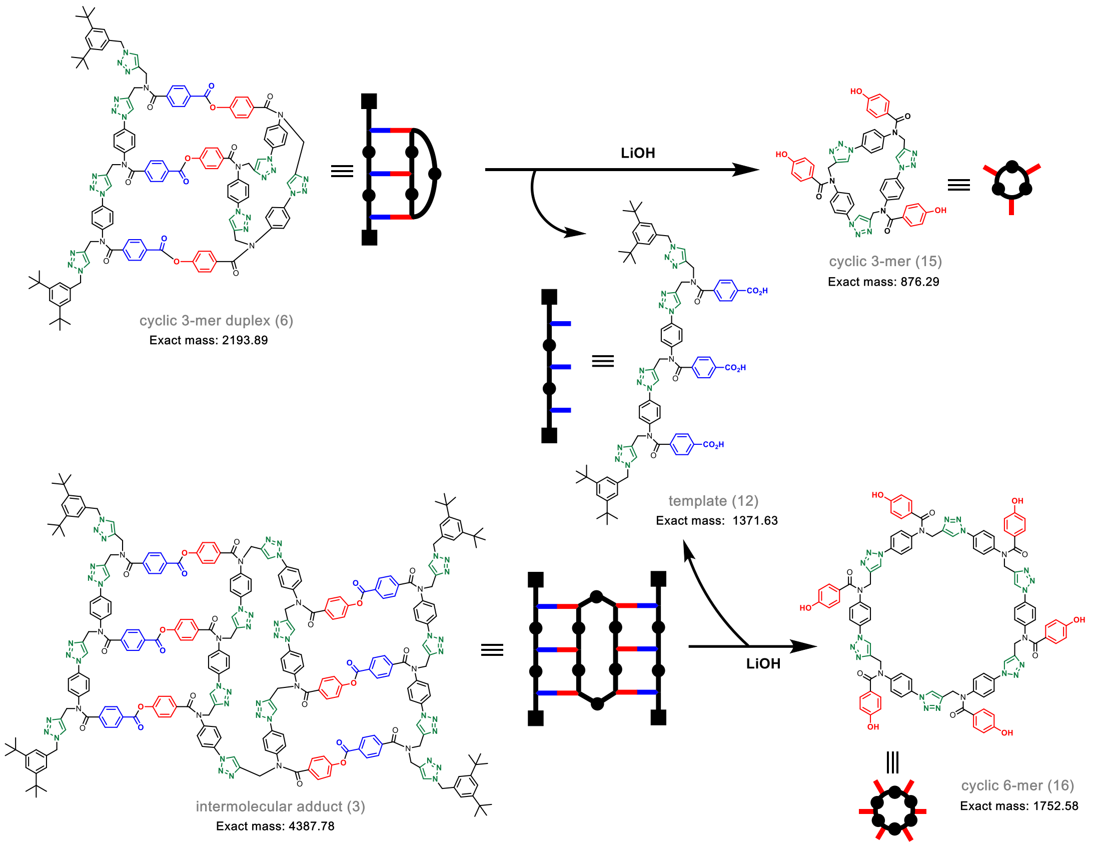

Scheme S2.

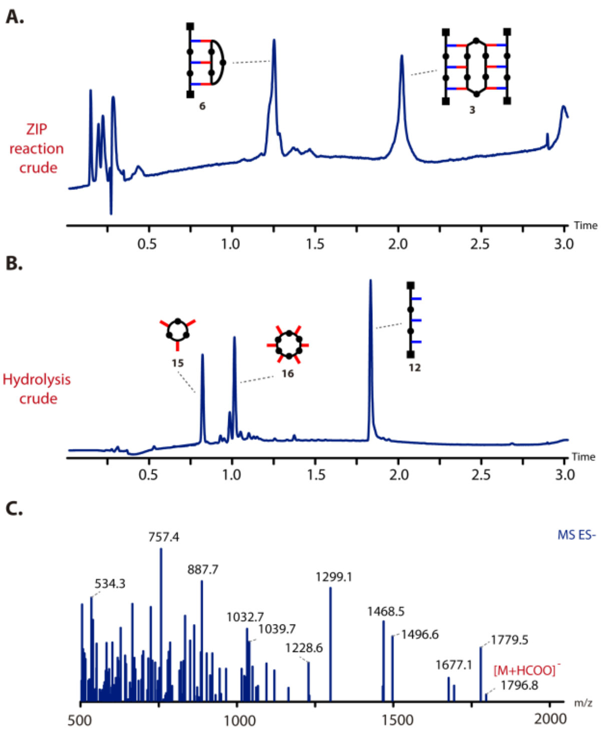

Figure S4. A. UPLC trace of the crude mixture from the ZIP reaction. **B.** UPLC trace for the hydrolysis of the crude mixture shown in **A**. *Conditions:* C18 column at 40 °C (254 nm) using water + 0.1% formic acid (A) and CH_3CN + 0.1% formic acid (B) with flow rate of 0.6 ml/min; Gradient of 0-2 minutes 65% -100%B + 1 minute 100% B (for **A**) and 0-2 minutes 5% -100%B + 1 minute 100% B (for **B**). **C.** Mass spectra of **16**.

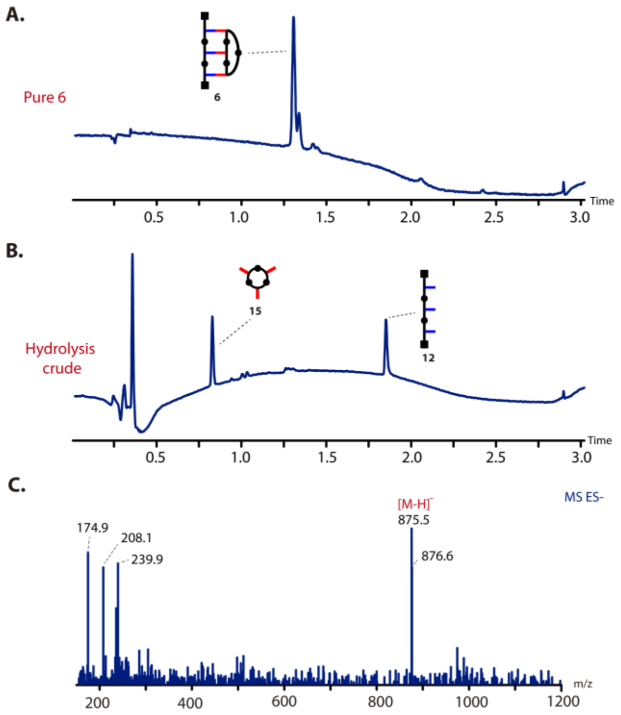

Figure S5. A. UPLC traces of the pure isolated **6**. **B.** UPLC trace for the hydrolysis of the **6**. *Conditions:* C18 column at 40 ºC (254 nm) using water + 0.1% formic acid (A) and CH_3CN + 0.1% formic acid (B) with flow rate of 0.6 ml/min; Gradient of 0-2 minutes 65% -100%B + 1 minute 100% B (for **A**) and 5% -100%B + 1 minute 100% B (for **B**). **C.** Mass spectra of **15**.

CuAAC reaction of pre-ZIP 1 in the presence of the 4-*t*-butylbenzyl azide.

Scheme S3 shows the possible products for the CuAAC reaction of pre-ZIP **1** performed at high dilution in the presence of different amounts of 4-*t*-butylbenzyl azide as capping agent. Firstly, the linear duplex **2** must be formed both in the parallel and antiparallel arrangement as the backbone is directional. Then, capping of **2** would lead to linear 3-mer duplex **7**. When the concentration of capping azide is high enough compared to the value of EM for the ZIP reaction, then doubly products (**4**) are formed.

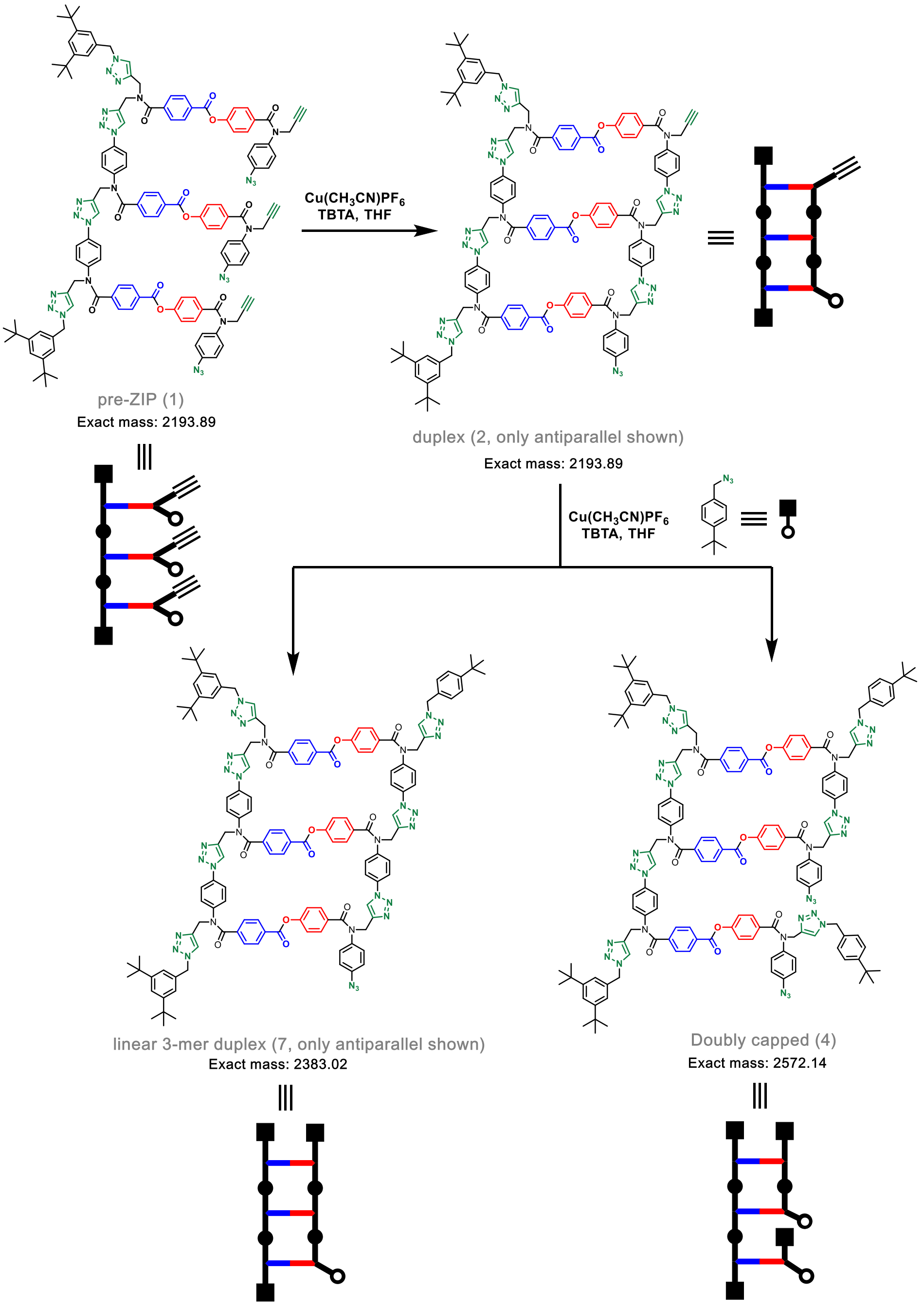

Scheme S3.

Initially, a 2.5 mM concentration of cap azide (100 equivalents) was used to analyse the reaction. The reaction was performed as follow: Compound **1** was dissolved, under N_2 atmosphere, in dry THF at 25 µM concentration. A stock solution 4-*t*-butylbenzyl azide in THF was prepared and the volume needed to get 2.5 mM concentration of cap was added to the reaction. Similarly, a stock solution of $Cu(CH_3CN)_4PF_6$ and TBTA in THF was prepared and the volume needed to get 50 µM concentration of Cu-TBTA was added to the reaction. The reaction was stirred at room temperature under N_2 atmosphere. Aliquots of the reaction were taken at different times, diluted with EtOAc and washed with 0.2 M EDTA soln. and then analysed by UPLC to assess the progress of the reaction (Figure S6). In the first hours of reaction, linear 2-mer and linear 3-mer duplexes are detected (Figure S7) but they are rapidly converted into capped linear 3-mer duplex **7**. After 2 days of reaction, **7** is the main product observed in the crude mixture. Due to the directionality of the backbone and residual CuAAC reaction between terminal monomers, the two satellites of **7** have been assigned as the parallel and 1,3-adduct.[S2]

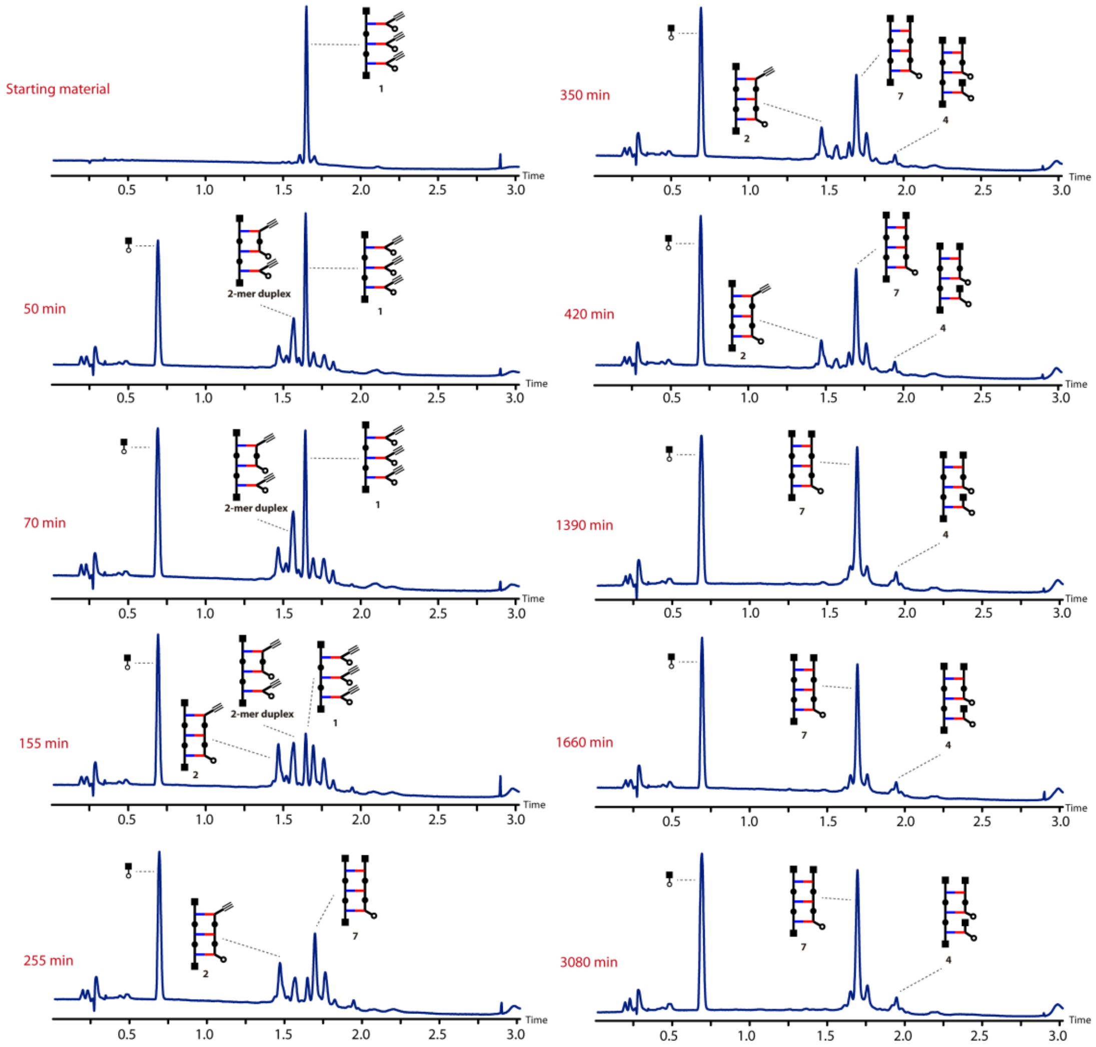

Figure S6. UPLC traces for the CuAAC reaction of **1** (50 µM of **1** and Cu-TBTA in THF) in the presence of *p*-*t*-butylbenzyl azide (2.5 mM) at different times. *Conditions:* C18 column at 40 °C (254 nm) using water + 0.1% formic acid (A) and CH_3CN + 0.1% formic acid (B) with flow rate of 0.6 ml/min; Gradient of 0-2 minutes 65% -100%B + 1 minute 100% B.

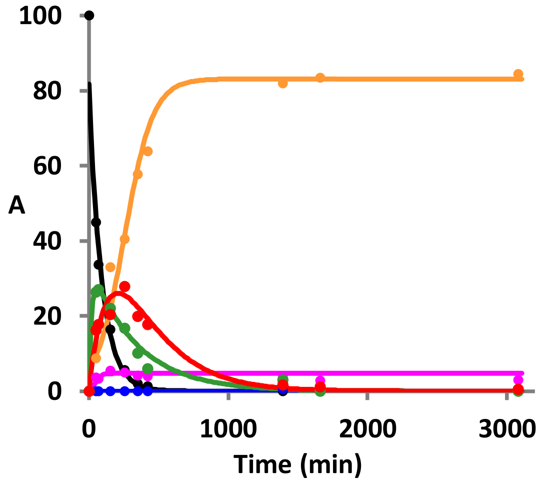

Figure S7. Time dependence of the product distribution for the reaction of **1** (25 µM), Cu-TBTA (50 µM) and 4-*t*-butylbenzyl azide (2.5 mM) in THF at room temperature (black = **1**, green=**linear 2-mers**, red = **2**, pink = **3**, blue = **6**, orange = **7**, plotted as HPLC peak area **A**). The solid lines are drawn as a guide.

Compound **7** was isolated (Figure S8A and S8B) and fully characterised by ^{1}H NMR, ^{13}C NMR, IR and HRMS (see pages S50-S52). Key evidence of the formation of a linear product was the absence of acetylene protons in ^{1}H NMR when compared to the starting material **1** and the existence of azide stretching bands in IR (Figure S8C and S8D).

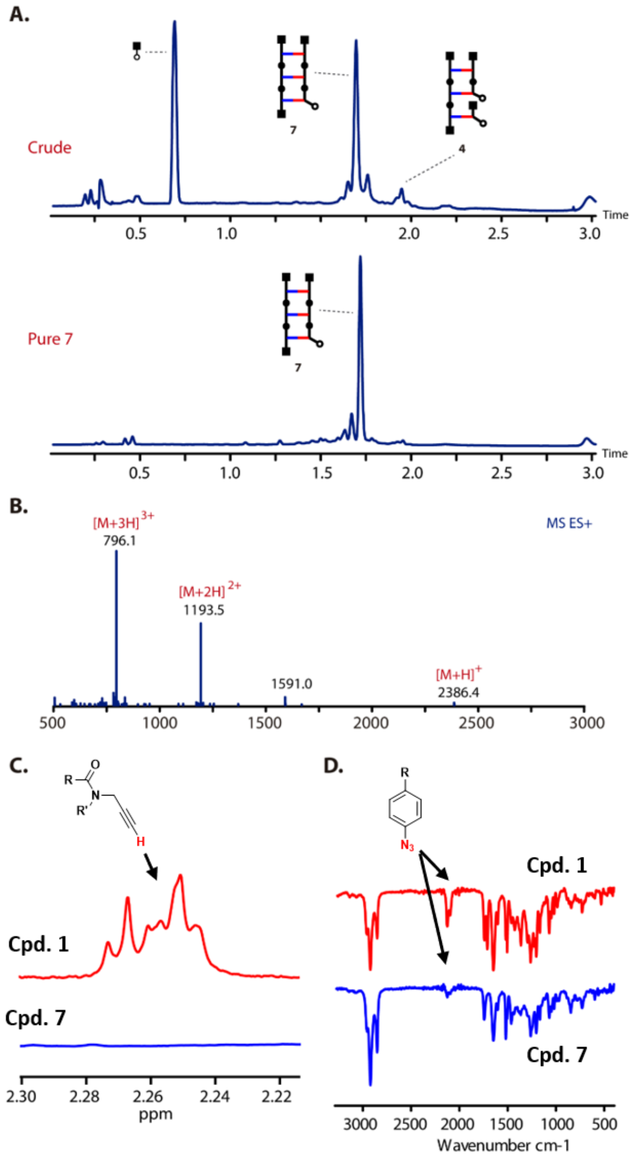

Figure S8. A. UPLC traces of the crude reaction mixture and isolated **7**. *Conditions:* C18 column at 40 °C (254 nm) using water + 0.1% formic acid (A) and CH_3CN + 0.1% formic acid (B) with flow rate of 0.6 ml/min; Gradient of 0-2 minutes 65% -100%B + 1 minute 100% B. **B.** Mass spectra of **7**. **C.** Partial ^{1}H NMR (400 MHz, $CDCl_3$) of **1** (top) and **7** (bottom), showing the absence of acetylene protons in **7**. **D.** FT-IR (ATR) of **1** (top) and **7** (bottom), showing the existence of azide stretching band in **1** and **7**.

In order to investigate the effect of the concentration of 4-*t*-butylbenzyl azide in the product distribution, the CuAAC reaction of **1** (50 μM of **1** and Cu-TBTA in THF) was performed using a range of capping azide concertation (0, 0.125, 0.25, 0.625, 1.25, 2.5, 5.0 and 12.5 mM). The crude reaction mixture after 2 days of stirring at room temperature was analysed by UPLC (Figure S9 and Figure 2(b), main text).

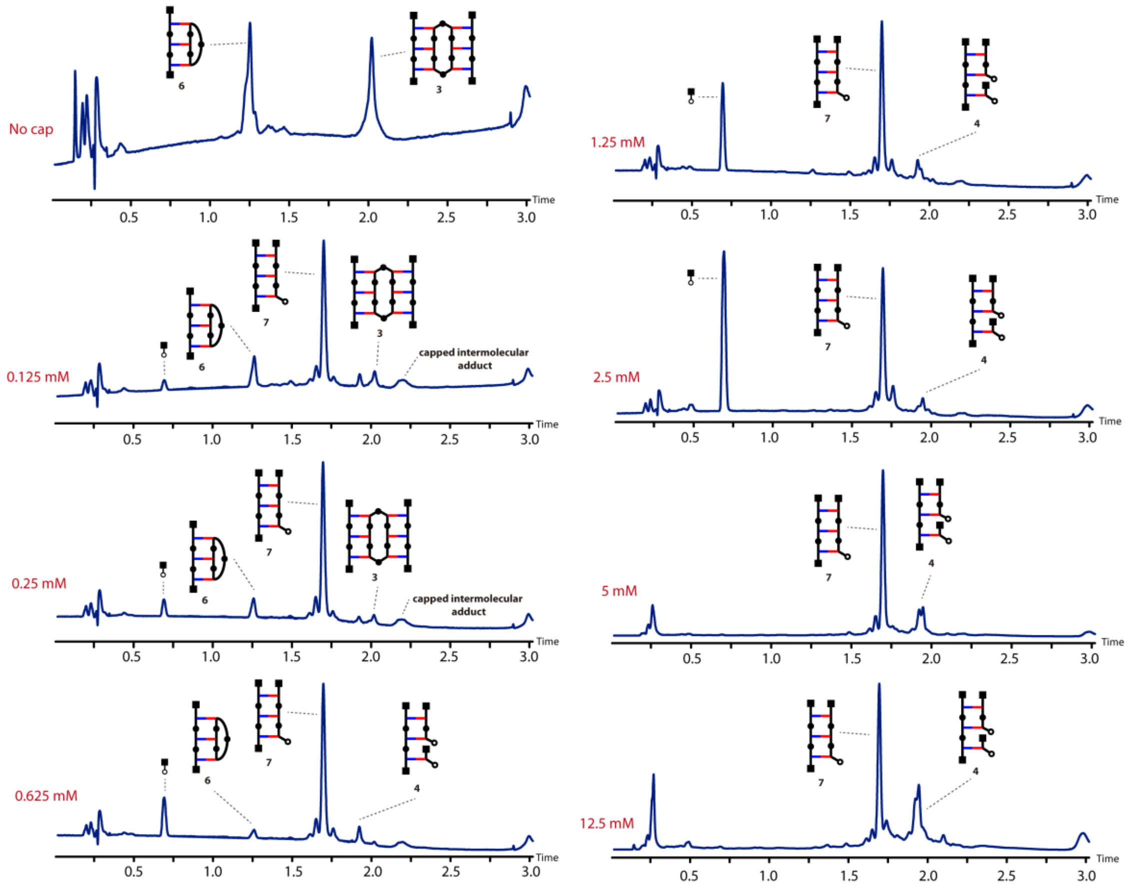

Figure S9. UPLC traces of the crude reaction mixture for the CuAAC reaction of **1** (50 µM of **1** and Cu-TBTA in THF) in the presence of *p*-*t*-butylbenzyl azide at different concentrations. *Conditions:* C18 column at 40 °C (254 nm) using water + 0.1% formic acid (A) and CH_3CN + 0.1% formic acid (B) with flow rate of 0.6 ml/min; Gradient of 0-2 minutes 65% -100%B + 1 minute 100% B. For the 5 mM and 12.5 mM experiment, the crude was dried under vacuum for 3 h to remove the excess of capping azide.

Basic hydrolysis of the crude mixtures for the experiments performed with 2.5 mM and 12.5 mM concentration of capping azide proved to be an alternative and efficient way to analyse the reaction. As shown in Scheme S4, for [cap]=2.5 mM, basic hydrolysis of the crude mixture provided mainly linear 3-mer **14** together with recovered template **12** (Figure S10). Linear 3-mer **14** was isolated and fully characterised by ^{1}H NMR, ^{13}C NMR, IR and HRMS (see pages S56-S58). For [cap]=12.5 mM, the hydrolysis crude contained linear 3-mer **14**, template **12** and a considerable amount of linear 1-mer and 2-mer (Figure S11).

The reaction was performed as follow: The crude from the CuAAC reaction was dissolved in THF (1 mL) and 1M LiOH soln. (3-4 drops) was added. After 15 min of reaction at room temperature, the reaction was quenched with diluted HCl soln., extracted with EtOAc and analyse by UPLC.

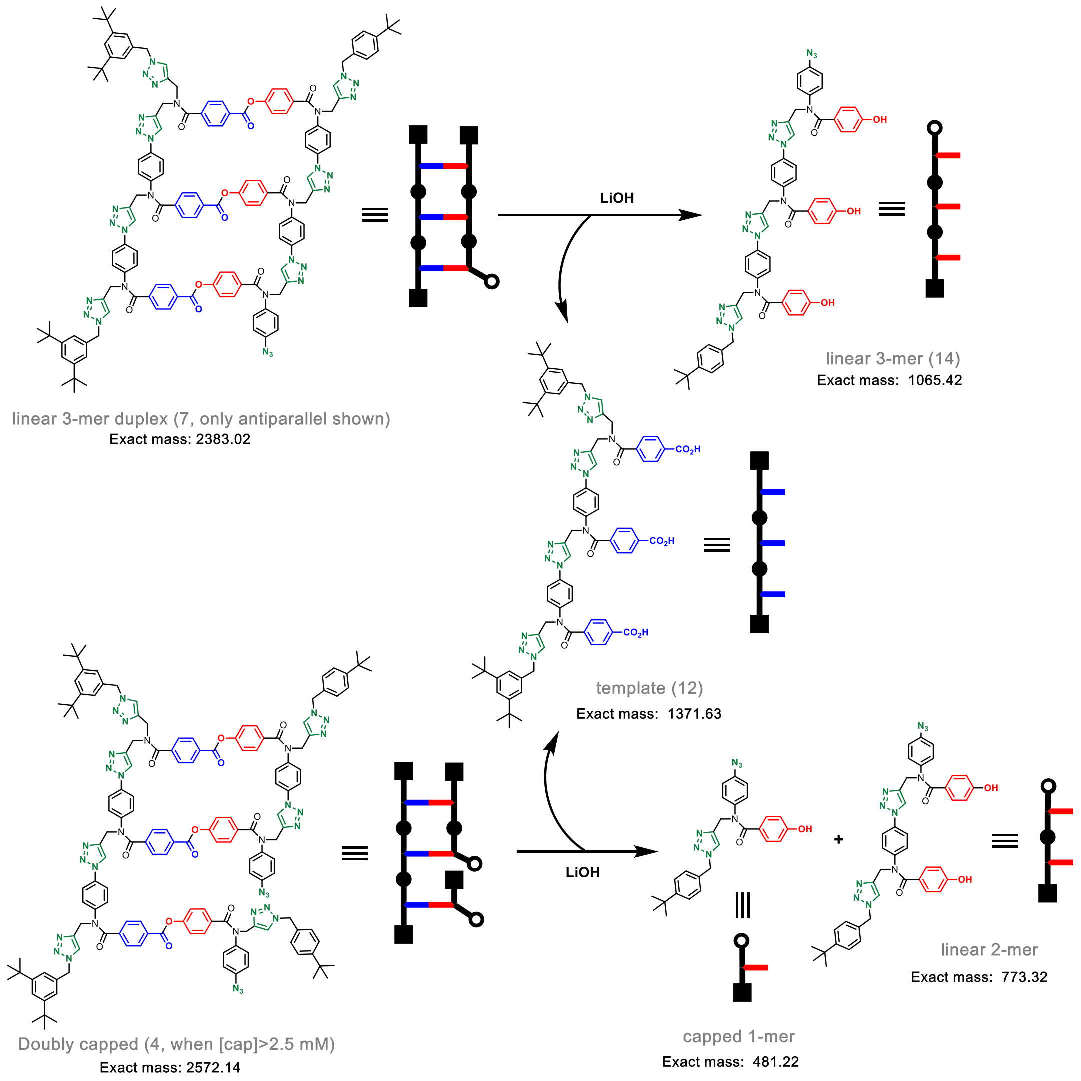
LiOH
linear 3-mer duplex (7, only antiparallel shown)
Exact mass: 2383.02
linear 3-mer (14)
Exact mass: 1065.42
template (12)
Exact mass: 1371.63
LiOH
Doubly capped (4, when [cap]>2.5 mM)
Exact mass: 2572.14
capped 1-mer
Exact mass: 481.22
linear 2-mer
Exact mass: 773.32

Scheme S4.

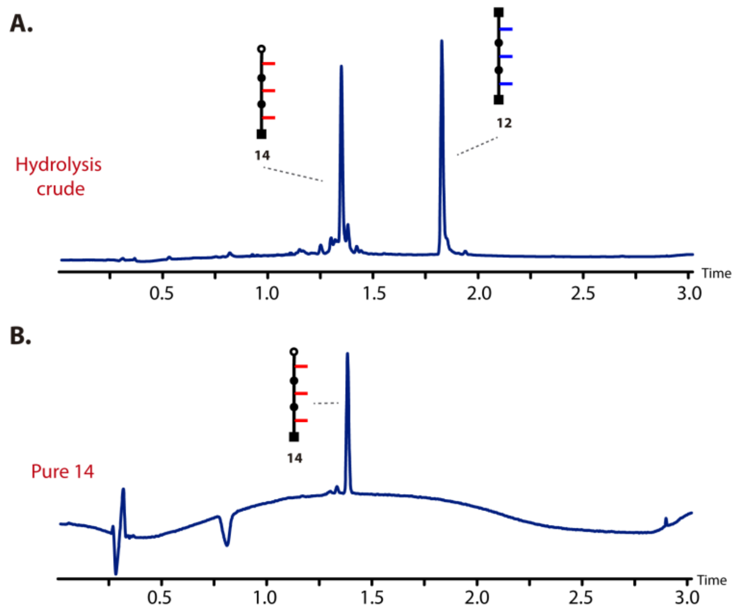

Figure S10. UPLC traces of the crude mixture after hydrolysis of isolated **7** (**A**) and isolated **14** (**B**). HRMS of **14** is shown in Figure 4, main text. *Conditions:* C18 column at 40 °C (254 nm) using water + 0.1% formic acid (A) and CH_3CN + 0.1% formic acid (B) with flow rate of 0.6 ml/min; Gradient of 0-2 minutes 5% -100%B + 1 minute 100% B.

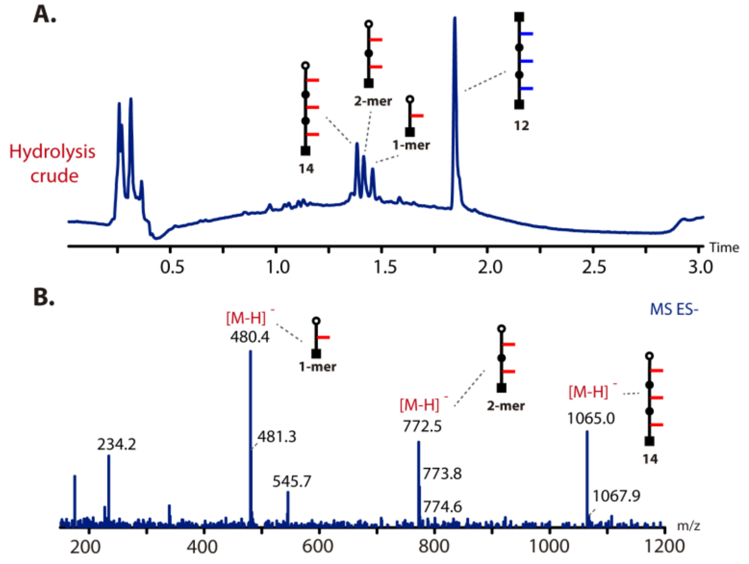

Figure S11. A. UPLC trace of the crude after hydrolysis of the mixture obtained when using [cap]=12.5 mM for the CuAAC reaction of **1**. *Conditions:* C18 column at 40 °C (254 nm) using water + 0.1% formic acid (A) and CH_3CN + 0.1% formic acid (B) with flow rate of 0.6 ml/min; Gradient of 0-2 minutes 5% - 100%B + 1 minute 100% B. **B.** Mass spectra for the peaks between 1.4 and 1.5 min corresponding to a mixture of **14**, linear 2-mer and 1-mer.

Synthesis and characterization of described compounds

Synthesis of *N*-(prop-2-yn-1-yl)-3-(triisopropylsilyl)prop-2-yn-1-amine hydrochloride (S4)

Monoprotected dipropragylamine derivative **S4** was prepared as described in Scheme S5. Protection of propragylamine with TIPS-Cl followed by reaction with di-*tert*-butyl dicarbonate afforded compound **S2** in excellent yield. The alkylation of **S2** with propargyl bromide and the treatment with HCl yielded monoprotected dipropargylamine hydrochloride **S4** in excellent yield.

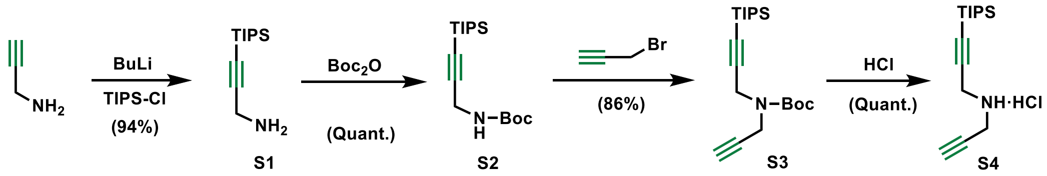

Scheme S5

3-(triisopropylsilyl)prop-2-yn-1-amine (S1)

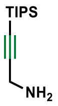

A solution of propargylamine (0.20 mL, 3.21 mmol) in THF (10 mL) was cooled to −78 °C while stirring in an argon atmosphere. *n*-Butyllithium (1.6 M in hexanes, 2.15 mL, 3.44 mmol) was added slowly, and the reaction was allowed to proceed for 15 min. The mixture was then warmed to 0 °C, and triisopropylsilyl chloride (0.80 mL, 3.75 mmol) was added. After stirring for 1. h at 0 °C, the reaction mixture was diluted with satd. aq. $NaHCO_3$ (5 mL) and stirred for 5 min. The solution was diluted with water (5 mL), and the product was extracted with diethyl ether (3x10). The combined organic phase was washed with brine (5 mL), dried with anhydrous $MgSO_4$, filtered, and the solvents evaporated. The obtained residue was purified by flash chromatography (EtOAc:Petroleum ether 1:2) to afford amine **S1** (0.621 g, 94%). Characterization data matches with previously reported data.[S3]

^{1}H NMR (400 MHz, $CDCl_3$): δ_H = 3.46 (s, 2H), 1.44 (bs, 2H), 1.06 (m, 21 H).

^{1}H-NMR (400 MHz, $CDCl_3$) compound S1.

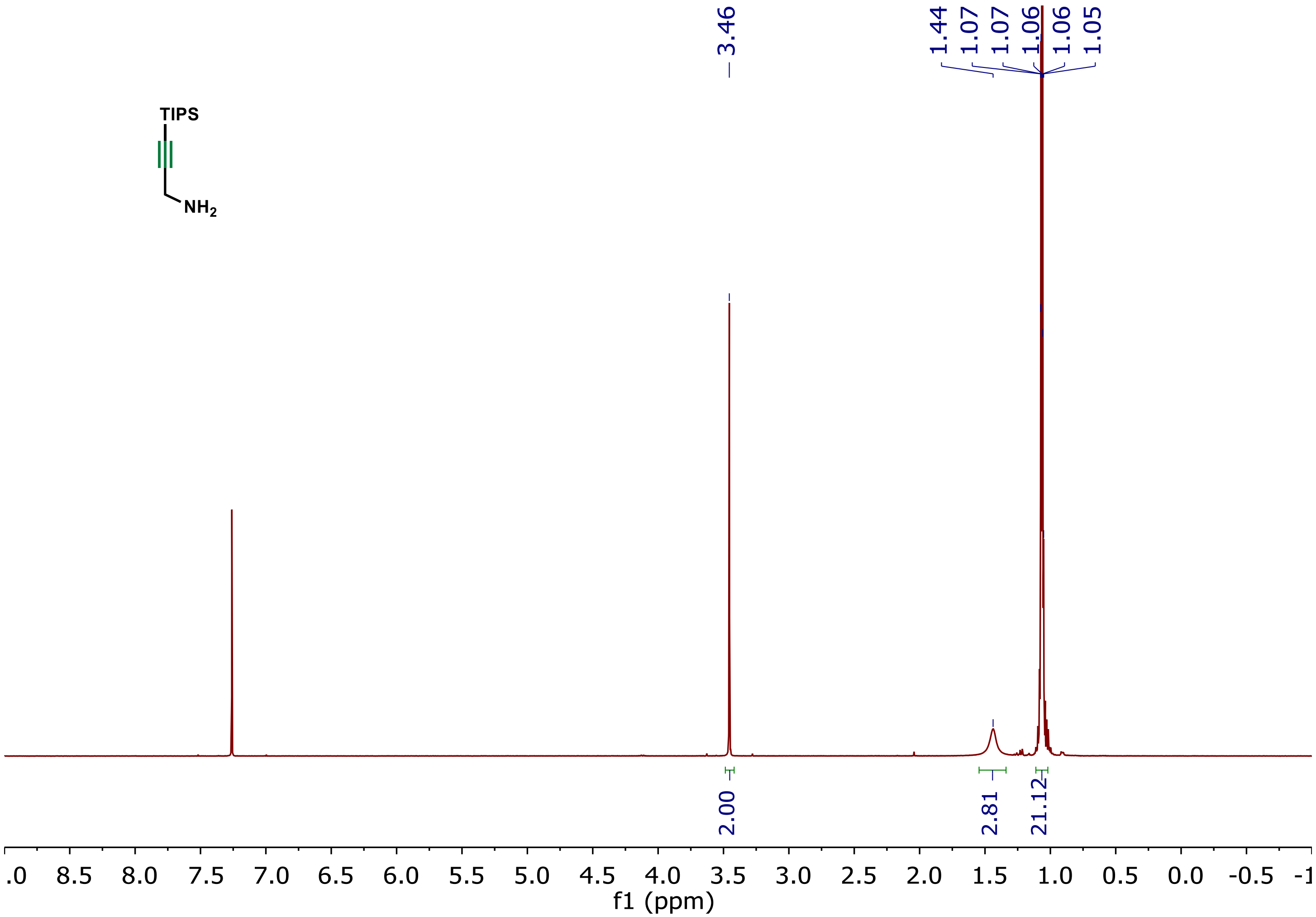

***tert*-Butyl (3-(triisopropylsilyl)prop-2-yn-1-yl)carbamate (S2).**

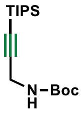

Boc_2O (0.769 g, 3.53 mmol) was added to a solution of **S1** (0.621 g, 2.94 mmol) in dry CH_2Cl_2 (10 mL). TEA (0.61 mL, 4.41 mmol) was added and the reaction was allowed to proceed for 1 h. After completion, the reaction was quenched with satd. aq. NH_4Cl and extracted with CH_2Cl_2 (3x5 mL). The combined organic phase was washed with satd. aq. $NaHCO_3$ (5 mL), H_2O and brine, and dried with anhydrous $MgSO_4$. The obtained white solid, **S2**, (0.972 g, quantitative) was used in the following step without further characterization.

Melting point: 66-68 ^oC.

^1H NMR (400 MHz, $CDCl_3$): δ_H = 4.63 (bs, 1H), 3.95 (d, 2H, *J*= 5.5 Hz), 1.45 (s, 9H), 1.05 (m, 21H).

^{13}C NMR (100.6 MHz, $CDCl_3$): δ_C = 155.4, 103.7, 80.1, 77.4, 31.8, 28.5, 18.7, 11.3.

HRMS (ES+): calcd for $C_{17}H_{34}NO_2Si$ 312.2359[M+H]$^+$, found 312.2353 [M+H]$^+$.

FT-IR (ATR): ν_{max} 3340, 2942, 2893, 2866, 1700, 1462, 1247, 1170, 1016, 1001, 677, 664 cm^{-1}.

^{1}H-NMR (400 MHz, $CDCl_3$) compound S2.

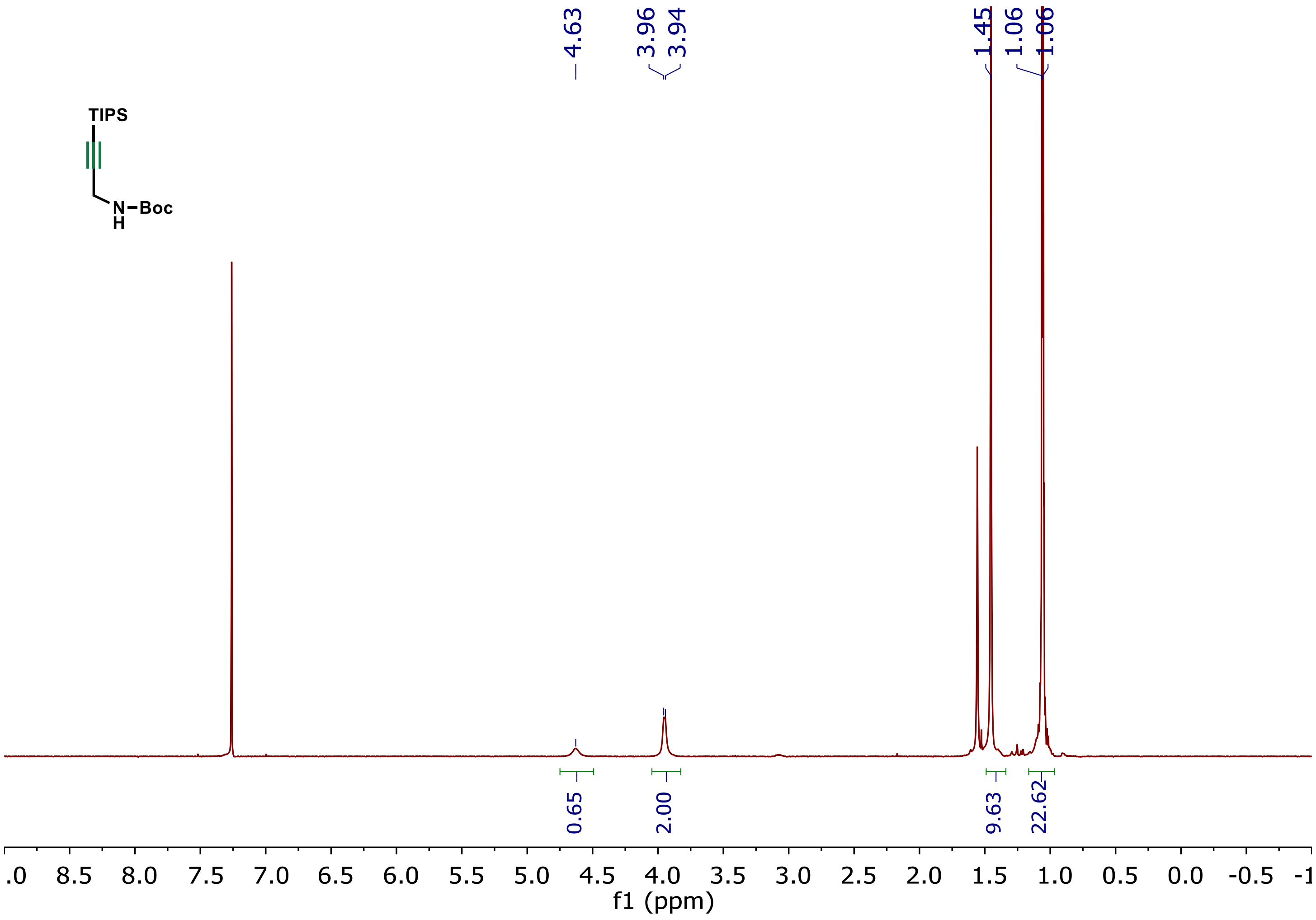

^{13}C-NMR (100.6 MHz, $CDCl_3$) compound S2.

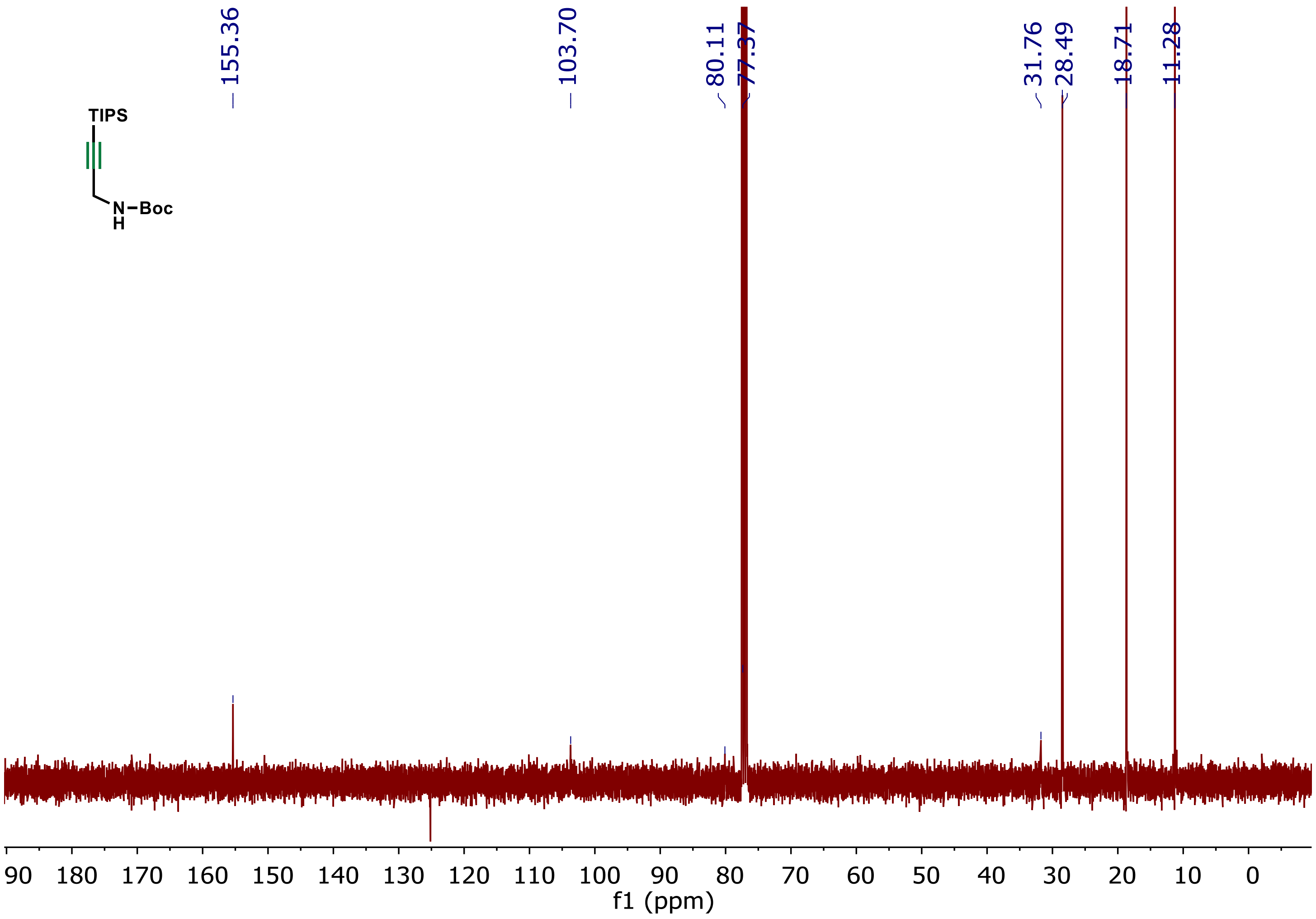

tert-Butyl prop-2-yn-1-yl(3-(triisopropylsilyl)prop-2-yn-1-yl)carbamate (S3).

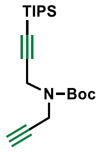

Compound **S2** (11.67 g, 37.46 mmol) was dissolved in dry THF (20 mL) and added to a suspension of NaH (60% dispersion in mineral oil, 2.25g, 56.19 mmol) in THF (100 mL) under inert atmosphere. The reaction was allowed to proceed for 45 min. Porpargyl bromide (80% in toluene, 6.05 mL, 56.19 mmol) was added dropwise and the reaction was vigorously stirred overnight. Satd. aq. NH_4Cl (20 mL) was carefully added at 0 °C and the reaction was extracted with EtOAc (3x100 mL). The combined organic phase was washed with brine (5 mL), dried with anhydrous $MgSO_4$, filtered, and the solvents evaporated. The obtained residue was purified by flash chromatography (EtOAc:Petroleum ether 1:9) to afford **S3** (11.2 g, 86%) as a yellow oil.

^{1}H NMR (400 MHz, CDCl$_3$): δ_H = 4.18 (bs, 4H), 2.20 (t, 1H, *J*= 2.5 Hz), 1.48 (s, 9H), 1.06 (m, 21H).

^{13}C NMR (100.6 MHz, CDCl$_3$): δ_C = 154.5, 102.5, 81.1, 79.2, 77.4, 72.0, 36.6, 35.2, 28.4, 18.7, 11.3.

HRMS (ES+): calcd for $C_{20}H_{36}NO_2Si$ 350.2515 $[M+H]^+$, 350.2513 found $[M+H]^+$.

FT-IR (ATR): ν_{max} 2943, 2866, 1703, 1367, 1243, 1162, 1007, 883, 677 and 663 cm^{-1}.

^{1}H-NMR (400 MHz, $CDCl_3$) compound S3.

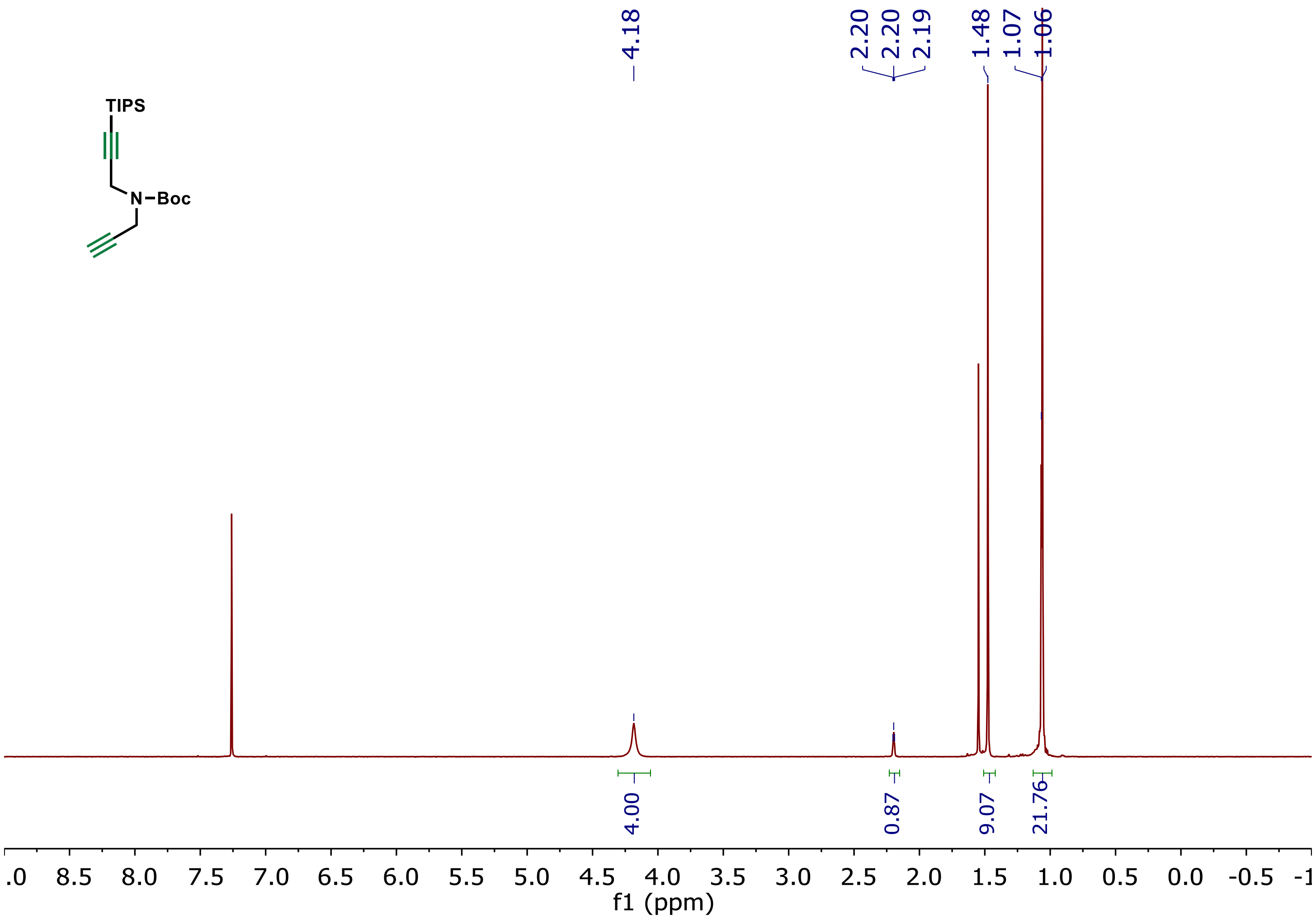

^{13}C-NMR (100.6 MHz, $CDCl_3$) compound S3.

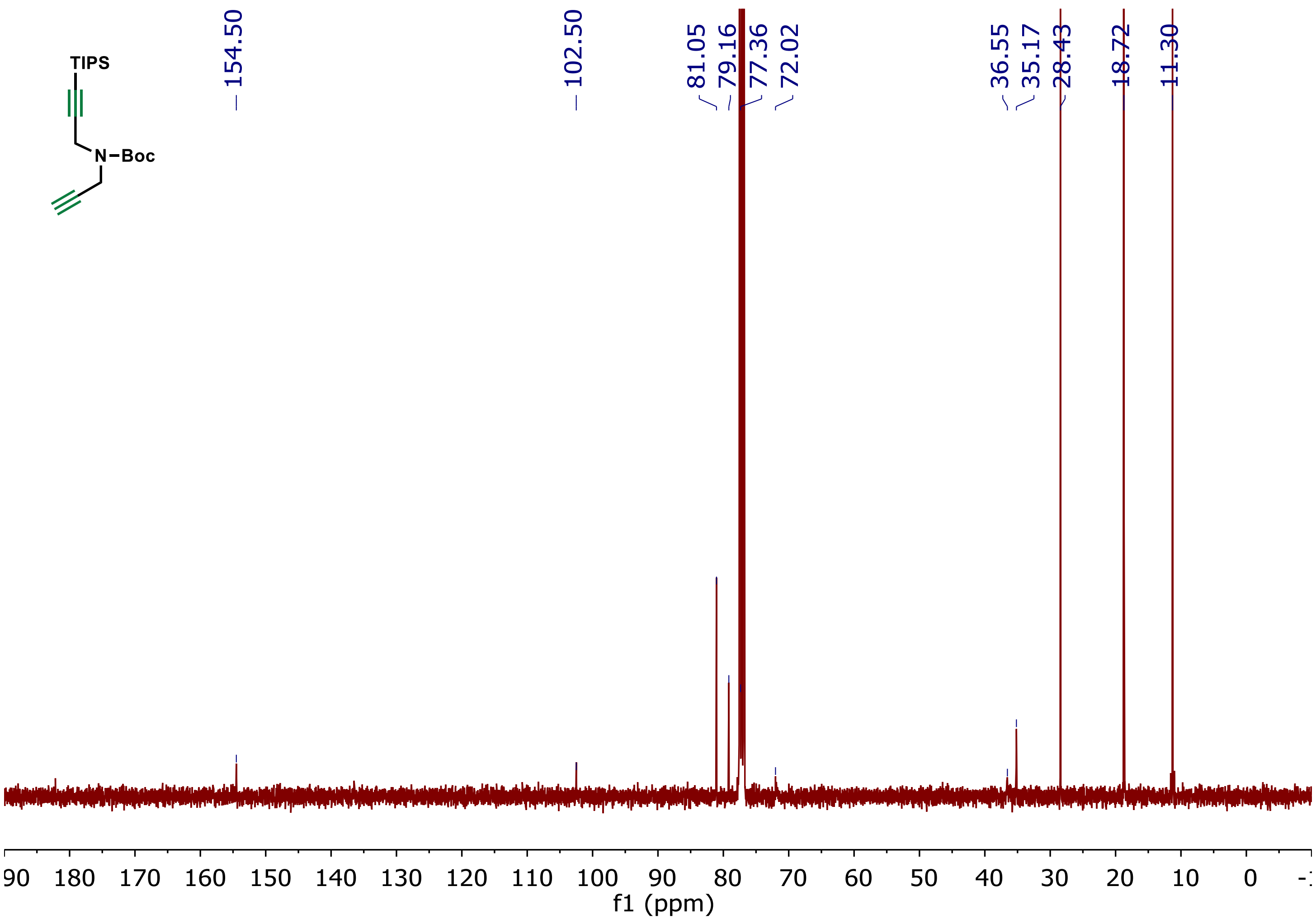

***N*-(prop-2-yn-1-yl)-3-(triisopropylsilyl)prop-2-yn-1-amine hydrochloride (S4).**

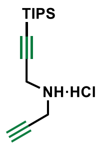

Compound **S3** (11.2 g, 32.04 mmol) was dissolved in MeOH (100 mL) and aq. HCl (37%, 40 mL) was added dropwise. The reaction was stirred overnight at room temperature. The solvents were evaporated, and co-evaporated with toluene to remove residual water. The crude was dried at high vacuum. Compound **S4** (9.05 g, 99%) was obtained as a brown solid and used in the following reaction without further purification.

Melting point: 95-97 ^{o}C.

^{1}H NMR (400 MHz, $CDCl_3$): δ_H = 4.18 (bs, 4H), 2.20 (t, 1H, *J*= 2.5 Hz), 1.48 (s, 9H), 1.06 (m, 21H).

^{13}C NMR (100.6 MHz, $CDCl_3$): δ_C = 93.4, 94.6, 78.3, 72.5, 34.3, 35.5, 18.7, 11.2.

HRMS (ES+): calcd for $C_{15}H_{28}NSi$ 250.1991 $[M\text{-}HCl+H]^+$, found 250.1992 $[M\text{-}HCl+H]^+$.

FT-IR (ATR): ν_{max} 2942, 2893, 2865, 1462, 1440, 1050, 882, 676, 662 and 633 cm^{-1}.

^{1}H-NMR (400 MHz, $CDCl_3$) compound S4.

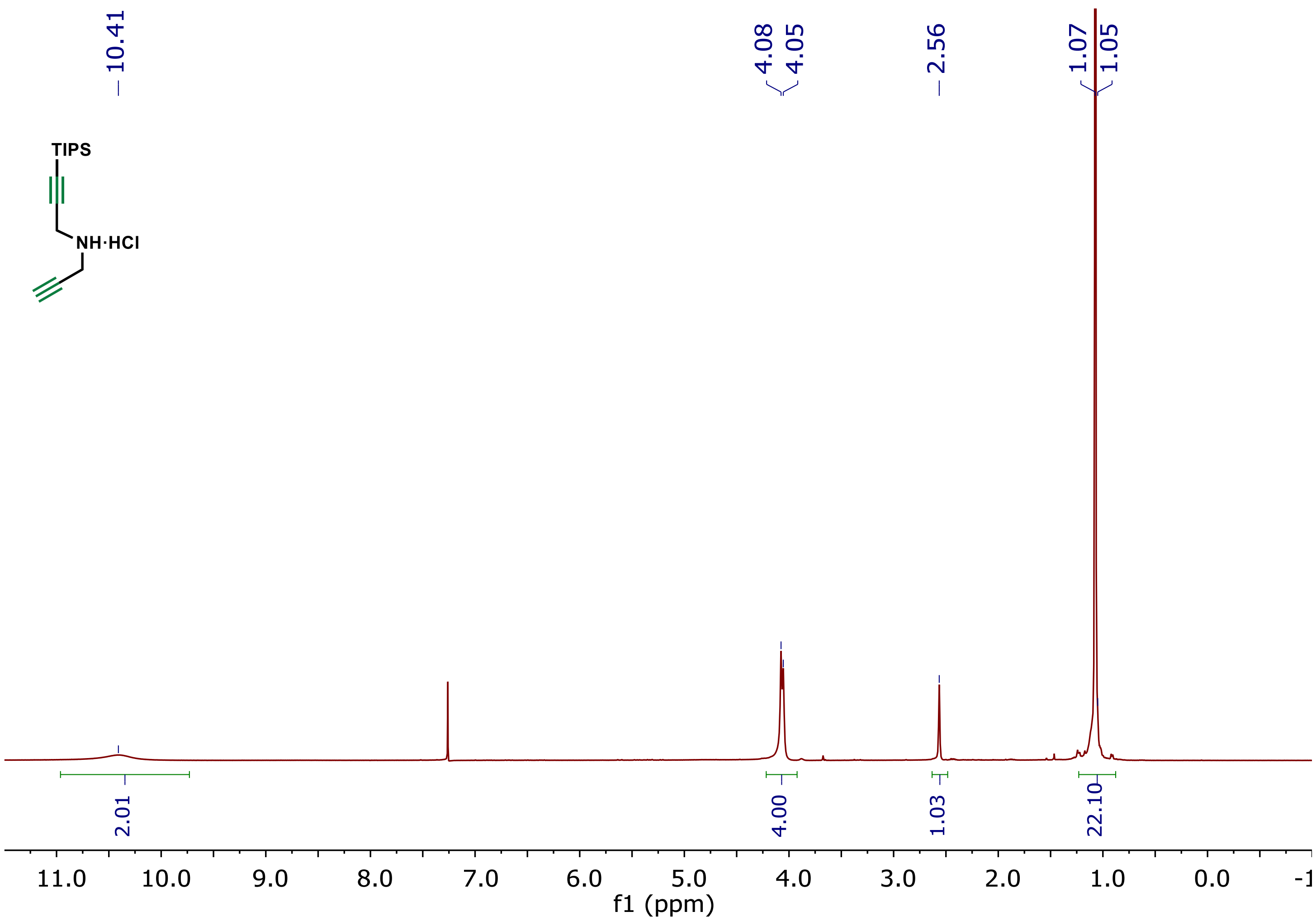

^{13}C-NMR (100.6 MHz, $CDCl_3$) compound S4.

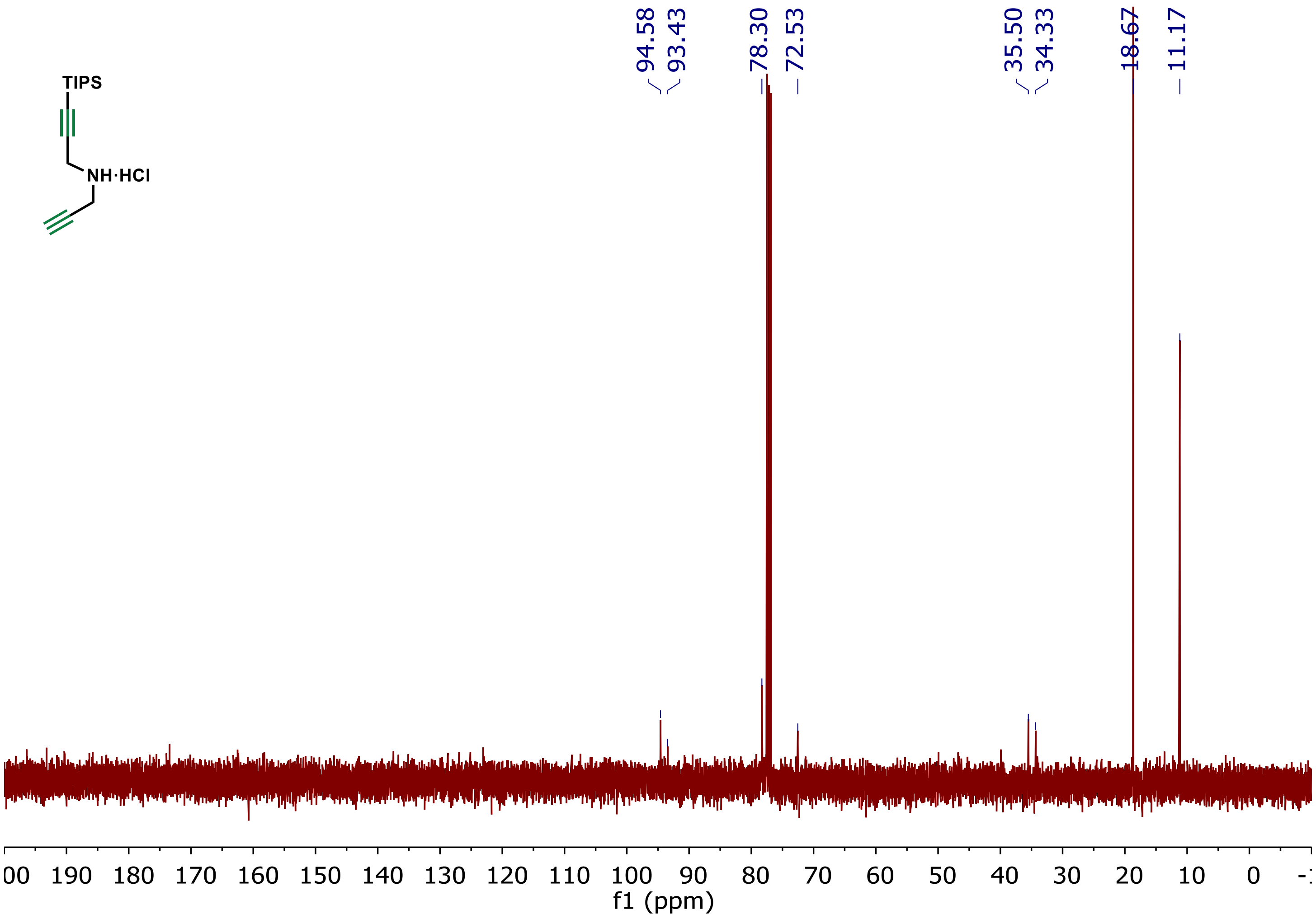

Synthesis of pre-ZIP 1

Scheme S6 shows the synthetic route towards pre-ZIP intermediate **1**.

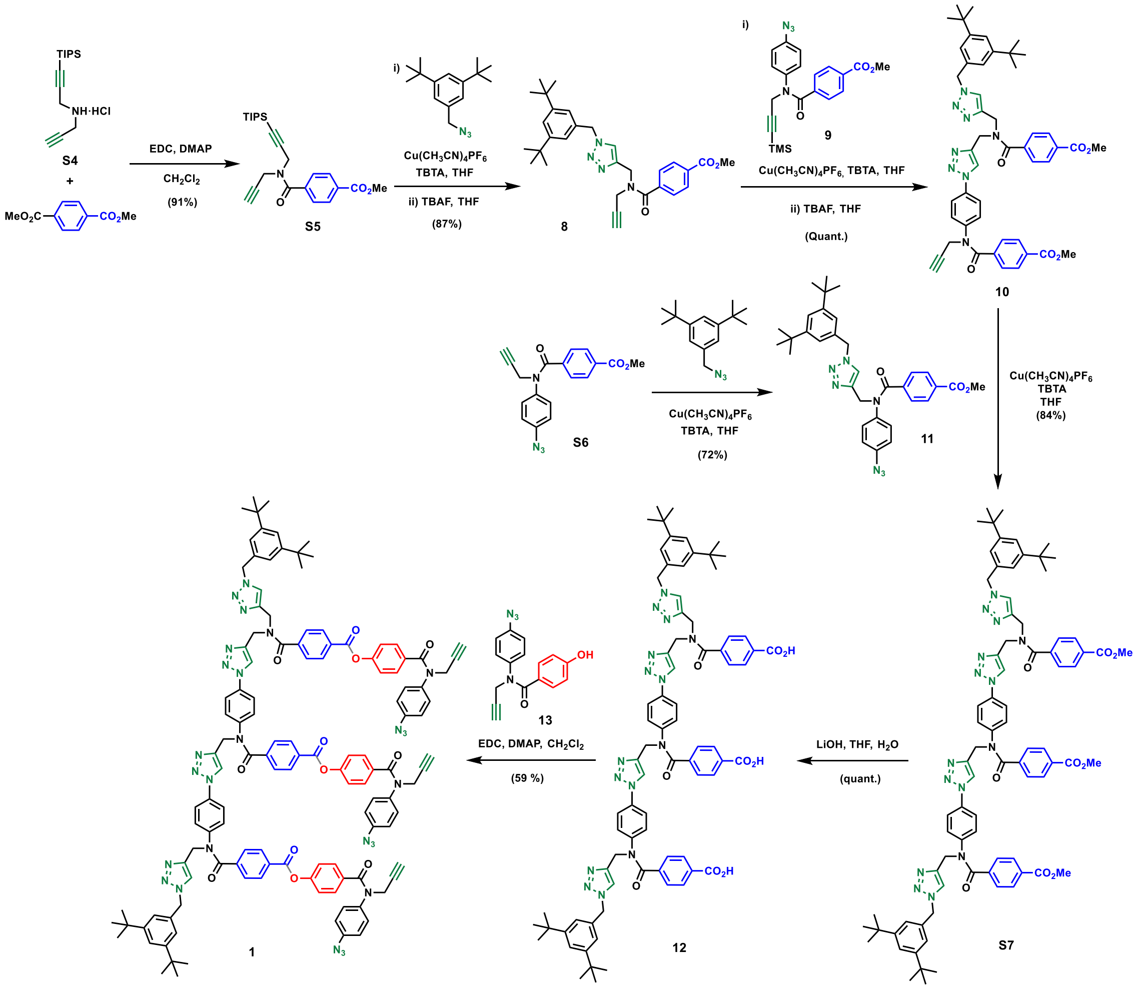

Scheme S6

Methyl 4-(prop-2-yn-1-yl(3-(triisopropylsilyl)prop-2-yn-1-yl)carbamoyl)benzoate (S5).

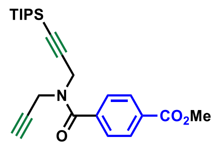

Mono-methyl terephthalate (0.034 g, 0.19 mmol), EDC (0.046 g, 0.21 mmol) and DMAP (0.002 g, 0.02 mmol) were dissolved in dry CH_2Cl_2 (3 ml). Compound **S4** (0.060 g, 0.21 mmol) and TEA (0.029 mL, 0.21 mmol) were added under N_2 atmosphere and the solution was left stirring at room temperature for 1h. The crude was diluted with EtOAc (50 mL) and washed with 5% aq. soln. HCl (3x), H_2O (1x) and brine. The organic phase was dried with $MgSO_4$ and concentrated *in vacuo* to yield **S5** as an orange oil (0.072 g, 91%).

^{1}H NMR (400 MHz, $CDCl_3$): δ_H = 8.09 (d, 2H, *J* = 8.0 Hz), 7.62 (bs, 2H), 4.48 and 4.14 (bs, 4H, rotamers), 3.93 (s, 3H), 2.30 (s, 1H), 1.07 (s, 21H).

^{13}C NMR (100.6 MHz, $CDCl_3$): δ_C = 169.9, 166.4, 139.1, 131.9, 129.9, 127.2, 101.2, 87.2, 78.0, 72.6, 52.5, 39.4, 33.9, 18.7, 11.2.

HRMS (ES+): calcd for C24H34NO$_3$Si 412.2308 $[M+H]^+$, found 412.2311 $[M+H]^+$.

FT-IR (ATR): ν_{max} 2943, 2865, 1727, 1650, 1434, 1410, 1276, 1247, 1107, 677 and 662 cm^{-1}.

^{1}H-NMR (400 MHz, $CDCl_3$) compound S5.

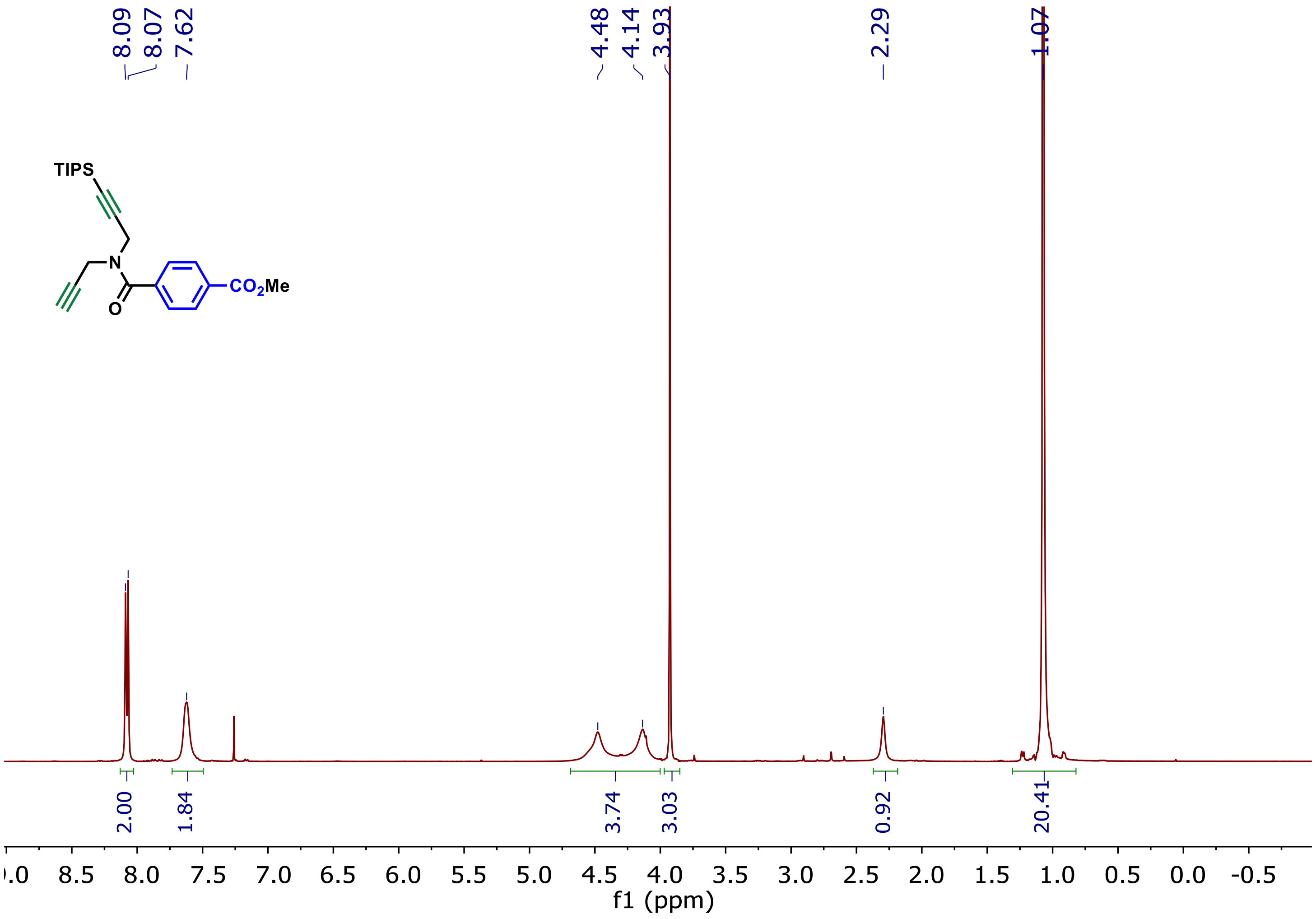

^{13}C-NMR (100.6 MHz, $CDCl_3$) compound S5.

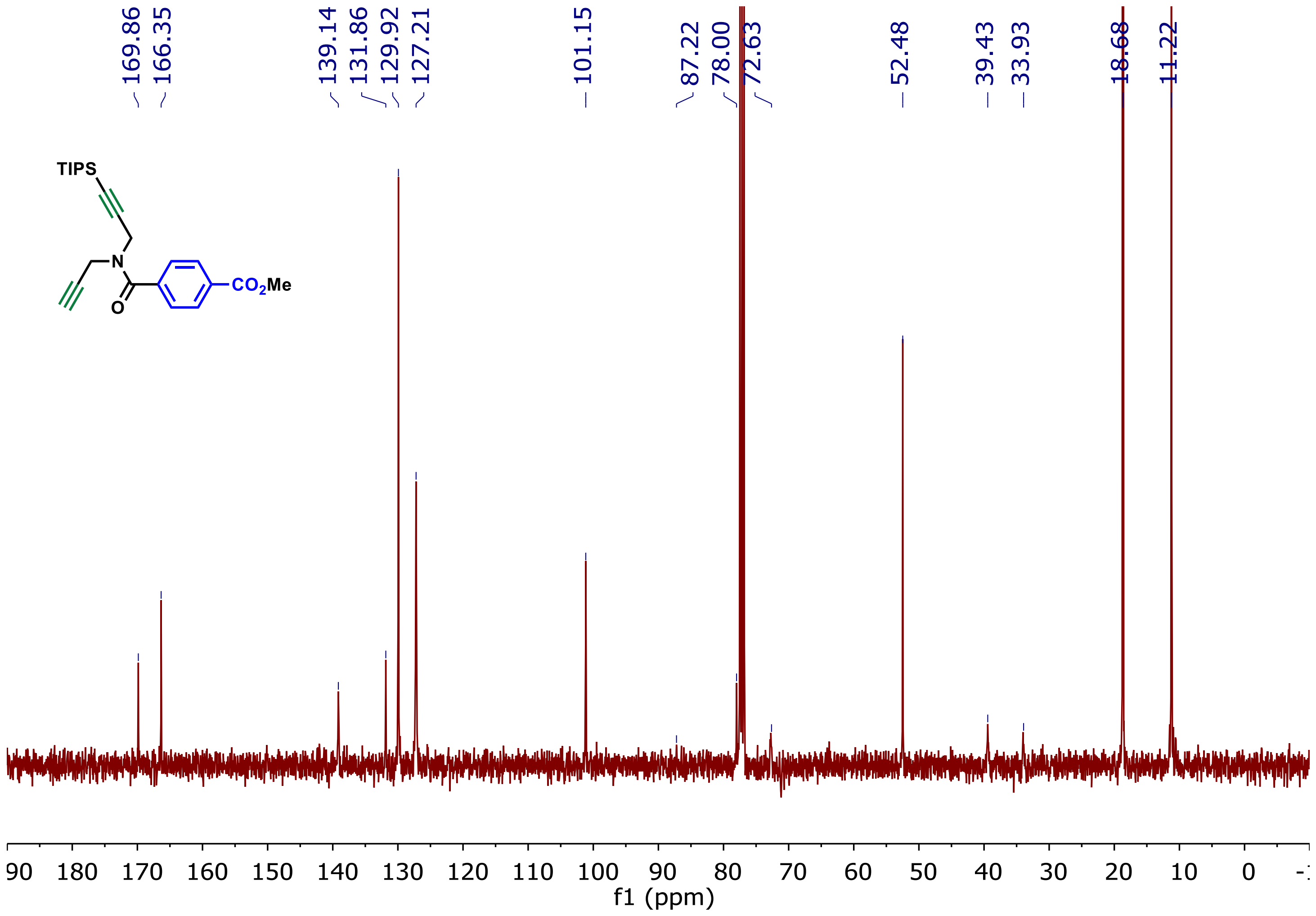

Compound 8.

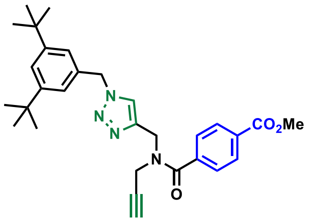

S5 (0.250 g, 0.61 mmol), $Cu(CH_3CN)_4PF_6$ (0.023 g, 0.06 mmol) and TBTA (0.032 g, 0.06 mmol) were mixed in a round-bottom flask and, under N_2, THF (5 mL) was added. A solution of 1-(azidomethyl)-3,5-di-*tert*-butylbenzene[S4] (0.0179 g, 0.73 mmol) in THF (2 mL) was added and the reaction was stirred overnight at room temperature. TBAF (1M in THF, 0.728 mL, 0.728 mmol) was added and the reaction stirred at room temperature for 10 min. The solvent was evaporated to dryness and the crude was dissolved in EtOAc and washed with 0.02 M EDTA soln. (1x), 0.1 N HCl soln, (2x), H_2O (2x) and brine. The organic phase was dried over $MgSO_4$ and the solvent removed under vacuum. The crude was purified by flash column chromatography on silica gel (gradient from 10% to 100%of EtOAc in Pet. Ether) to afford **8** (0.263 g, 87%) as a clear syrup.

^{1}H NMR (400 MHz, CDCl$_3$): δ_H = 8.07 (d, 2H, *J* = 8.0 Hz), 7.64 (s partially overlapped, 1H), 7.57 (d, 2H, *J* = 8.0 Hz), 7.42 (s, 1H), 7.10 (s, 2H), 5.50 (s, 2H), 4.85, 4.63, 4.35 and 4.06 (bs, 4H, rotamers), 3.93 (s, 3H), 2.32 and 2.22 (bs, 1H, rotamers), 1.30 (s, 18H).

^{13}C NMR (125.7 MHz, CDCl$_3$): δ_C = 170.3, 166.4, 152.0, 143.7, 139.3, 133.8, 131.9, 130.0, 127.5, 127.3, 123.6, 123.0, 122.5, 78.5, 73.5, 55.0, 52.5, 40.1 and 39.3 (rotamers), 35.1, 31.5.

HRMS (ES+): calcd for $C_{30}H_{37}N_4O_3$ 501.2866 $[M+H]^+$, found 501.2856 $[M+H]^+$.

FT-IR (ATR): ν_{max} 2955, 1725, 1639, 1456, 1277, 1249, 1110 and 754 cm^{-1}.

^{1}H-NMR (400 MHz, $CDCl_3$) Compound 8.

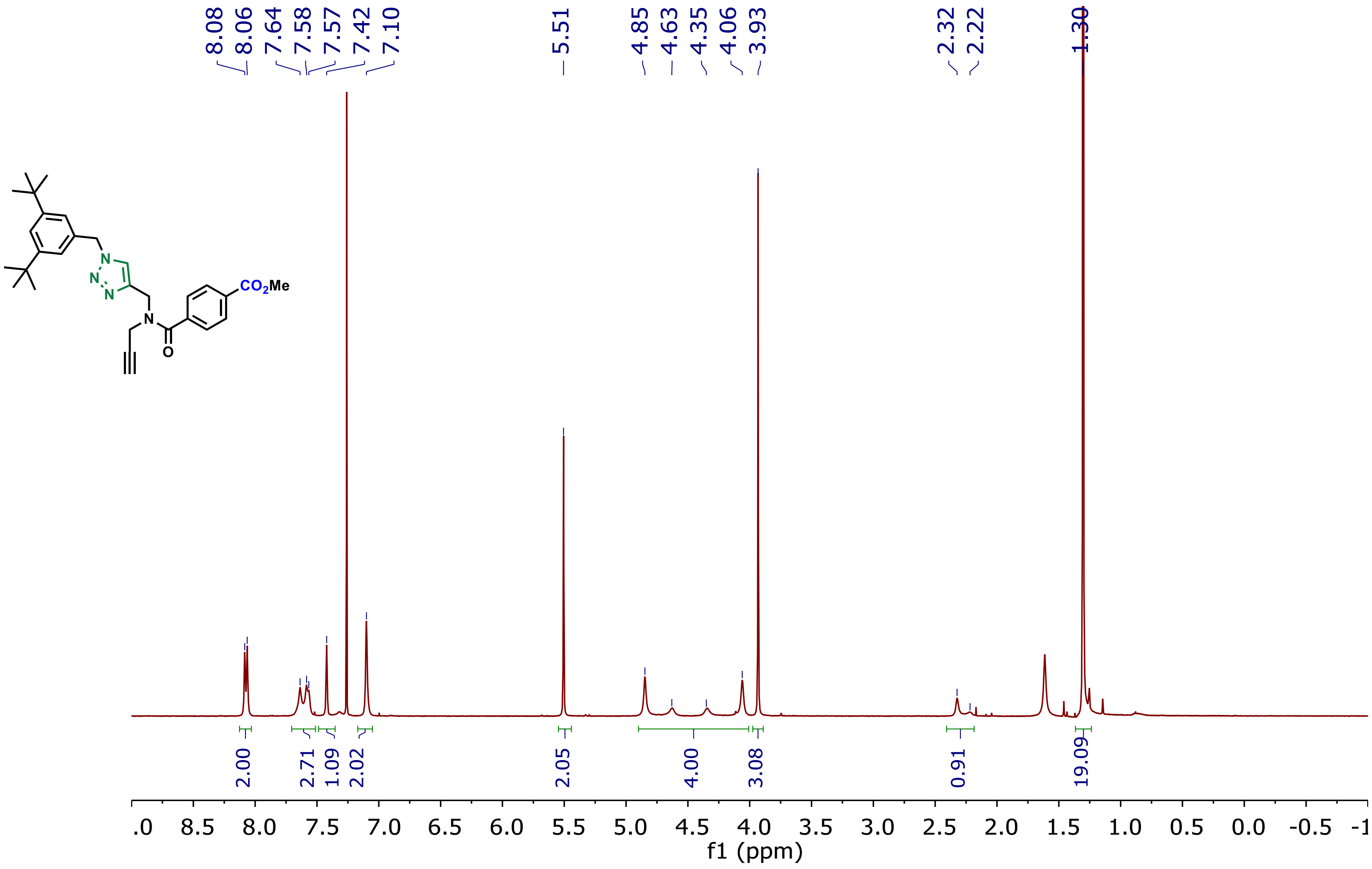

^{13}C-NMR (125.7 MHz, $CDCl_3$) Compound 8.

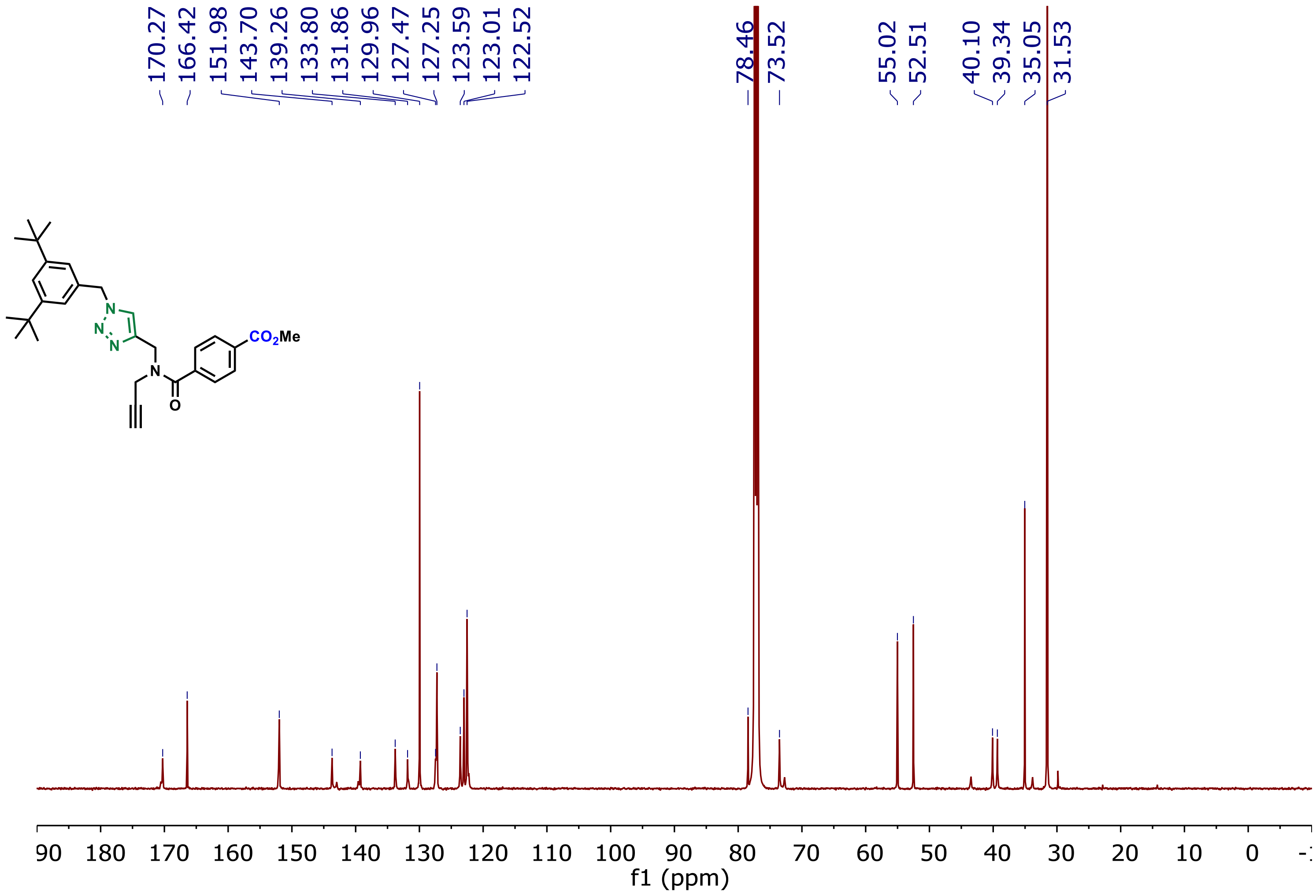

Compound 10

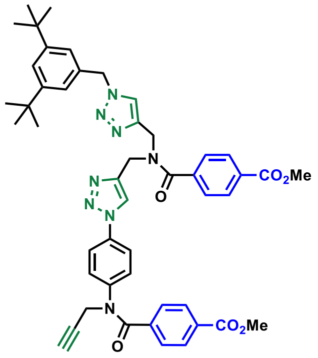

9[S2] (0.034 g, 0.08 mmol) and **8** (0.049 g, 0.10 mmol) were dissolved in anhydrous THF (10 mL) under N_2 atmosphere. Then, TBTA (5 mg, 0.01 mmol) and $Cu(CH_3CN)_4PF_6$ (3.6 mg, 0.01 mmol) were dissolved in THF (5 mL) and this solution added to the reaction mixture. After 16 h of stirring at room temperature, TBAF solution (1M in THF, 0.098 mL, 0.10 mmol) was added, the reaction stirred for 10 minutes at room temperature and quenched with 5% soln. HCl. The reaction was extracted with EtOAc (3x) followed by washing with H_2O and brine. The organic layer was dried over anhydrous $MgSO_4$ and concentrate under vacuum. The crude was purified by flash column chromatography on silica gel (gradient from 10% to 75% of EtOAc in Pet. Ether) to afford **10** (0.070 g, quantitative) as a foam.

^{1}H NMR (400 MHz, $CDCl_3$): δ_H = 8.14 (s, 1H), 8.06 (t, 2H, *J* = 8.5 Hz), 7.89 (d, 2H, *J* = 8.0 Hz), 7.73 (d, 2H, *J* = 8.5 Hz), 7.68 (m, 2H), 7.66 and 7.51 (s, 1H, rotamers), 7.44 (s, 1H), 7.42 (s, 2H), 7.31 (t, 2H, *J* = 7.5 Hz), 7.10 (s, 2H), 5.51 and 5.50 (s, 2H, rotamers), 4.73 and 4.70 (s, 2H, rotamers), 4.62 and 4.56 (s, 2H, rotamers), 3.92 (s, 3H), 3.86 (s, 3H), 2.31 (s, 1H), 1.30 (s, 18H).

^{13}C NMR (100.6 MHz, $CDCl_3$): δ_C = 169.3, 166.5, 166.2, 152.0, 152.0, 139.6, 139.1, 133.7, 131.7, 131.7, 129.9, 129.5, 129.2, 128.8, 127. 8, 124.0, 123.1, 122. 9, 122.5, 122.0, 121.5, 121.3, 78.4, 73.3, 55.1, 52.5, 47.3, 44.3, 39.8, 39.7, 35.0, 31.5.

HRMS (ES+): calcd for $C_{48}H_{51}N_8O_6$ 835.3932 $[M+H]^+$, found 835.3927 $[M+H]^+$.

FT-IR (ATR): ν_{max} 2961, 1721, 1643, 1519, 1277, 1107, 1046, 1020 and 750 cm^{-1}.

^{1}H-NMR (400 MHz, $CDCl_3$) Compound 10.

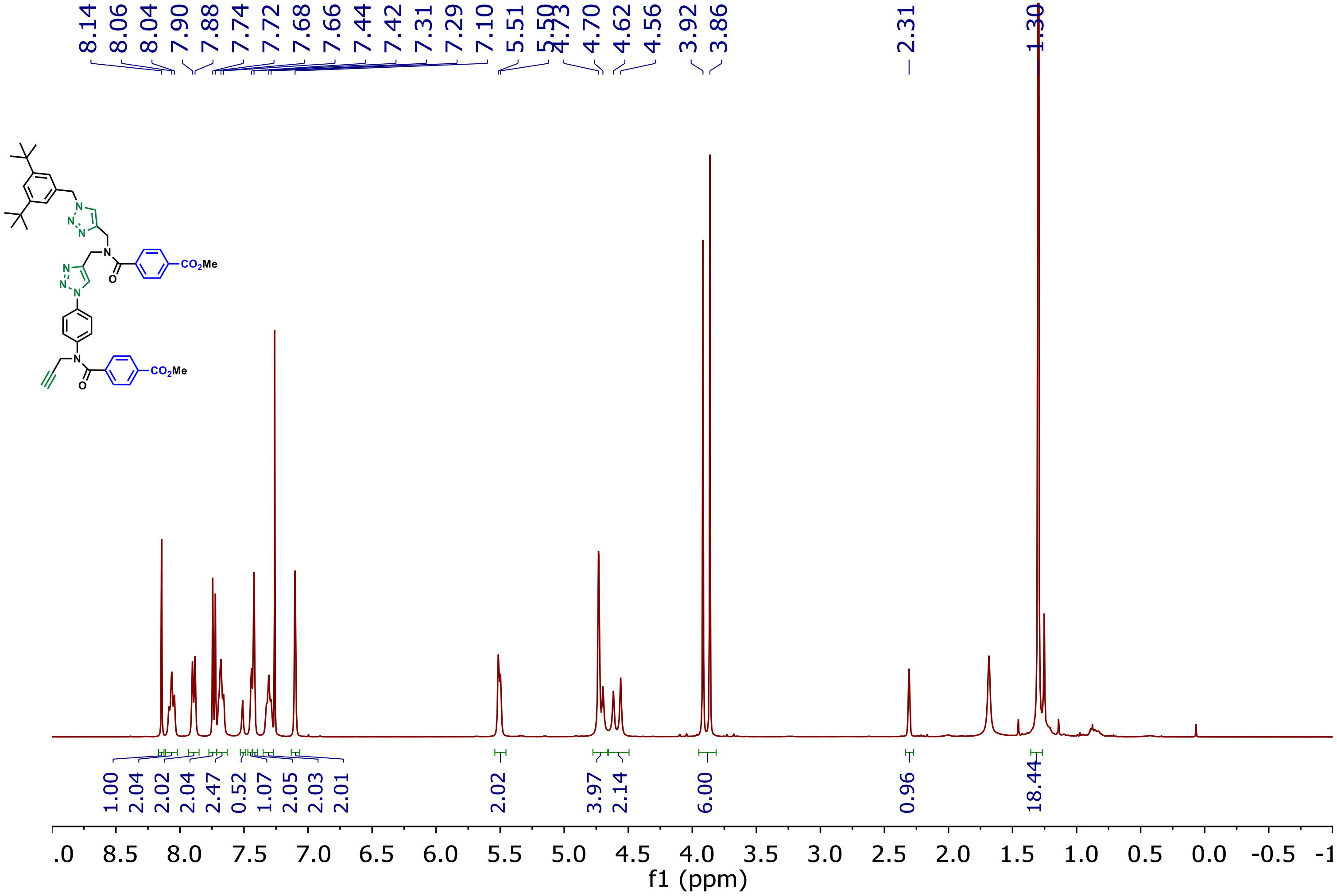

^{13}C-NMR (100.6 MHz, $CDCl_3$) Compound 10.

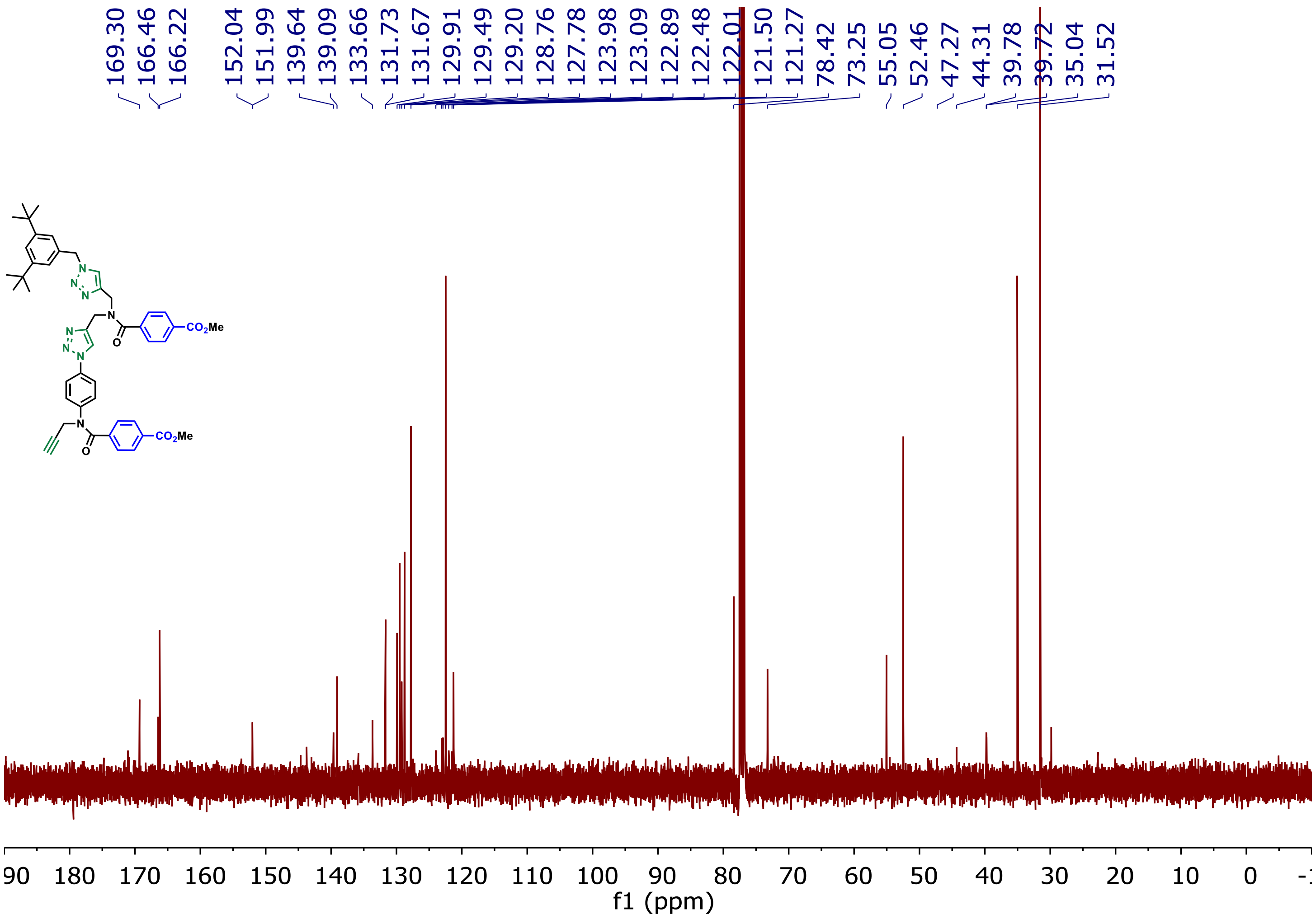

Compound 11.

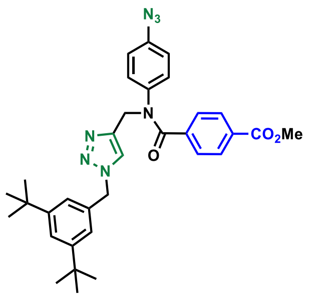

Compound **S6**[S2] (0.030 g, 0.09 mmol) and 1-(azidomethyl)-3,5-di-*tert*-butylbenzene (0.220 g, 0.90 mmol) were mixed in a round-bottom flask and, under N_2, THF (100 mL) was added. $Cu(CH_3CN)_4PF_6$ (0.003 g, 0.09 mmol) and TBTA (0.005 g, 0.09 mmol) were added and the reaction was stirred overnight at room temperature. Once the reaction was completed, the solvent was evaporated and the crude was purified by flash column chromatography on silica gel (gradient from 0% to 60% of EtOAc in Pet. Ether) to afford **11** (0.037 g, 72%) as a foam.

^{1}H NMR (400 MHz, CDCl$_3$): δ_H = 7.83 (d, 2H, *J* = 8.5 Hz), 7.67 (s, 1H), 7.40 (t, 1H, *J* = 2.0 Hz), 7.29 (d, 2H, *J* = 8.5 Hz), 7.07 (m, 4H), 6.82 (d, 2H, *J* = 8.5 Hz), 5.50 (s, 2H), 5.08 (s, 2H), 3.87 (s, 3H), 1.28 (s, 18H).

^{13}C NMR (100.6 MHz, CDCl$_3$): δ_C = 169.4, 166.4, 151.9, 144.0, 140.1, 139.8, 139.0, 133.9, 131.3, 129.3, 129.0, 128.7, 123.9, 122.9, 122.3, 119.9, 54.9, 52.4, 46.3, 35.0, 31.5.

HRMS (ES+): calcd for $C_{33}H_{38}N_7O_3$ 580.3036 $[M+H]^+$, found 580.3023 $[M+H]^+$.

FT-IR (ATR): ν_{max} 2956, 2925, 2867, 2125, 2095, 1725, 1646, 1506, 1277 and 1108 cm^{-1}.

^{1}H NMR (400 MHz, $CDCl_3$) compound 11.

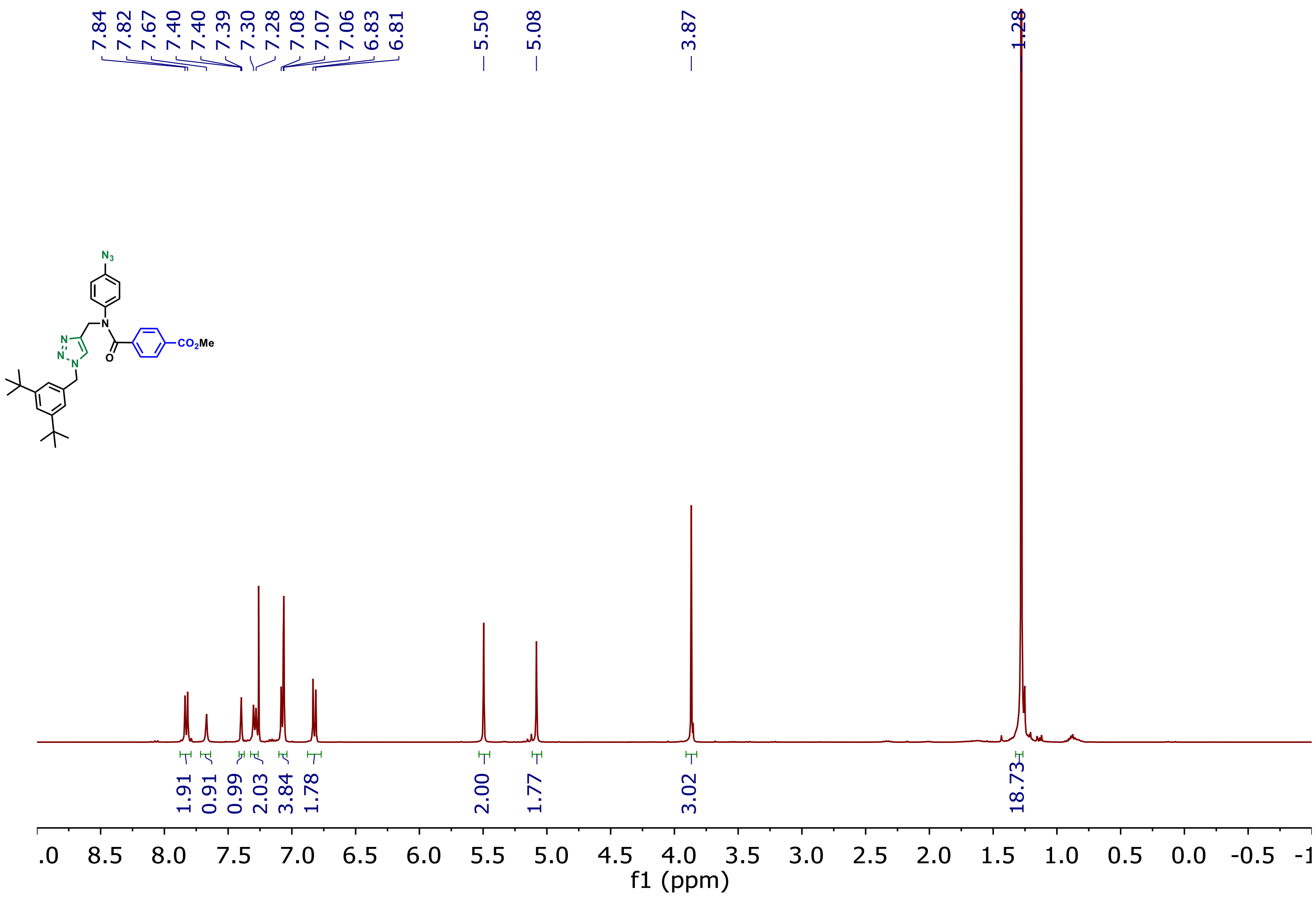

^{13}C NMR (100.6 MHz, $CDCl_3$) compound 11.

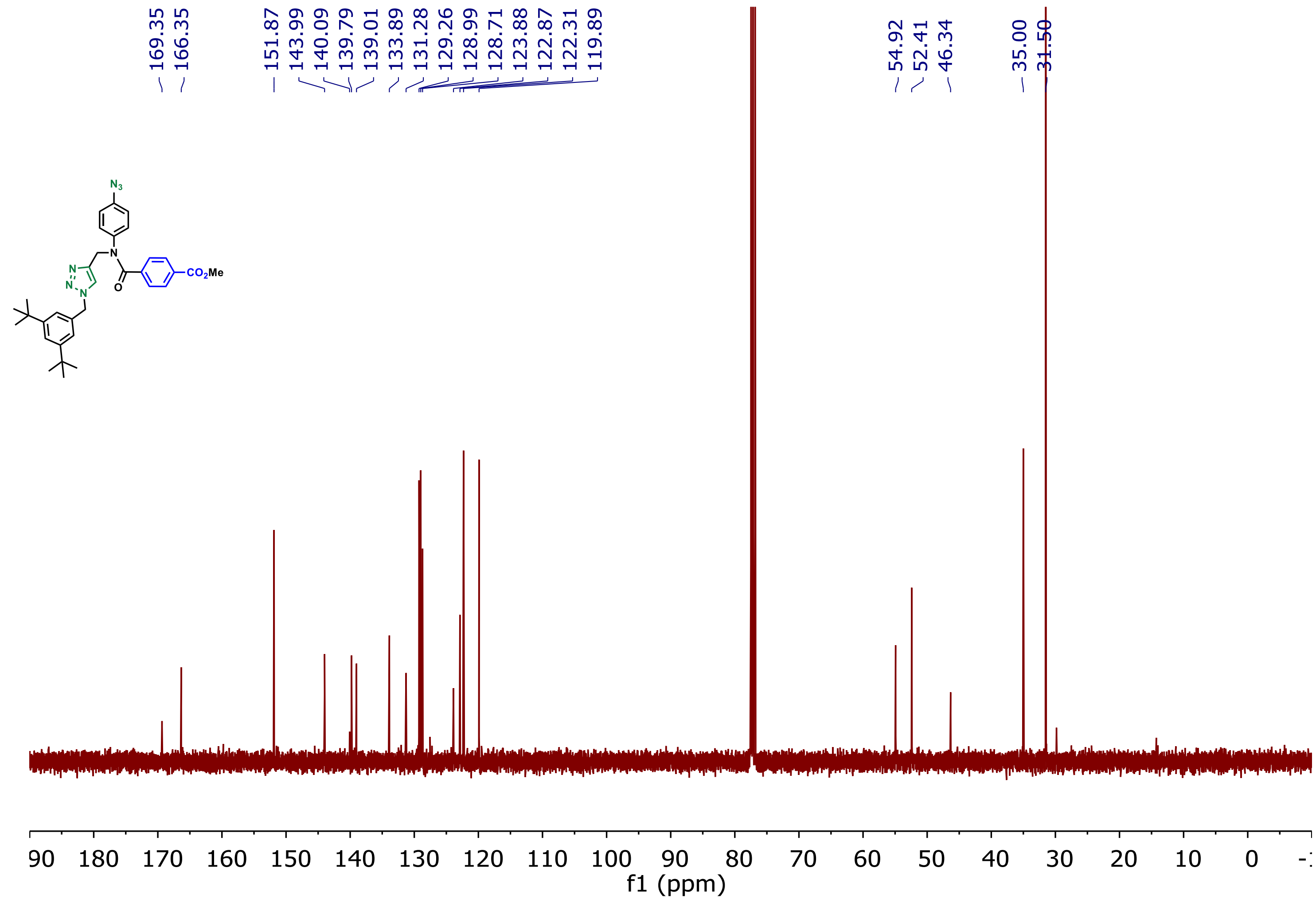

Compound S7.

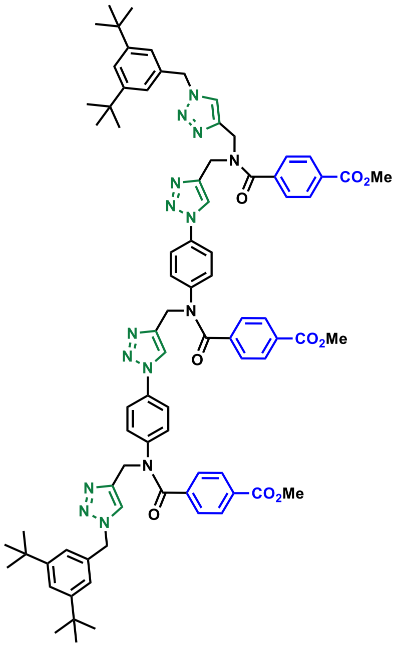

10 (0.040 g, 0.048 mmol), **11** (0.037 g, 0.064 mmol) were mixed in a round-bottom flask and, under N_2, THF (2 mL) was added. A solution of $Cu(CH_3CN)_4PF_6$ (0.002 g, 0.005 mmol) and TBTA (0.003 g, 0.005 mmol) in dry THF (0.5 mL) was added and the reaction was stirred overnight at room temperature. Then, the solvent was evaporated to dryness and the crude was purified by flash column chromatography on silica gel (gradient from 20% to 95% of EtOAc in Pet. Ether) to afford **S7** (0.057 g, 84%) as a light-yellow foam.

^{1}H NMR (400 MHz, $CDCl_3$): δ_H = 8.17 (s, 1H), 8.12 (s, 1H), 8.05 (m, 2H), 7.84 (m, 4H), 7.73 (d, 2H, *J* = 8.0 Hz), 7.70 (s, 1H), 7.68 and 7.52 (s, 1H, rotamers), 7.62 (m, 4H), 7.34 (m, 10H), 7.10 (s, 2H), 7.06 (s, 2H), 5.51 (s, 2H), 5.50 (s, 2H), 5.21 (s, 2H), 5.13 (s, 2H), 4.72 and 4.69 (s, 2H, rotamers), 4.61 and 4.55 (s, 2H, rotamers), 3.90 (s, 3H), 3.84 (s, 6H), 1.29 (s, 18H), 1.27 (s, 18H).

^{13}C NMR (100.6 MHz, $CDCl_3$): δ_C = 169.4, 169.3, 166.4, 166.2, 166.1, 151.8, 144.3, 143.7, 139.4, 139.2, 135.3, 133.8, 131.7, 131.6, 131.5, 129.8, 129.4, 129.4, 128.8, 128.8, 128.7, 127.7, 125.1, 123.9, 122.9, 122.4, 122.3, 122.0, 121.1, 55.0 and 54.9 (rotamers), 52.4 (OCH_3), 47.1, 46.3, 46.3, 45.6, 44.2, 43.9 and 39.4 (rotamers), 35.0, 34.9, 31.5, 31.5.

HRMS (ES+): calcd for $C_{81}H_{88}N_{15}O_9$ 1415.6923 $[M+H]^+$, found 1415.5874 $[M+H]^+$.

FT-IR (ATR): ν_{max} 2955, 1721, 1643, 1518, 1435, 1109, 1047, 748 and 710 cm^{-1}.

^{1}H-NMR (400 MHz, $CDCl_3$) Compound S7.

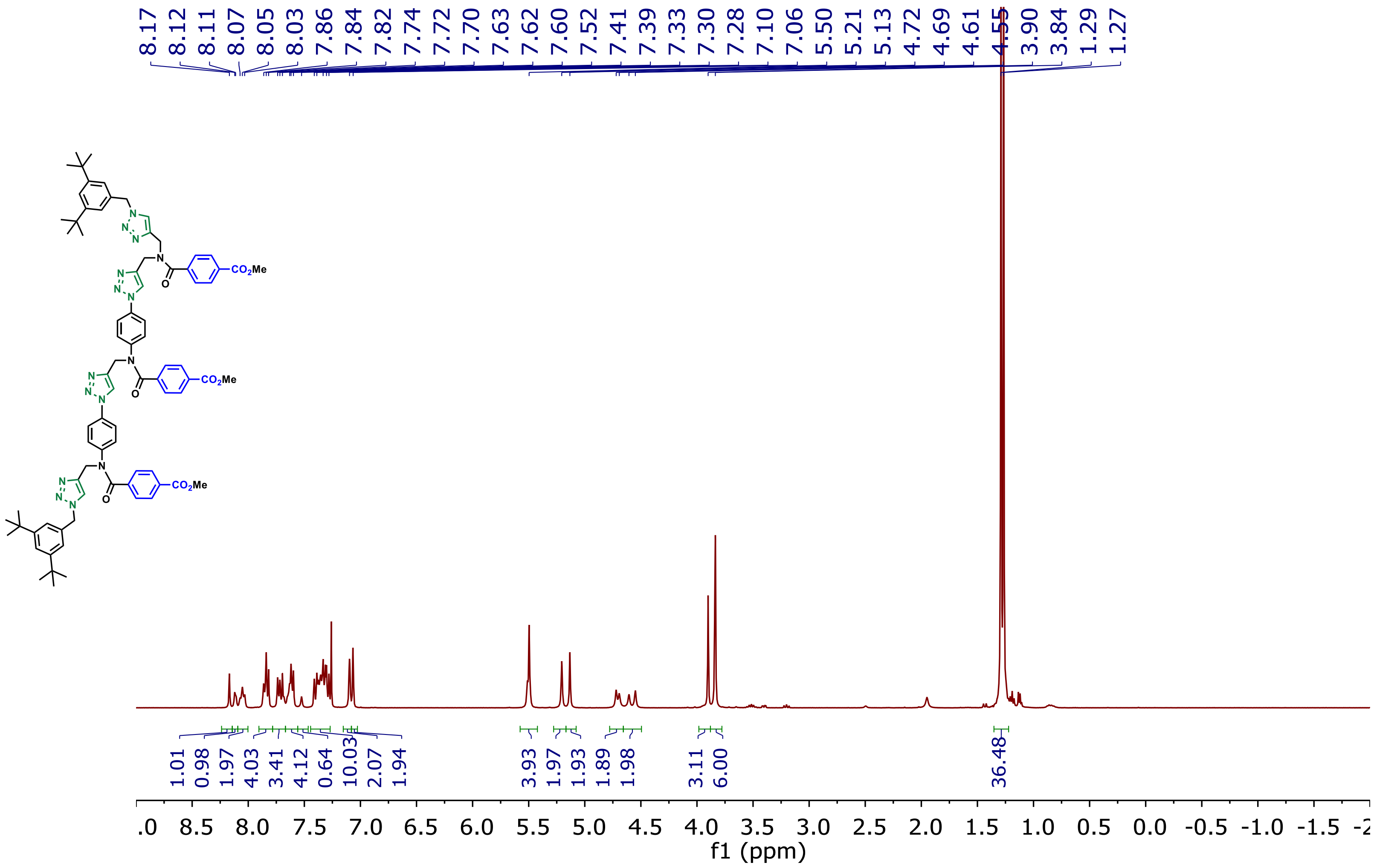

^{13}C-NMR (100.6 MHz, $CDCl_3$) Compound S7.

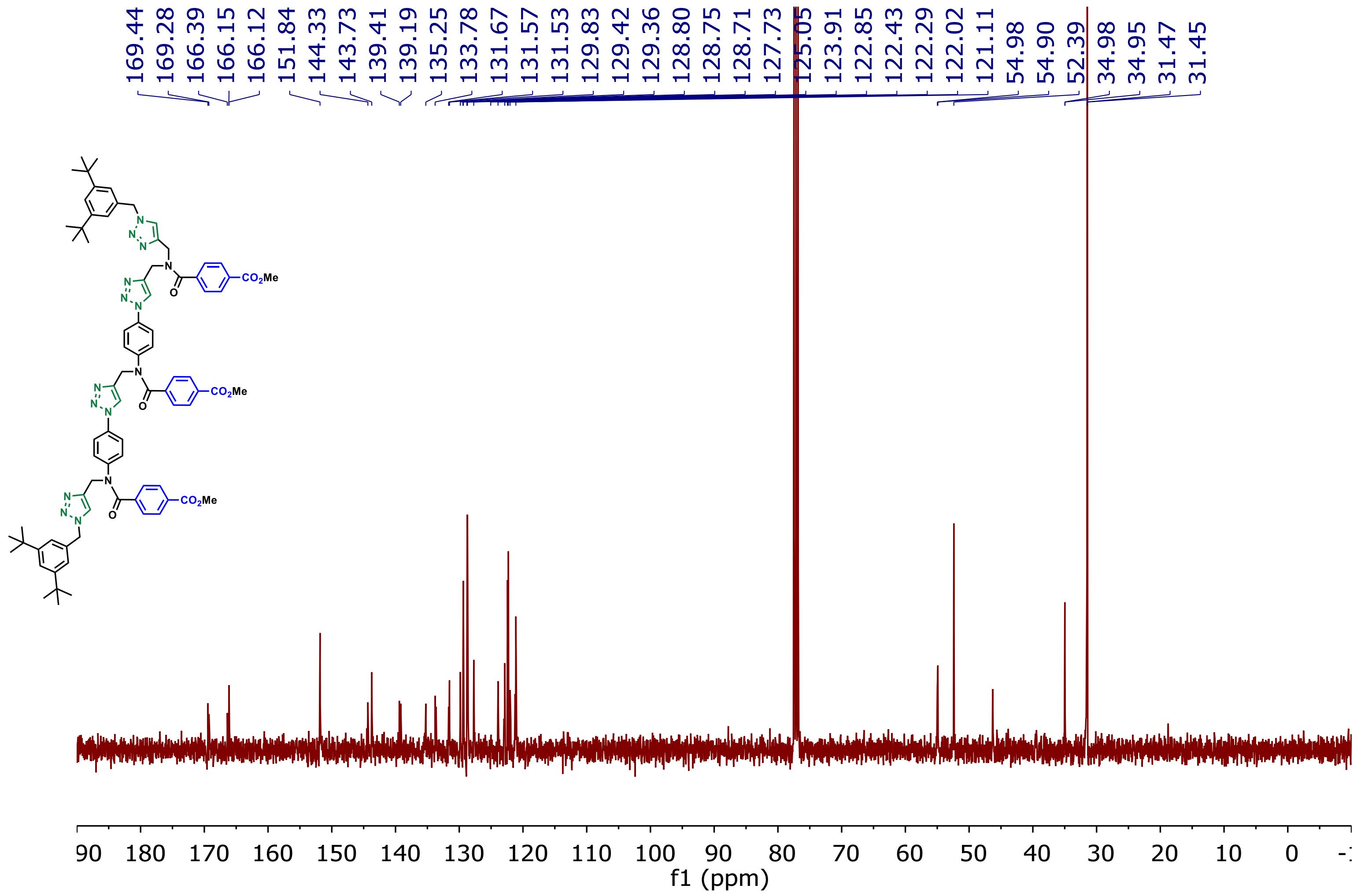

Compound 12.

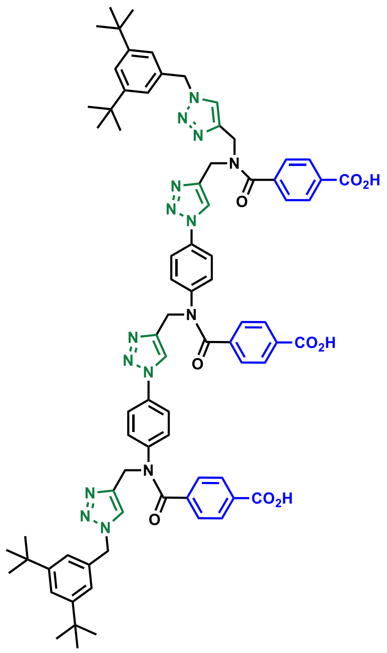

Compound **S7** (0.057 g, 0.040 mmol) was dissolved in THF/H_2O 4:1 (2.5 mL) and LiOH (0.015 g, 0.363 mmol) was added. After 3 h of stirring at room temperature, the crude diluted with H_2O and acidified with 1M HCl soln. to pH 2-3. The aqueous phase was extracted with EtOAc (3x) and the combined organic phase was washed with brine, dried over $MgSO_4$ and evaporated to dryness. The obtained yellow solid, compound **12** (0.055 g, quantitative), was used without further purification.

^{1}H NMR (500 MHz, DMSO-*d*$_6$): δ_H = 8.74 (s, 1H), 8.74 and 8.69 (s, 1H, rotamers), 8.17 (s, 2H), 7.96 (m, 2H, rotamers), 7.65 (m, 8H), 7.61 (m, 2H, rotamers), 7.45 (m, 6H, rotamers), 7.36 (d, 2H, *J* = 8.5 Hz), 7.34 (t, 1H, *J* = 2.0 Hz), 7.27 (t, 1H, *J* = 2.0 Hz), 7.13 (bs, 2H), 7.03 (d, 2H, *J* = 1.5 Hz), 5.55 (s, 2H), 5.52 (s, 2H), 5.22 (s, 2H), 5.16 (s, 2H), 4.68 and 4.65 (s, 2H, rotamers), 4.46 and 4.44 (s, 2H, rotamers), 1.24 (s, 18H), 1.15 (s, 18H).

^{13}C NMR (500 MHz, DMSO-*d*$_6$): δ_C = 169.8, 168.6, 166.7, 166.6, 150.8, 150.7, 144.2, 142.9, 142.6, 142.5, 139.8, 139.8, 139.7, 135.8, 135.3, 134.7, 134.6, 131.7, 131.7, 129.7, 129.5, 129.4, 129.2, 129.0, 129.0, 128.9, 128.9, 128.7, 128.6, 128.5, 128.4, 128.0, 127.9, 127.6, 127.1, 127.0, 126.6, 124.0, 123.9, 121.9, 121.7, 121.6, 121.5, 120.5, 120.3, 53.4, 53.2, 45.2, 45.1, 43.6, 43.5, 34.5, 34.4, 31.2, 31.1.

HRMS (ES+): calcd for $C_{78}H_{82}N_{15}O_9$ 1372.6420 $[M+H]^+$, found 1372.6370 $[M+H]^+$.

FT-IR (ATR): ν_{max} 2955, 2921, 2852, 1709, 1643, 1518, 1246, 1046, 1022, 990 and 711 cm^{-1}.

^{1}H-NMR (500 MHz, DMSO-d_6) Compound 12.

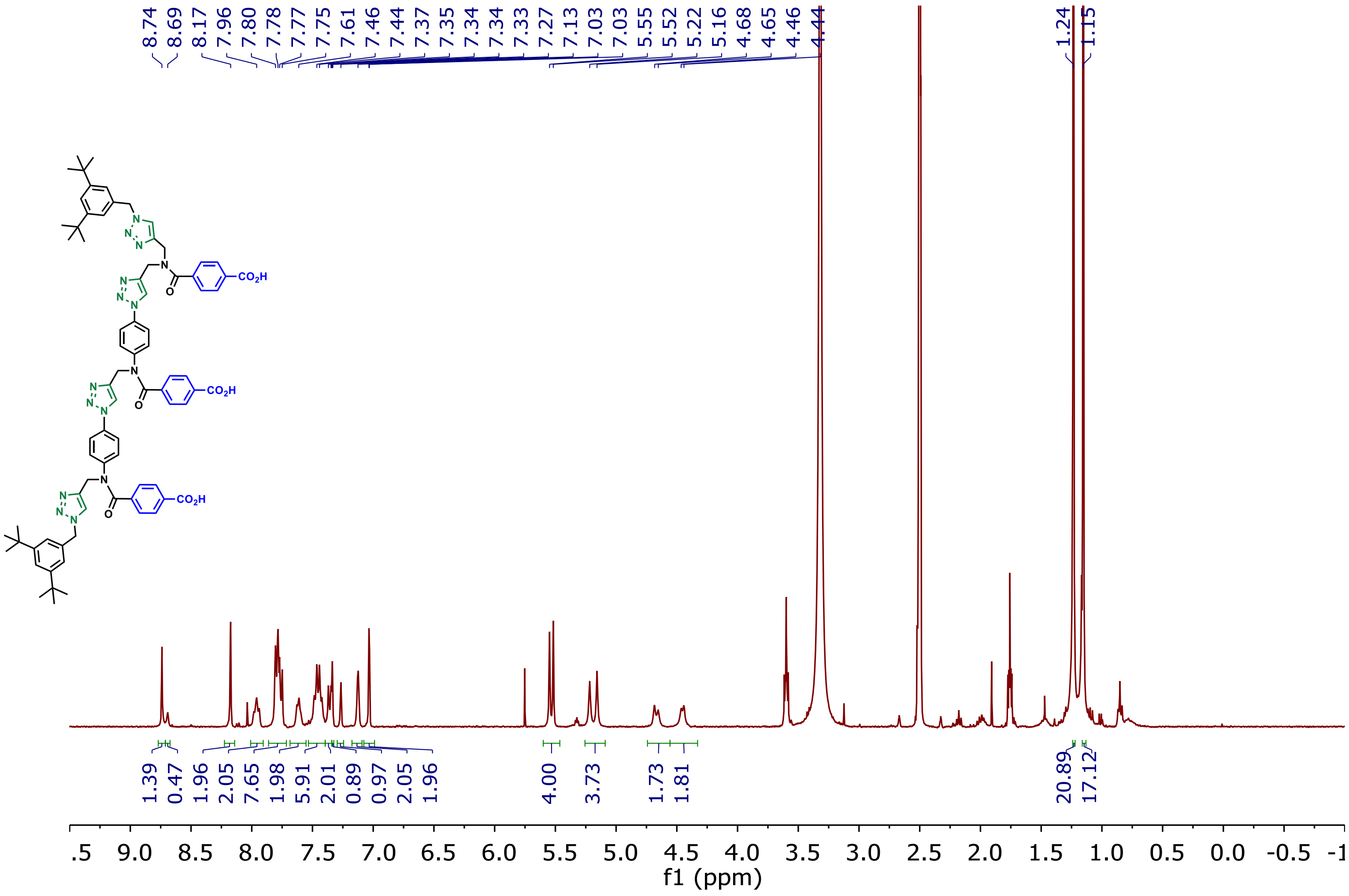

^{13}C-NMR (126 MHz, DMSO-d_6) Compound 12.

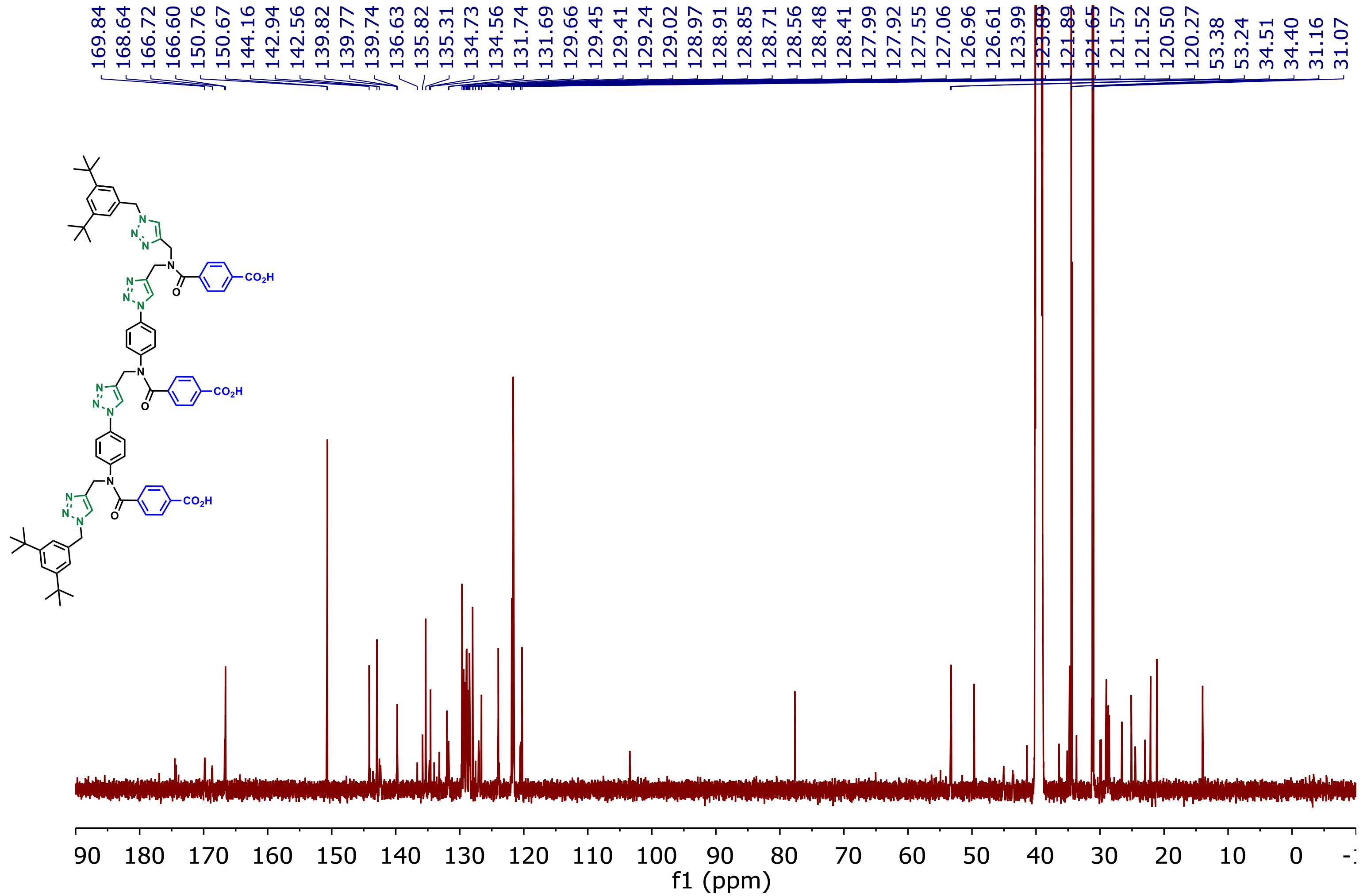

Pre-ZIP 1.

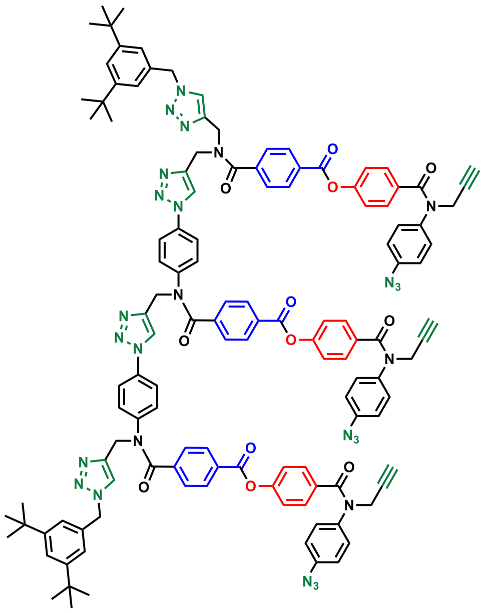

A solution of compound **12** (0.052 g, 0.04 mmol), **13**[S2] (0.044g, 0.15 mmol), EDC (0.036 g, 0.19 mmol) and DMAP (0.023 g, 0.19 mmol) in dry CH_2Cl_2 (2 mL) under N_2 atmosphere was stirred at room temperature for 16 h. The reaction was diluted with EtOAc and washed with 0.1N HCl soln. (3x), H_2O (1x) and brine. The organic phase was dried with $MgSO_4$ and concentrated *in vacuo*. The crude material was purified by flash column chromatography on silica gel (gradient from 0% to 4% of MeOH in CH_2Cl_2) to afford **1** (0.049 g, 59 %) as a light-yellow foam.

^{1}H NMR (500 MHz, $CDCl_3$): δ_H = 8.17 (m, 4H), 7.98 (m, 2H), 7.95 (d, 2H, *J* = 8.5 Hz), 7.81 (d, 2H, *J* = 8.5 Hz), 7.71 (s, 1H), 7.68 (m, 2H), 7.64 (d, 2H, *J* = 8.5 Hz), 7.56 and 7.44 (s, 1H, rotamers), 7.40 (m, 14H), 7.33 (d, 2H, *J* = 9.0 Hz), 7.15 (d, 2H, *J* = 8.5 Hz), 7.12 (m, 6H), 7.08 (m, 4H), 7.02 (m, 4H), 6.95 (d, 2H, *J* = 8.5 Hz), 6.91 (d, 4H, *J* = 8.5 Hz), 5.53 (s, 2H), 5.51 (s, 2H), 5.22 (s, 2H), 5.15 (s, 2H), 4.72-457 (m, 10H, rotamers), 2.27 (t, 1H, *J* = 2.5 Hz), 2.26 (m, 2H), 1.30 (s, 18H), 1.28 (s, 18H).

^{13}C NMR (126 MHz, $CDCl_3$): δ_C = 169.2, 169.2, 169.1, 164.0, 163.7, 163.7, 152.1, 152.1, 152.0, 152.0, 144.4, 143.8, 143.7, 140.5, 140.3, 140.1, 139.5, 139.5, 139.4, 139.4, 139.3, 135.4, 133.8, 133.7, 132.7, 132.7, 130.7, 130.6, 130.6, 130.5, 130.1, 130.0, 129.3, 129.1, 129.0, 128.9, 128.1, 124.0, 123.1, 123.0, 122.5, 122.4, 122.2, 121.4, 121.3, 121.3, 121.2, 120.1, 120.0, 78.9, 78.8, 72.8, 55.1, 55.0, 46.5, 46.4, 44.3, 43.8, 40.2, 40.1, 39.6 and 39.4, 35.1, 35.0, 31.5.

HRMS (ES+): calcd for $C_{126}H_{113}N_{27}O_{12}$ 1098.4548 $[M+2H]^{2+}$, found 1098.9215 $[M+2H]^{2+}$.

FT-IR (ATR): ν_{max} 2957, 2921, 2851, 2126, 2095, 1711, 1647, 1505, 1363, 1262 and 1166 cm^{-1}.

^{1}H-NMR (500 MHz, $CDCl_3$) Compound 1.

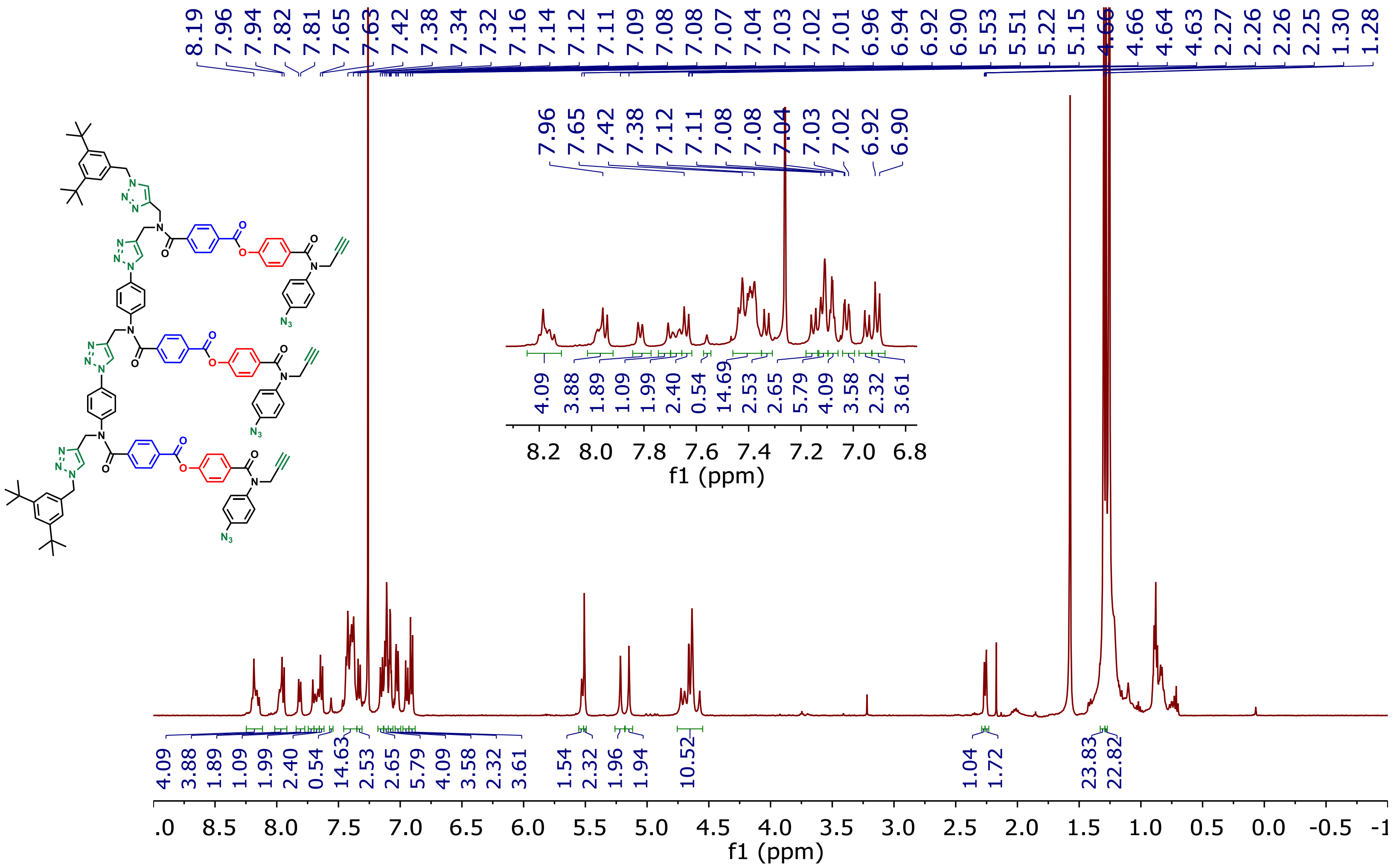

^{13}C-NMR (126 MHz, $CDCl_3$) Compound 1.

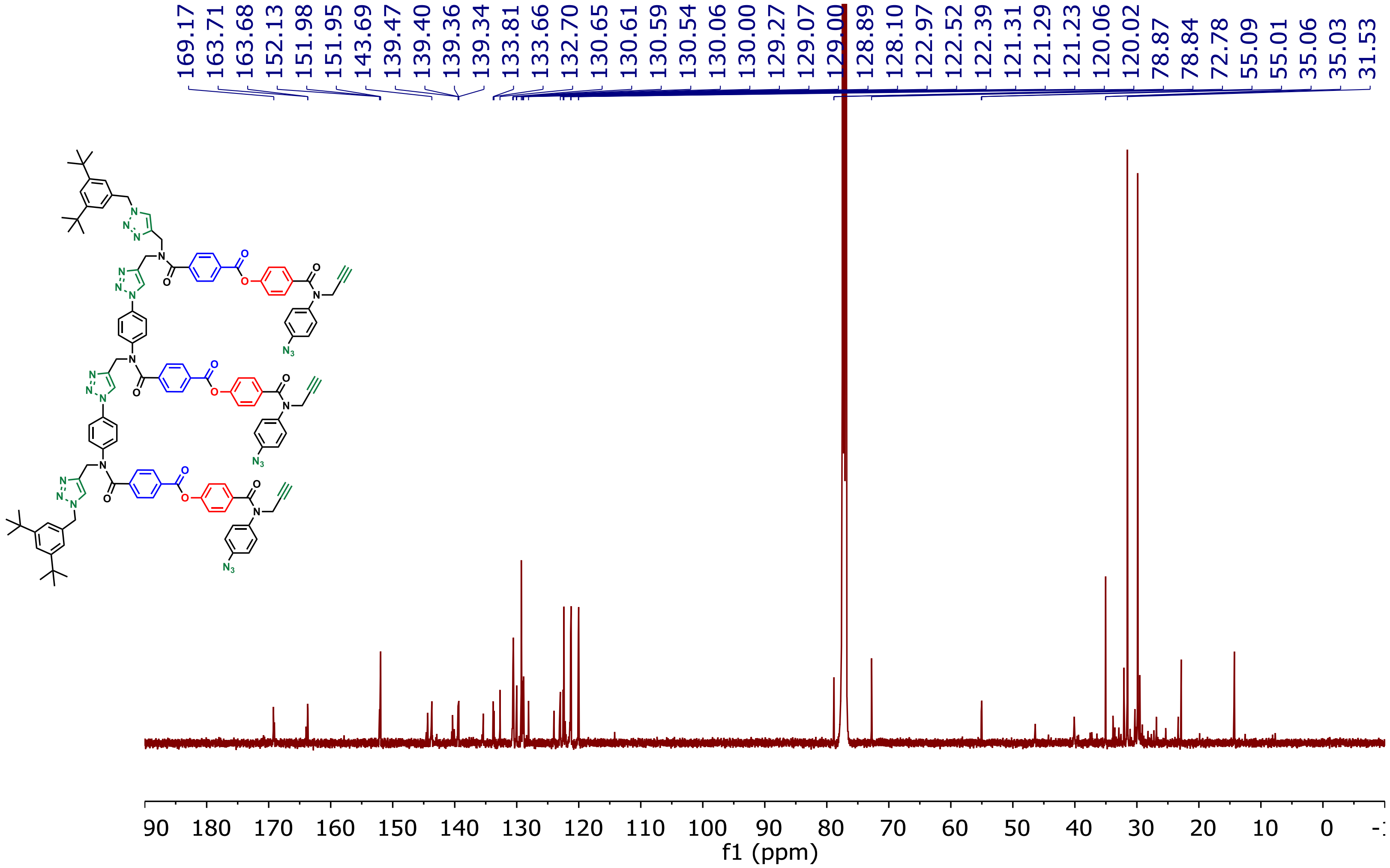

Cyclic 3-mer duplex 6.

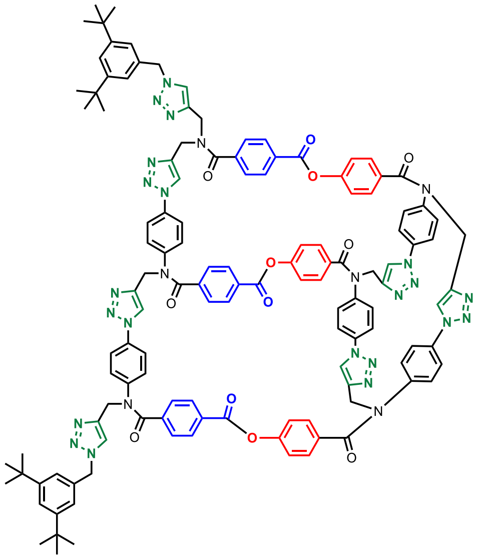

Compound **1** (0.015 g, 6.8·10^{-3} mmol) was dissolved, under N_2 atmosphere, in dry THF (700 mL). A solution of $Cu(CH_3CN)_4PF_6$ (0.010 g, 0.03 mmol) and TBTA (0.014 g, 0.03mmol) in THF (2 mL) was added to the reaction. The reaction was stirred under N_2 atmosphere at room temperature for 2 days. Then, the solvent was evaporated and the crude dissolved in EtOAc. The organic layer was washed with 0.02 M EDTA soln. (3x) and brine, then dried over $MgSO_4$. The solvent was evaporated and the crude was purified by flash column chromatography on silica gel (gradient from 0% to 7% of MeOH in CH_2Cl_2) to afford **6** (0.003 g, 16%) as a foam. Due to the complexity of the structure and the low amount of material recovered, this compound was characterized only by LCMS and IR. Further confirmation of the cyclic structure was obtained by the hydrolysis of **6** and characterization of the cyclic 3-mer **15**.

LCMS: C18 column at 40 °C (254 nm) using water + 0.1% formic acid (A) and CH_3CN + 0.1% formic acid (B) with flow rate of 0.6 ml/min; Gradient of 0-2 min 65% -100%B + 1 min 100% B.

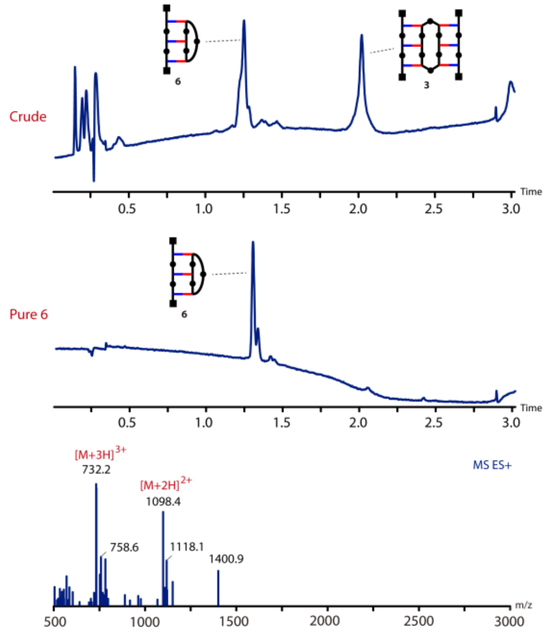

FT-IR (ATR): ν_{max} 2956, 2922, 2852, 1739, 1646, 1519, 1363, 1263, 1205 and 845 cm^{-1}.

Linear 3-mer duplex 7.

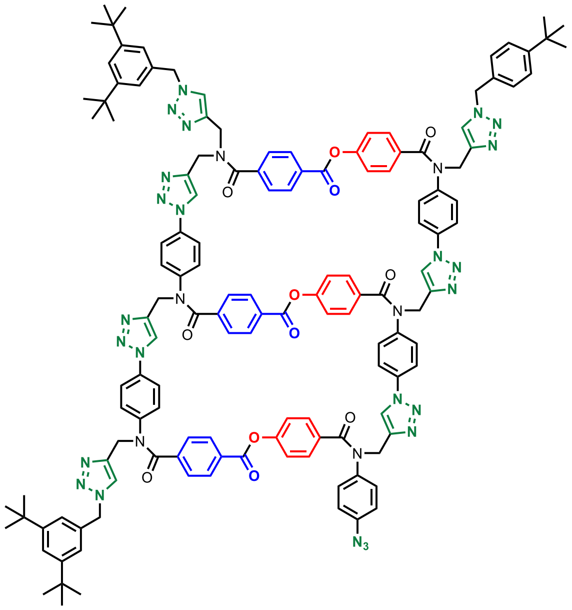

(antiparallel shown; parallel and 1,3-adduct are observed in minor proportion)

Compound **1** (0.021 g, 9.6·10^{-3} mmol) was dissolved, under N_2 atmosphere, in dry THF (380 mL). 4-*t*-Butylbenzyl azide[S4] (0.136 g, 0.72 mmol) was dissolved in THF (2 mL) and added to the reaction flask. A solution of $Cu(CH_3CN)_4PF_6$ (0.007 g, 0.02 mmol) and TBTA (0.010 g, 0.02 mmol) in THF (2 mL) was added to the reaction and the solution was stirred under N_2 atmosphere at room temperature for 2 days. Then, the solvent was evaporated and the crude dissolved in EtOAc. The organic layer was washed with 0.02 M EDTA soln. (3x) and brine, then dried over $MgSO_4$. The solvent was evaporated to dryness and the crude was purified by flash column chromatography on silica gel (gradient from 0% to 10% of MeOH in CH_2Cl_2) to afford **7** (0.016 g, 70%) as a foam.

^{1}H NMR (500 MHz, DMSO-*d*$_6$): δ_H = 8.80 (m, 4H), 8.19 (m ,2H), 8.11 (s, 1H), 8.05 (m, 2H), 7.92 (m, 2H), 7.79 (m, 10H), 7.65 (m, 2H), 7.50-7.20 (m, 28H), 7.09 (m, 8H), 6.97 (d, 2H, *J* = 8.5 Hz), 5.55 (s, 2H), 5.53 (s, 2H), 5.49 (s, 2H), 5.16 (m, 10H), 4.68 and 4.62 (s, 2H, rotamers), 4.50 and 4.43 (s, 2H, rotamers), 1.24 (m, 27H), 1.18 (m, 18H).

^{13}C NMR (126 MHz, DMSO-*d*$_6$, multiple peaks appears as broad peak due to overlapping): δ_C =168.6, 168.4, 163.8, 163.6, 151.1, 150.8, 150.8, 150.5, 143.1, 143.0, 142.4, 141.0, 135.4, 134.8, 133.3, 130.0, 129.7, 129.5, 129.2, 128.8, 127.4, 127.3, 125.4, 124.3, 124.1, 122.0, 121.8, 121.6, 120.5, 120.3, 119.8, 53.5, 53.3, 52.4, 45.0, 44.9, 43.6 and 38.7, 34.6, 34.5, 31.2, 31.2, 31.1.

HRMS (ES+): calcd for $C_{137}H_{127}N_{30}O_{12}$ 1192.5164 $[M+2H]^{2+}$, found 1192.5170 $[M+2H]^{2+}$.

FT-IR (ATR): ν_{max} 2956, 2921, 2851, 2125, 2093, 1743, 1647, 1519, 1261, 1204 and 1069 cm^{-1}.

^{1}H NMR (500 MHz, DMSO-d_6) compound 7.

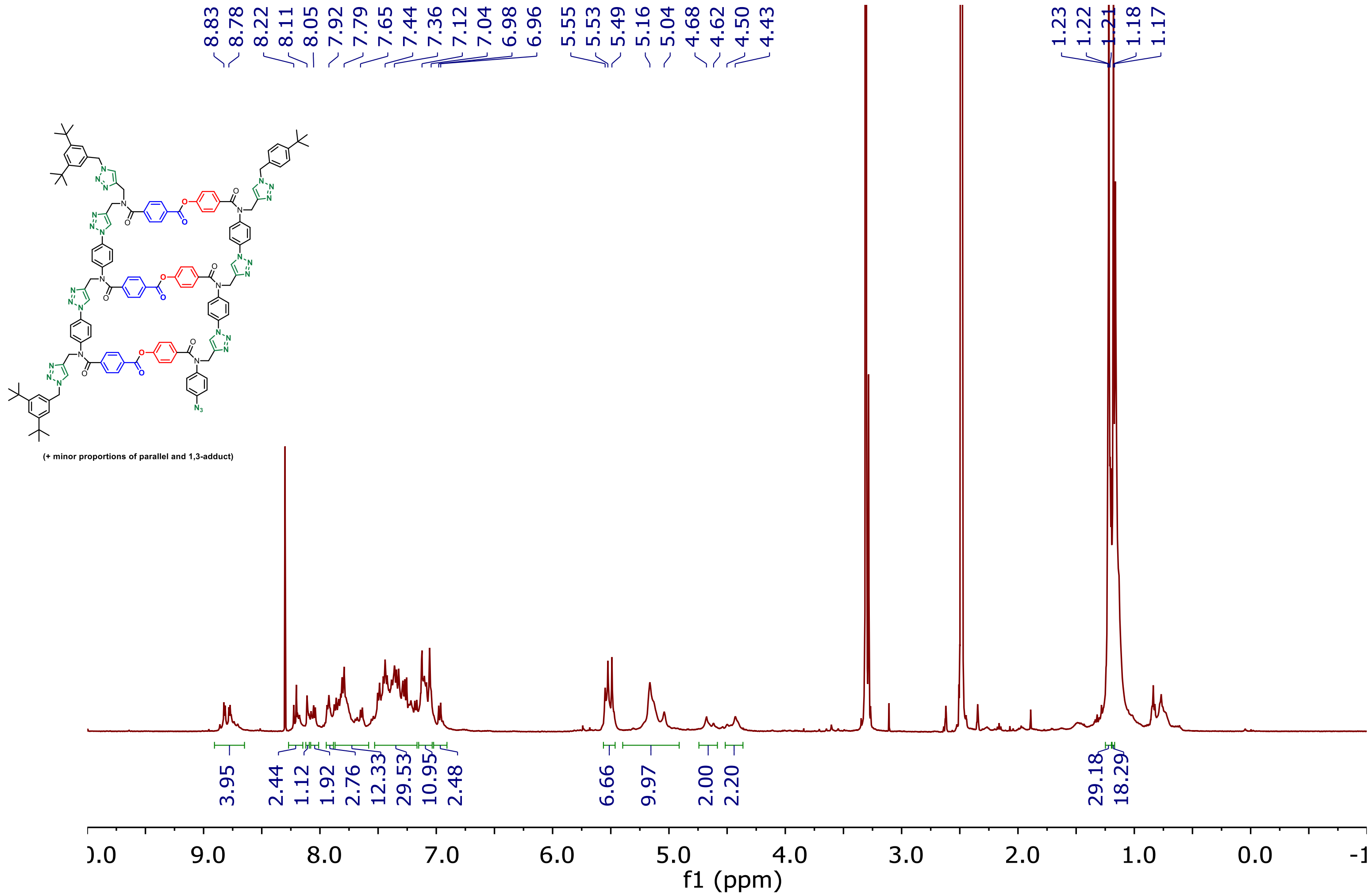

^{13}C NMR (126 MHz, DMSO-d_6) compound 7.

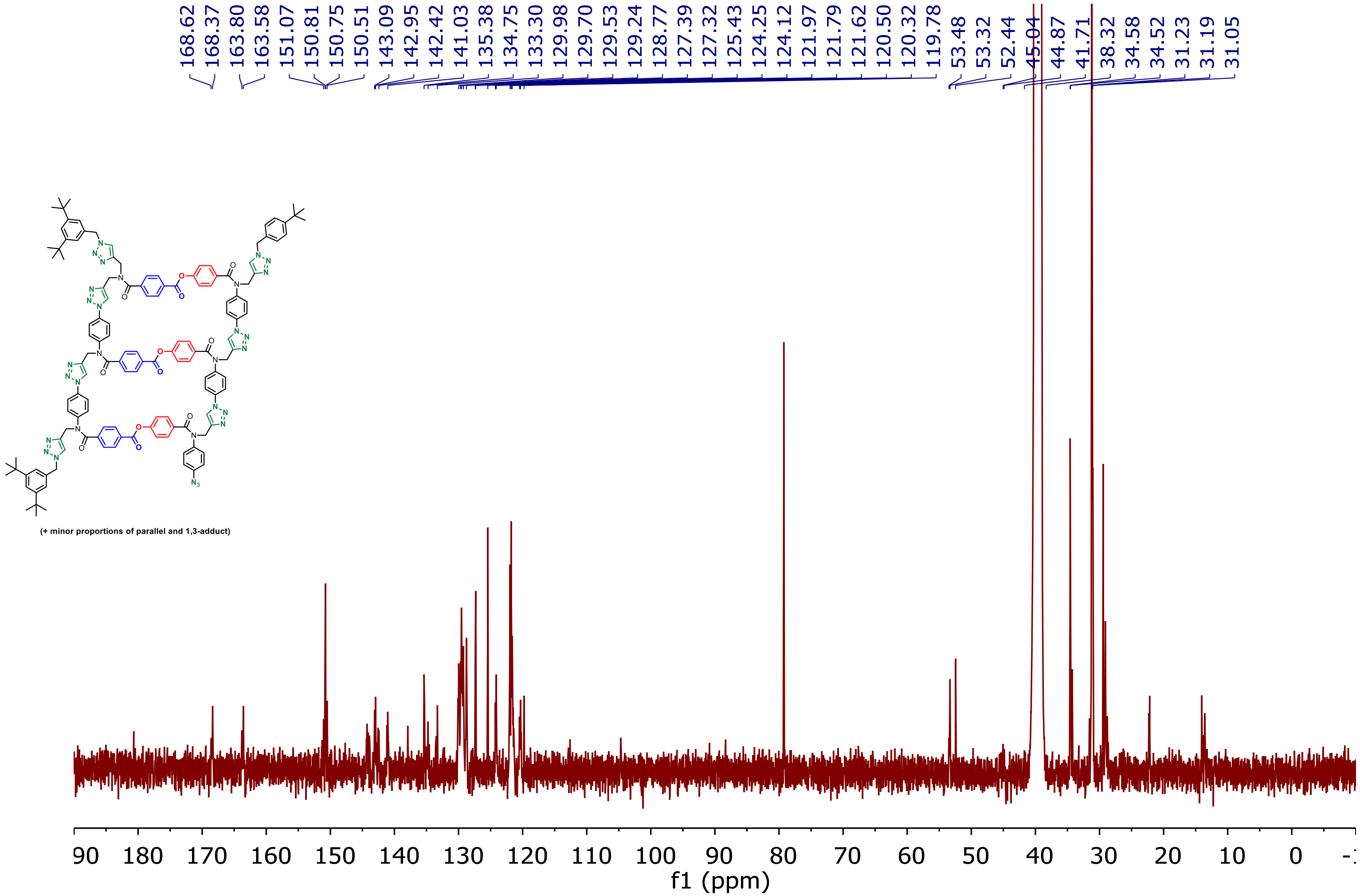

Cyclic 3-mer 15.

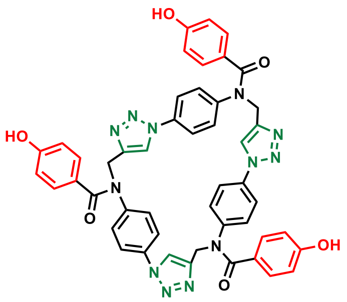

6 (2.4 mg, 0.004 mmol) was dissolved in THF/H_2O (2:1, 1.5 mL) and 1M LiOH soln. (0.05 mL, 0.05 mmol) was added and the solution stirred at room temperature for 30 min. Then, the solution was quenched with 1M HCl soln. to pH= 3-4 and diluted with EtOAc. The organic layer was washed with H_2O and brine and dried over $MgSO_4$. The solvent was evaporated to dryness and the crude was purified by flash column chromatography on silica gel (gradient from 0% to 15% of MeOH in CH_2Cl_2) to afford cyclic 3-mer **15** (0.8 mg, 83%) as a white solid.

^{1}H NMR (500 MHz, DMSO-*d*$_6$): δ_H =9.87 (bs, 3H), 8.28 (s, 3H), 7.55 (d, 6H, *J* = 9.0 Hz), 7.24 (d, 6H, *J* = 8.5 Hz), 7.20 (d, 6H, *J* = 9.0 Hz), 6.61 (d, 6H, *J* = 8.5 Hz), 5.30 (s, 6H).

^{13}C NMR (126 MHz, DMSO-*d*$_6$): δ_C = 169.2, 159.0, 143.5, 141.8, 133.5, 130.8, 129.1, 125.8, 121.5, 119.4, 114.5, 43.1.

HRMS (ES+): calcd for $C_{48}H_{37}N_{12}O_6$ 877.2959 $[M+H]^+$, found 877.2950 $[M+H]^+$.

FT-IR (ATR): ν_{max} 3132, 2918, 2850, 1638, 1608, 1518, 1462, 1278, 1239, 1170 and 762 cm^{-1}.

^{1}H-NMR (500 MHz, DMSO-d_6) compound 15.

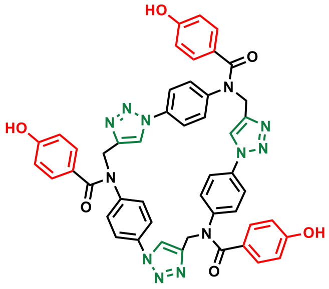

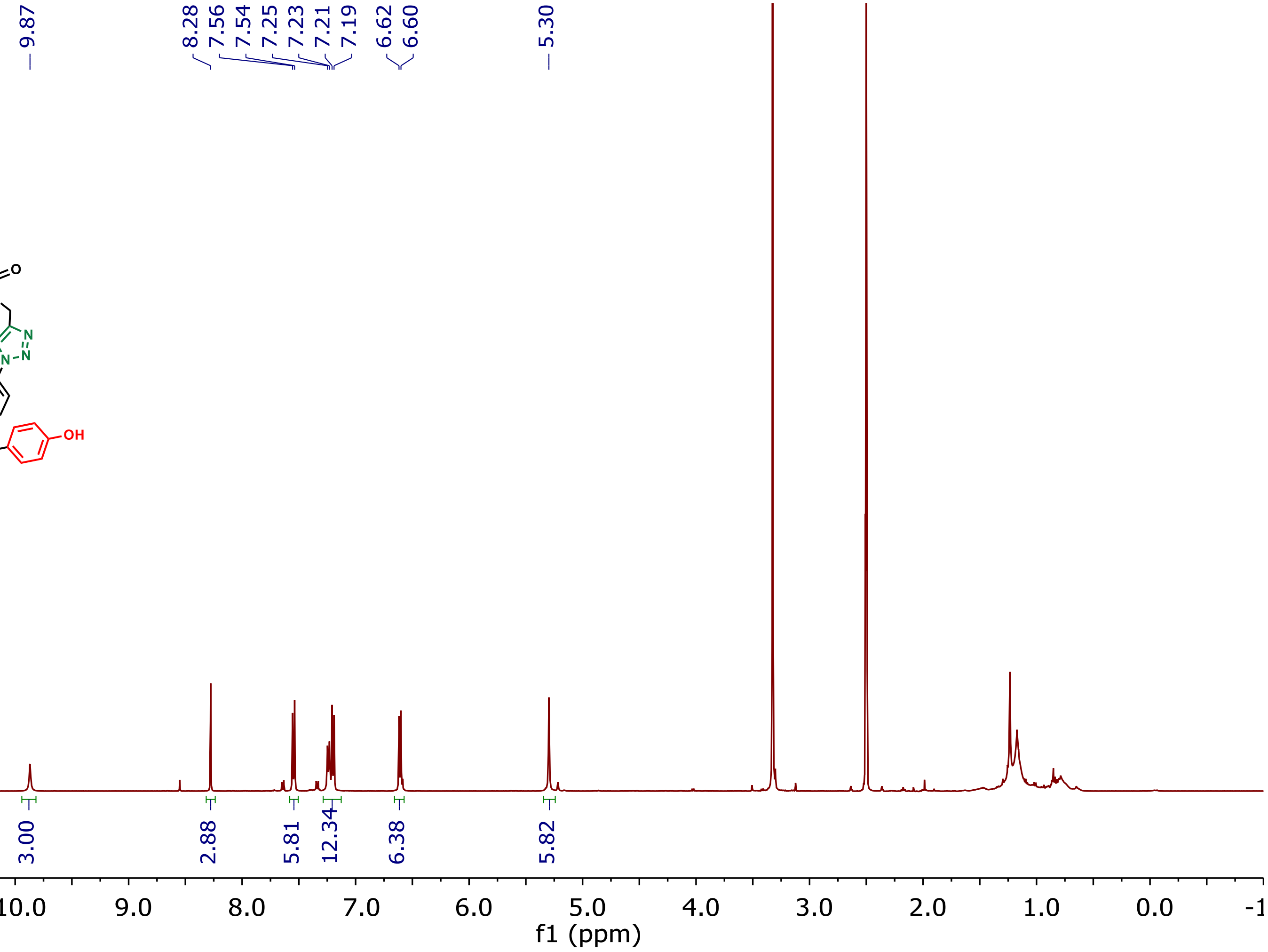

^{13}C-NMR (125 MHz, DMSO-d_6) Compound 15.

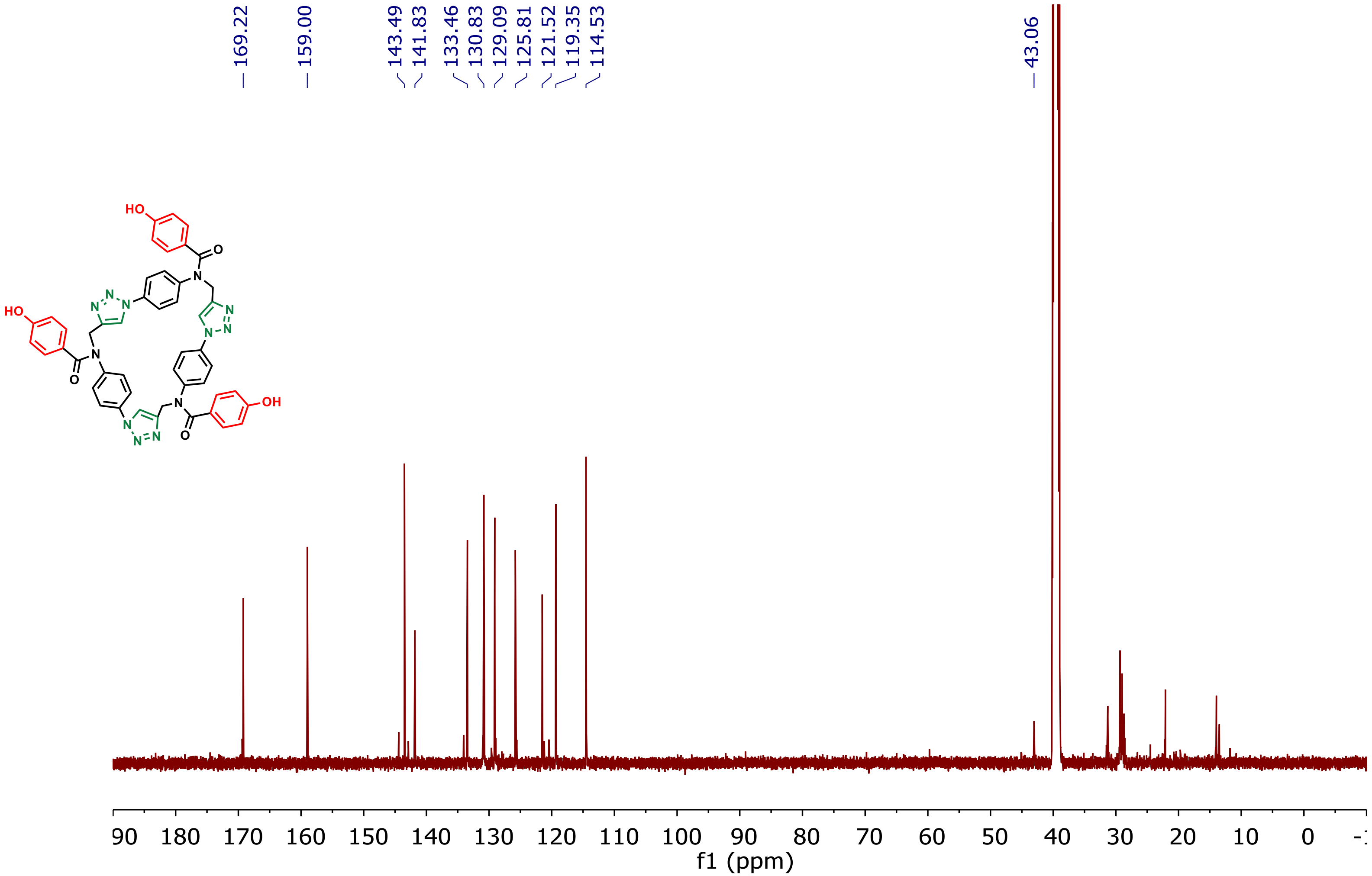

Linear 3-mer 14.

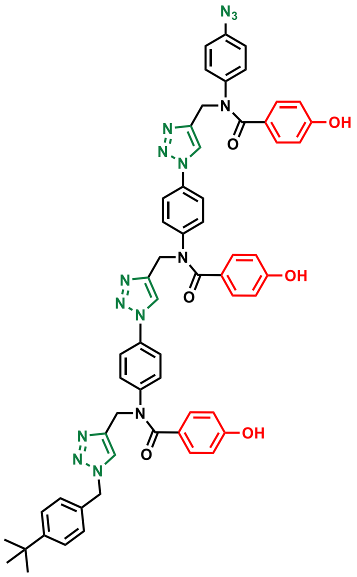

Compound **7** (0.009 g, 0.004 mmol) was dissolved in THF/H_2O (2:1, 1.5 mL) and 1M LiOH soln. (0.05 mL, 0.05 mmol) was added and the solution stirred at room temperature for 1h. Then, the solution was quenched with 1M HCl soln. to pH= 3-4 and diluted with EtOAc. The organic layer was washed with H_2O and brine and dried over $MgSO_4$. The solvent was evaporated to dryness and the crude was purified by flash column chromatography on silica gel (gradient from 0% to 10% of MeOH in CH_2Cl_2) to afford **14** (0.002 g, 50%) as a white solid.

^{1}H NMR (500 MHz, DMSO-*d*$_6$): δ_H = 9.85 (bs, 3H), 8.70 (s, 1H), 8.65 (s, 1H), 8.05 (s, 1H), 7.77 (d, 2H, *J* = 9.0 Hz), 7.77 (d, 2H, *J* = 9.0 Hz), 7.39 (d, 2H, *J* = 9.0 Hz), 7.28 (d, 2H, *J* = 8.5 Hz), 7.28 (d, 2H, *J* = 9.0 Hz), 7.20 (d, 2H, *J* = 9.0 Hz), 7.20 (d, 2H, *J* = 9.0 Hz), 7.17 (d, 2H, *J* = 9.0 Hz), 7.15 (d, 2H, *J* = 9.0 Hz), 7.08 (d, 2H, *J* = 8.5 Hz), 6.97 (d, 2H, *J* = 9.0 Hz), 6.58 (m, 6H), 5.48 (s, 2H), 5.17 (s, 2H), 5.10 (s, 2H), 5.09 (s, 2H), 1.17 (s, 9H).

^{13}C NMR (126 MHz, DMSO-*d*$_6$): δ_C = 169.2, 169.2, 169.1, 159.0, 158.8, 150.5, 144.6, 143.7, 143.5, 143.4, 140.6, 137.3, 134.2, 134.0, 133.2, 130.9, 130.8, 130.8, 129.2, 128.8, 128.7, 127.3, 125.9, 125.7, 125.7, 125.4, 124.0, 121.5, 121.5, 120.4, 120.3, 119.6, 114.6, 114.5, 114.4, 52.4, 45.4, 45.1, 34.2, 31.0.

HRMS (ES+): calcd for $C_{59}H_{52}N_{15}O_6$ 1066.4225 $[M+H]^+$, found 1066.4205 $[M+H]^+$.

FT-IR (ATR): ν_{max} 3150, 2921, 2851, 2123, 2092, 1631, 1607, 1516, 1278, 1237, 1169 and 844 cm^{-1}.

^{1}H-NMR (500 MHz, DMSO-d_6) Compound 14.

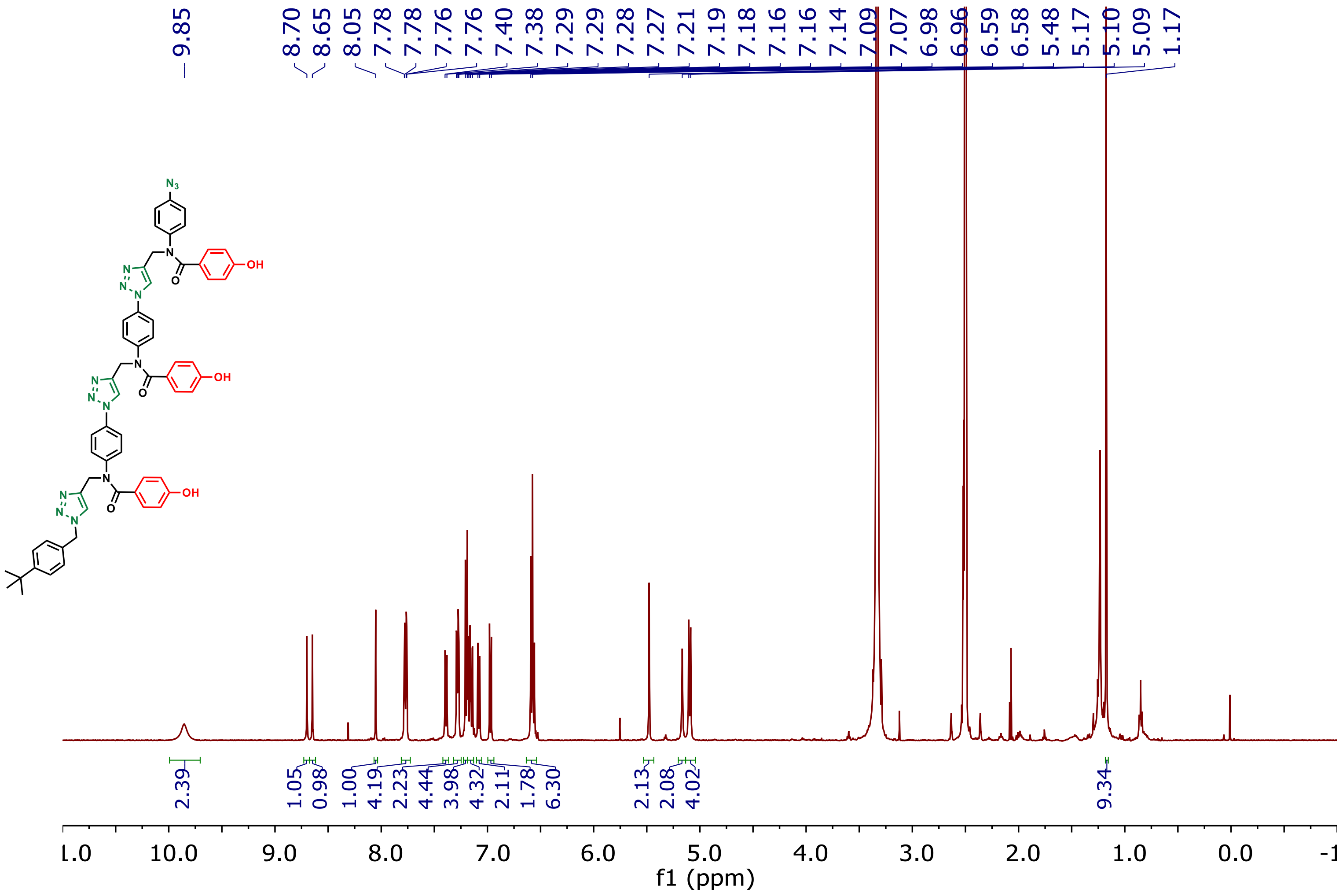

^{13}C-NMR (125 MHz, DMSO-d_6) Compound 14.

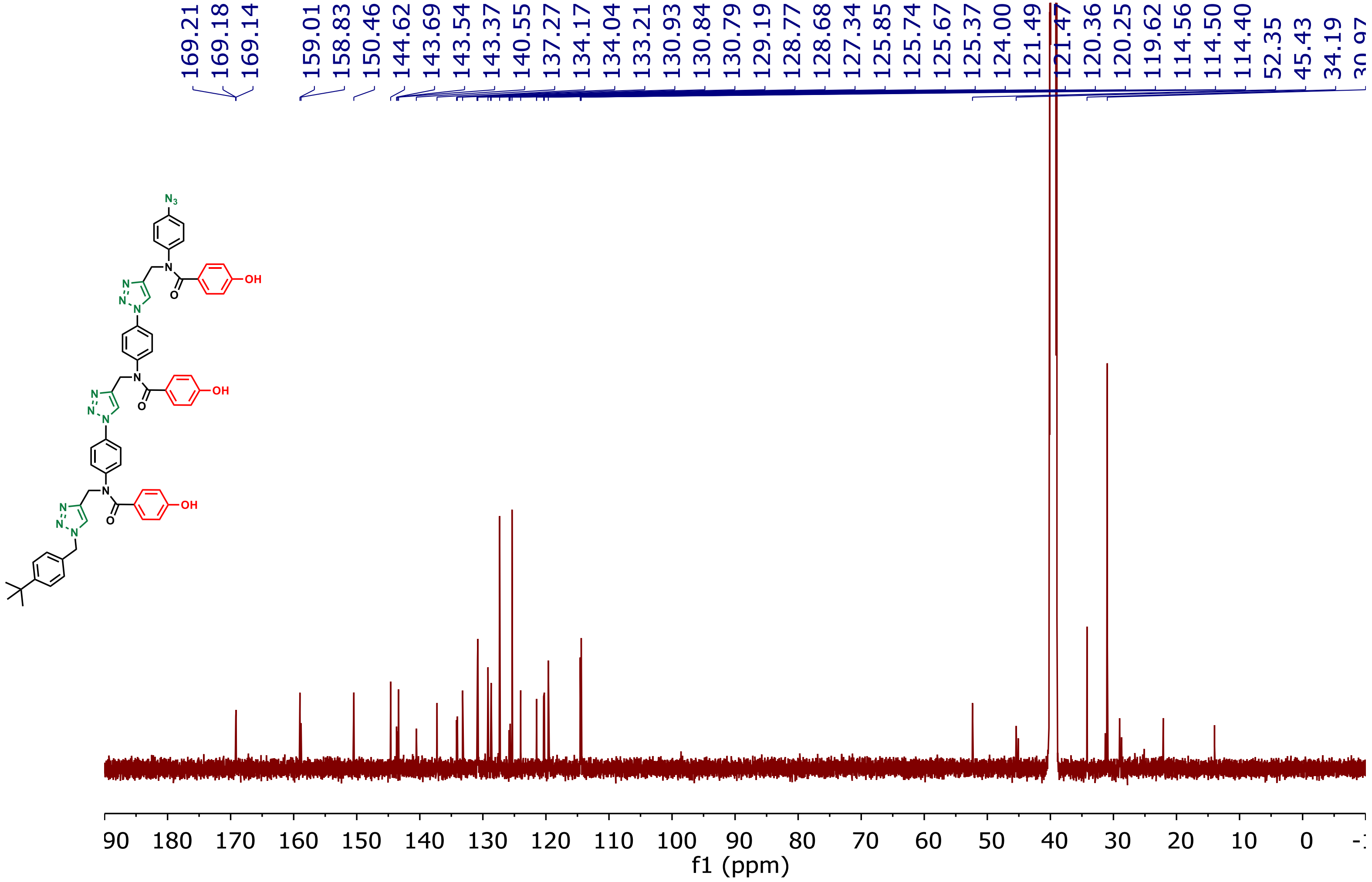

References

[S1] M. Ciaccia, D. Núñez-Villanueva, C. A. Hunter, *J. Am. Chem. Soc.* **2019**, *141*, 10862.

[S2] D. Núñez-Villanueva, M. Ciaccia, G. Iadevaia, E. Sanna, C. A. Hunter. *Chem. Sci.* **2019**, *10*, 5258.

[S3] V. Vanek, J. Pícha, B. Fabre, M. Budesínský, M. Lepsík, J. Jirácek. *Eur. J. Org. Chem.* **2015**, 3689.

[S4] J. J. Gassensmith, L. Barr, J. M. Baumes, A. Paek, A. Nguyen and B. D. Smith. *Org. Lett.* **2008**, *10*, 3343.
